# DHX9-mediated epigenetic silencing of BECN1 contributes to impaired autophagy and tumor progression in breast cancer via recruitment of HDAC5

**DOI:** 10.1038/s41419-025-07847-y

**Published:** 2025-07-14

**Authors:** Ziyang Li, Fang Liu, Fengbei Li, Guopeng Zeng, Xin Wen, Jianan Ding, Jueyu Zhou

**Affiliations:** Department of Biochemistry and Molecular Biology, School of Basic Medical Sciences, Southern Medical University, Guangzhou, China

**Keywords:** Breast cancer, Autophagy, Gene silencing

## Abstract

Autophagy is closely linked to tumorigenesis, progression and metastasis. DHX9 is a member of the DExD/H-box helicase family and plays important roles in transcription, translation, RNA editing and non-coding RNA synthesis. Mounting evidence demonstrates that aberrant expression of DHX9 is associated with the development and progression of several tumors. However, whether DHX9 regulates autophagy deficiency in breast cancer (BC) remains unknown. Herein, we found that DHX9 expression was frequently elevated in BC cells and tissues, which suggested poor survival. The viability and motility of BC cells were irritated by enhanced DHX9 expression. Meanwhile, reduced DHX9 expression postponed tumor development both in vitro and in vivo. Subsequent research revealed that DHX9 knockdown suppressed the activation of the mTOR signaling pathway and accelerated autophagic flux by promoting the formation of autophagosomes in BC cells. Mechanistically, DHX9 occupied the proximal promoter of BECN1 and repressed its transcription. DHX9-mediated BECN1 inhibition required histone deacetylase (HDAC) activity. HDAC5 was recruited to the nucleus and co-localized with DHX9 at the BECN1 promoter, mediating the deacetylation of histone H3 and ultimately inhibited BECN1 transcription. Importantly, the tumor-suppressive effect of DHX9 knockdown was reversed by BECN1 downregulation. In conclusion, the previously unrecognized significance of DHX9 in mediating the epigenetic silencing of BECN1, which is essential for autophagy and tumorigenesis, highlights its potential as an effective biomarker as well as a prospective therapeutic candidate for BC.

## Introduction

Breast cancer (BC) currently ranks first in both incidence and mortality among women in the worldwide [1]. BC prognosis exhibits high heterogeneity across different diagnostic stages, molecular subtypes and other clinicopathological characteristics. The 5-year survival rates reach 79%–99% for locoregional stage tumors but drop to 24%–39% in distant stage. Among molecular subtypes, hormone receptor (HR) positive/ human epidermal growth factor receptor-2 (HER2) negative BCs show the highest survival probability (89%–96%), whereas triple-negative breast cancer (TNBC) has a survival probability of 73%–81% [2]. Surgery combined with adjuvant therapy remains central to BC treatment. Precision approaches including endocrine, targeted therapies and novel agents (cell cycle inhibitors, immunotherapies) have improved outcomes. However, molecular heterogeneity, unclear mechanisms, and drug resistance continue to pose major challenges.

In BC, experimental evidence indicates that autophagy has a considerable impact on the growth, progression of tumors and the response to chemotherapy. *BECN1*, the first tumor-associated autophagy-related gene (ATG) identified in mammals, is mono-allelically deleted in BC and functions as a tumor suppressor [3, 4]. Homozygous-BECN1-deficient mice exhibit embryonic lethality, while heterozygous disruption of BECN1 leads to the occurrence of lung cancer, liver cancer, lymphoma and mammary tumor [4, 5]. Thus, BC is genetically related to impaired autophagy.

DHX9 belongs to the DExH-box helicase family. It is a part of the helicase superfamily 2 together with the DExD-box family [6]. DHX9 is capable of unraveling complex polynucleotide structures [7], and plays a multifunctional role in regulating transcription [8, 9], translation [10]. Accumulating studies have recently revealed that DExD/H-box helicases exert their biological functions through interactions with ATGs to affect autophagy processes [11]. Nevertheless, a specific role for DHX9 in autophagy has yet to be established.

Increasing evidence has demonstrated DHX9 dysregulation is linked to diseases. DHX9, mainly exerting oncogenic roles, is aberrantly expressed in several cancers. For instance, DHX9 stimulates cell viability, motility and apoptosis resistance in colorectal cancer by activating the NF-κB signaling pathway [12]. One research identified DHX9 as a key node involved in cell proliferation and the mTOR signaling pathway in TNBC [13]. The upregulated long-stranded non-coding RNA AK023948, triggered the AKT signaling pathway by interacting with DHX9 in MCF7 cells [14]. Nevertheless, whether abnormal DHX9 expression affects BC cell autophagy and its regulatory mechanisms remains unknown.

Our current work involved an investigation of DHX9 expression and prognosis significance across BC PAM50 subtypes. Subsequent experiments uncovered the oncogenic functions of DHX9 in MCF7 (Luminal A) and MDA-MB-231 (TNBC), the widely applicable BC cell models and an orthotopic xenograft model in nude mice. Furthermore, our results highlighted its potential roles and possible molecular mechanisms involved in autophagy inhibition. Collectively, our findings provide evidence that DHX9 hinders the transcription of BECN1 by recruiting HDAC5 to its promoter mediating the deacetylation of histone H3 and accelerates breast tumor progression.

## Results

### DHX9 is frequently upregulated in BC and indicates poor prognosis

**Fig. 1:**
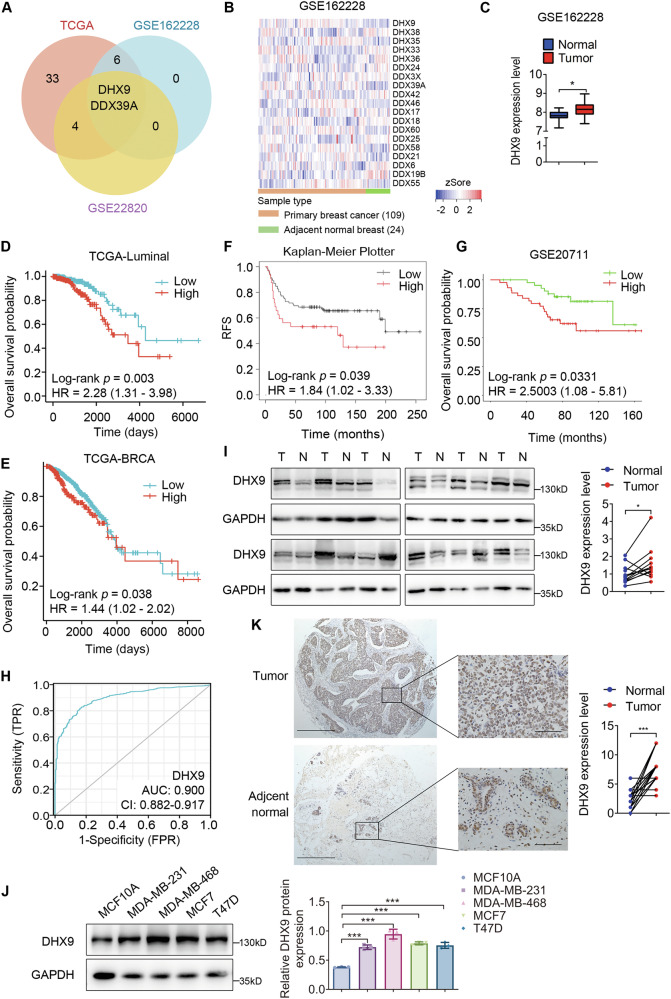
DHX9 is highly expressed in breast cancer and indicates poor prognosis. **A** Venn diagram constructed with differentially highly expressed DExH/D-box helicases in breast cancer from the TCGA-BRCA dataset and two public GEO datasets (GSE162228, GSE22820). The threshold values were established at *p* < 0.05 and log fold change>0. **B** Representative heatmap from a GEO dataset (GSE162228) showing the differentially expressed DExH/D-box helicases. The threshold value was *p* < 0.05. **C** Box diagram representing the quantitative analysis of DHX9 expression in breast cancer (*n* = 109) and adjacent breast tissues (*n* = 24) from a GEO dataset (GSE162228). **D** The difference in the overall survival probability in luminal subtypes between DHX9-high and -low group based on TCGA-BRCA survival data was calculated by the Kaplan-Meier methodology. **E** The difference in overall survival probability between DDX39A-high and -low group based on TCGA-BRCA survival data was calculated by the Kaplan-Meier method. **F**, **G** Kaplan-Meier survival plots generated using Kaplan-Meier Plotter (**F**) and OSbrca (**G**) online databases. RFS relapse-free survival. **H** Receiver operating characteristic curve indicating the potential diagnostic value of DHX9. The AUC value was 0.900 (CI = 0.882–0.917, *p* < 0.001). **I** The protein levels of DHX9 in 12 pairs of breast cancer tissues (T) and matched adjacent tissues (N) were evaluated by Western blotting. The scatter diagram (Right) depicts the relative expression of DHX9 to GAPDH. **J** The protein levels of DHX9 in four breast cancer cell lines and a normal breast epithelium cell line MCF10A were evaluated by Western blotting. Histogram (Right) shows the relative expression of DHX9 to GAPDH. **K** Representative IHC images of DHX9 expression in a pair of primary breast cancer and matched adjacent tissue from the tissue microarray were shown. Scale bars, 500 μm (Left images). The middle raw images exhibit a 10-fold magnification compared to the left ones. Scale bars, 50 μm. The scatter plot (Right) indicated the immunohistochemical staining scores of DHX9 in breast tumors (*n* = 43) and matched adjacent tissues (*n* = 38). Data are representative of three biological independent experiments (**J**) and are plotted as the mean ± SD (**I**–**K**). *P*-values were calculated by unpaired (**J**) or paired (**I, K**) two-tailed Student’s t test. *p < 0.05, ***p < 0.001 vs. corresponding control.

Given the importance of DExD/H-box helicases in many physiological and pathological processes, we interrogated the TCGA-BRCA database and two public GEO datasets (GSE162228 and GSE22820) to analyze the expression profile of the DExD/H-box helicase family. A venn diagram was subsequently created on the basis of the differentially highly expressed genes, of which two genes (*DHX9* and *DDX39A*) were identified (Fig. 1A). The representative heatmap from GSE162228 displayed all the differentially expressed DExD/H-box helicases (Fig. 1B). The box plot in Fig. 1C showed significantly higher DHX9 levels in BC tissues compared to adjacent normal tissues.

Furthermore, we analyzed the prognosis significance of genes (*DHX9* or *DDX39A*) using the TCGA-BRCA dataset. We found a strong connection between elevated DHX9 expression and worse overall survival in luminal BCs. However, this connection was not observed in TNBC and HER2-positive (HER2 + ) BCs (Fig. 1D and Figure S1A, B). Nevertheless, higher expression of DDX39A represented a better prognosis in both all BC cases and luminal subtypes (Fig. 1E and Figure S1C–E). These results implied DHX9 might function as a tumor promoter, whereas DDX39A as a tumor suppressor. Hence, we decided to examine the function of DHX9 in BC. Likewise, we generated Kaplan-Meier (KM) survival plot using the OSbrca and the KM Plotter online tool. The plots displayed patients harboring higher DHX9 levels exhibited significantly worse overall survival, relapse-free survival, distant relapse-free survival, disease-free survival and metastasis-free survival (Fig. 1F, G and Figure S1F). Meanwhile, the diagnostic efficacy of DHX9 in BC was determined through receiver operating characteristic curve analysis, indicating its potential role as a biomarker (Fig. 1H). Moreover, clinicopathological parameters unmasked a link between DHX9 expression and variables including age, race, menopausal status, PAM50 and histological type (Table S1).

Using the TCGA dataset, we observed that DHX9 mRNA levels were frequently raised in the majority of cancer types compared with adjacent normal tissues (Figure S1G, H). Furthermore, the CPTAC database also showed the higher protein levels of DHX9 in BC samples (Figure S1I). Subsequently, we examined DHX9 expression in clinical specimens collected from Nanfang Hospital and several BC cell lines via Western blotting. Higher DHX9 expression was observed in primary tumors than that in adjacent normal tissues (Fig. 1I). Consistently, DHX9 was obviously upregulated in BC cell lines compared with the mammary epithelial cell line MCF10A (Fig. 1J). IHC analysis of a commercial tissue microarray further displayed a frequently raised DHX9 expression in BC tissues compared with paired noncancerous samples, while DHX9 was largely located in the nucleus of BC cells (Fig. 1K). Taken together, our data showed that DHX9 was significantly upregulated in BC and its enhanced expression was correlated with poor prognosis.

### DHX9 knockdown represses tumorigenesis in vitro and in vivo

**Fig. 2:**
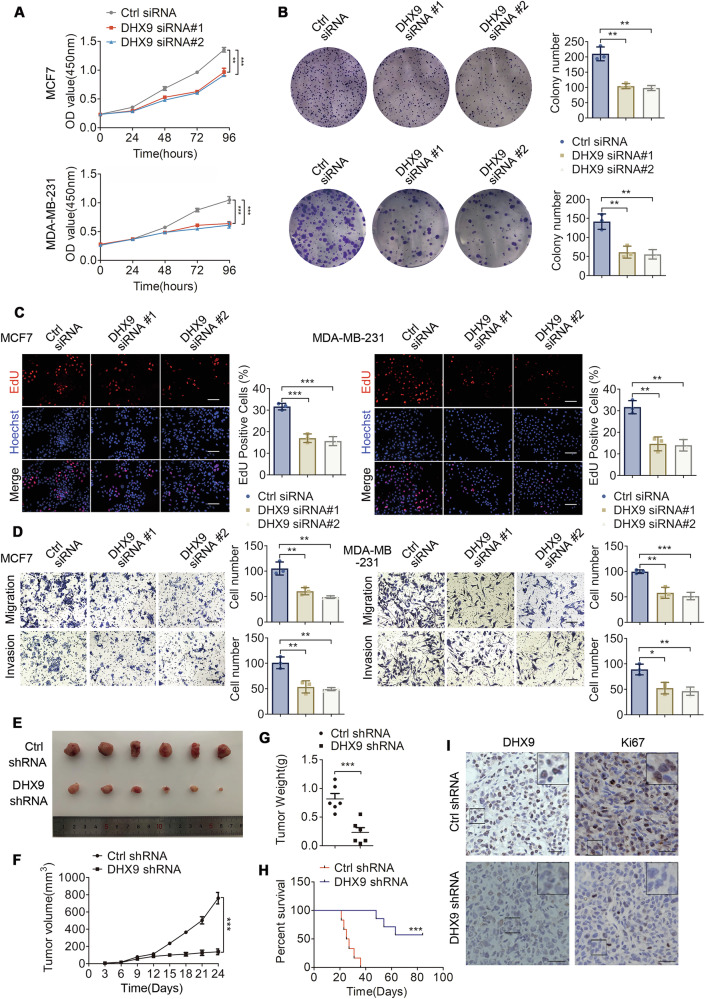
DHX9 knockdown impairs breast tumorigenesis in vitro and in vivo. **A** CCK-8 assays to evaluate the cell viability in BC cells after DHX9 knockdown. **B** Colony formation assays were performed after DHX9 knockdown. **C** EdU tests were conducted following DHX9 knockdown. Representative views and the quantification of EdU-positive cells are shown. Scale bars, 100 μm. **D** Transwell assays were conducted after DHX9 knockdown. Scale bars, 100 μm. **E** Image of excised xenografts at the terminal of the experiment (6 mice per group). **F**, **G** The volume (**F**) and weight (**G**) were calculated after three weeks growth of the xenografts (6 mice per group). **H** The Kaplan–Meier survival curve was established to display the survival difference among tumor-bearing nude mice. The statistical parameters were determined by a log-rank test (6 mice per group). ***p < 0.001. **I** Representative IHC pictures showing the immunostaining of DHX9 and Ki67 in mice xenografts (6 mice per group). Scale bars, 25 μm. Data are representative of three biological independent experiments (**A–D**) and are plotted as the mean ± SD (**A-D**, **F**, **G**). *P*-values were calculated by unpaired two-tailed Student’s t test (**A–D**, **F**, **G**). *p < 0.05, **p < 0.01, ***p < 0.001 vs. corresponding control.

To clarify the impact of DHX9 on the malignant phenotype of BC cells, DHX9 was transiently overexpressed or downregulated in MCF7 and MDA-MB-231 cells, with transfection efficacy validated via Western blotting (Figure S2A). The cell proliferation capacity was evaluated using CCK8, colony formation and EdU tests. As expected, DHX9 silencing significantly weakened cell growth and reduced the number of colonies (Fig. 2A, B). Consistently, EdU assays confirmed that DHX9 depletion in BC cells hampered cell proliferation (Fig. 2C). In contrast, DHX9 overexpression promoted cell viability and increased the number of colonies (Figure S2B, C). Then, to evaluate the potential influence of DHX9 on cell invasion and migration, we performed transwell assays. As expected, DHX9 knockdown reduced cell invasion and migration abilities (Fig. 2D), whereas DHX9 overexpression had the opposite effects (Figure S2D).

To further confirm the oncogenic function of DHX9 in vivo, the right fourth mammary fat pads were injected with MDA-MB-231 cells stably transfected with shCtrl or shDHX9 plasmids to establish a xenograft nude mouse model. Western blotting was employed to verify the effectiveness of these knockdown cell lines (Figure S2E). As expected, DHX9 knockdown obviously reduced tumor growth, volume and weight (Fig. 2E–G). However, no macroscopic metastatic lesions (including in the liver and lungs) were observed in either group and the body weight showed no significant difference between the groups. Furthermore, we documented the survival times of the mice bearing orthotopic xenograft tumors and utilized the Kaplan-Meier method for data analysis. The results showed DHX9 inhibition markedly extended the survival time of the tumor-bearing mice (Fig. 2H). Moreover, in DHX9-knockdown tumors, IHC showed that DHX9 and Ki-67 expressions were significantly reduced (Fig. 2I), while cleaved caspase-3 levels increased (Figure S2F). Collectively, we demonstrated that DHX9 played an oncogenic role in BC development, which could be a promising therapeutic target to combat the aggressive behavior of BC.

### DHX9 knockdown contributes to autophagy activity

To dissect how DHX9 affects biological behavior, GSEA analyses were run to identify the potential pathways. The results displayed that DHX9 was involved in the mammalian target of rapamycin (mTOR) signaling pathway in BC (Figure S3A). Subsequently, several proteins associated with the mTOR pathway were identified using Western blotting. Compared with those in the control group, the protein expressions levels of p-mTOR, p-RPS6 and p-AKT were significantly upregulated in the DHX9 overexpression group, whereas total protein expression did not change remarkably (Figure S3B). Conversely, DHX9 silencing downregulated the levels of p-mTOR, p-RPS6 and p-AKT (Figure S3C). These results suggested that DHX9 may activate the mTOR signaling pathway.

**Fig. 3:**
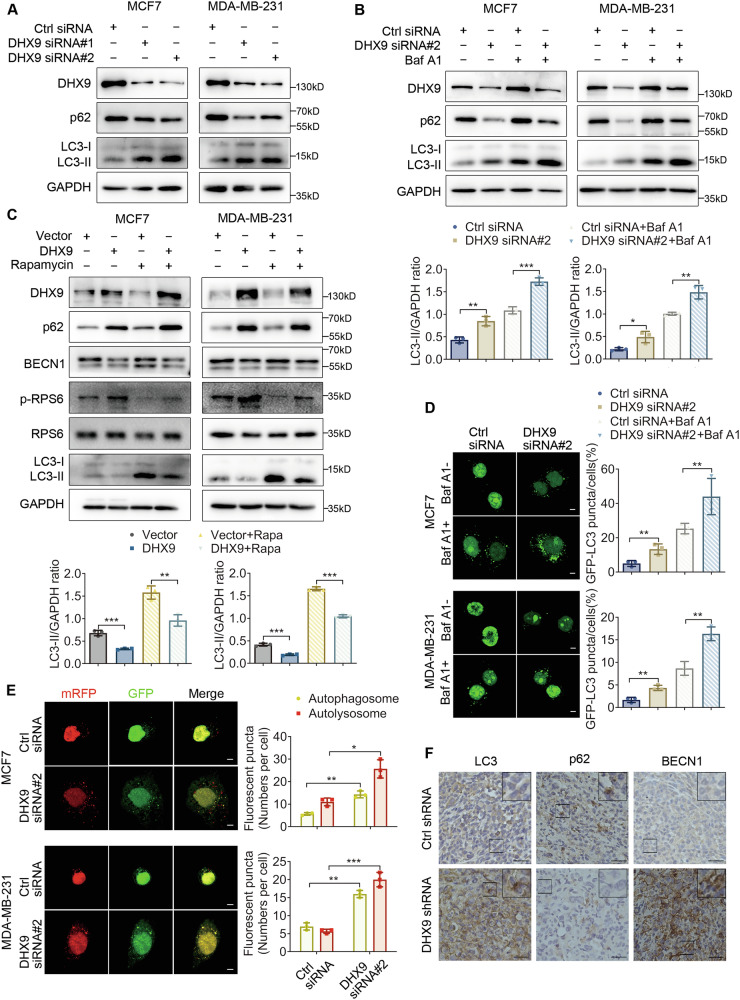
DHX9 knockdown induces autophagy. **A** Immunoblot to investigate the protein expressions of LC3-II and p62 following DHX9 knockdown. **B** BC cells with transiently decreased DHX9 or control cells were treated with 200 nM Baf A1 or DMSO for 4 hours and examined by Western blotting. Histograms (Below) showing the relative expression of LC3-II to GAPDH. **C** BC cells transiently overexpressing DHX9 or vector were treated with 10 nM Rapamycin or DMSO for 4 h. The protein levels of LC3-II, p62, BECN1, p-RPS6, RPS6 and DHX9 were detected by Western blotting. Histograms (Below) show the relative expression of LC3-II to GAPDH. **D** BC cells transiently expressed GFP-LC3 after DHX9 silencing. Twenty-four hours later, DMSO or 200 nM Baf A1 was applied to treat cells for 4 h. The numbers of GFP signal puncta per cell were observed with a confocal microscope and counted later. Scale bars, 5 μm. **E** BC cells transiently expressed mRFP-GFP-LC3 after DHX9 silencing. Twenty-four hours later, autophagosomes (yellow dots) and autolysosomes (red-only dots) per cell were observed and counted. Scale bars, 5 μm. **F** Representative IHC pictures showing the immunostaining of LC3, p62 and BECN1 in mouse xenografts (6 mice per group). Scale bars, 25 μm. Data are representative of three biological independent experiments (**A**–**E**) and are plotted as the mean ± SD (**B**–**E**). *P*-values were calculated by unpaired two-tailed Student’s t test (**B**–**E**). *p < 0.05, **p < 0.01, ***p < 0.001 vs. corresponding control.

Defective autophagy has been implicated in breast tumorigenesis. In most cell types, multiple steps throughout the autophagy process are adversely controlled by the mTOR pathway [15]. Thus, we assessed whether DHX9 participated in autophagy regulation by examining the protein levels of LC3-II and SQSTM1/p62, two well-established markers of autophagy. In BC cells, DHX9 knockdown led to decreased p62 and increased LC3-II expression (Fig 3A and Figure S3D). On the contrary, ectopic expression of DHX9 exhibited elevated p62 and reduced LC3-II levels (Figure S3E). To explore whether the increased LC3-II levels were primarily attributed to autophagy induction or impaired autophagosome-lysosomal fusion, LC3 turnover assays were performed. As indicated by increased levels of LC3-II with or without the lysosomal inhibitor bafilomycin A1 (Baf A1), knockdown of DHX9 promoted the formation of autophagosomes (Fig. 3B). Next, we explored whether DHX9 modulated autophagy via mTOR activity in BC cells. The results revealed that DHX9 knockdown or overexpression could still enhance or weaken autophagic flux in response to treatment with rapamycin, an mTOR inhibitor (Fig. 3C and Figure S3F).

Furthermore, we transfected the GFP-LC3 and mRFP-GFP-LC3 plasmids respectively into BC cells after DHX9 knockdown and observed the autophagosomes with a confocal microscope. The results indicated that DHX9 downregulation significantly increased the number of autophagic puncta in cells treated with either DMSO or Baf A1 (Fig. 3D). Meanwhile, DHX9 silencing significantly increased the quantity of yellow (autophagosomes) and red-only (autolysosomes) speckles (Fig. 3E), indicative of enhanced autophagic flux. Additionally, histological analysis in the tumor xenograft tissue sections showed elevated levels of LC3, BECN1 and reduced p62 levels in the DHX9- knockdown group (Fig. 3F). In summary, our findings implied that reduced DHX9 might be essential for increased autophagosome formation and autophagy.

### The oncogenic effect of DHX9 on BC cells depends on BECN1

**Fig. 4:**
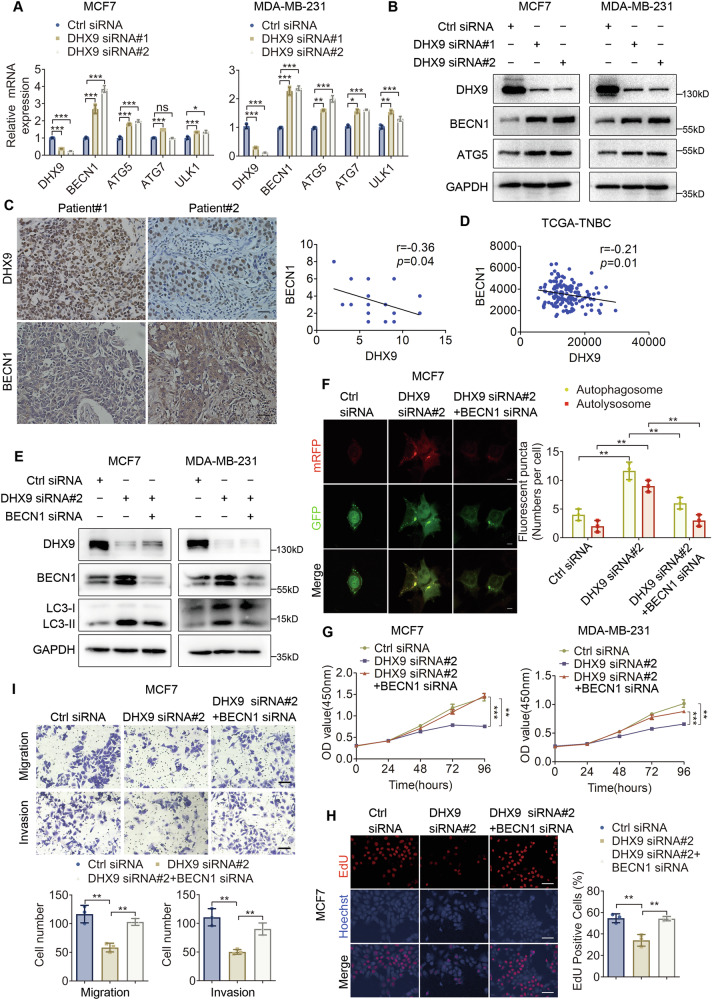
BECN1 knockdown reverses the biological effects of DHX9 silencing on BC cells. **A** Quantitative RT-PCR to inspect the mRNA expression of several ATGs after DHX9 knockdown. **B** Immunoblot to investigate the expression of BECN1, ATG5 and DHX9 after DHX9 knockdown. **C** Representative IHC photographs of BECN1 and DHX9 staining in primary BC tissues of the tissue microarray from two patients were shown (*n* = 31). Scale bars, 40 μm. The scatter plot (Right) shows the correlation analysis between DHX9 and BECN1. Pearson’s correlation analysis was conducted to assess the linear relationship between variables. *P* values (two-tailed) were calculated by Pearson r. **D** The correlation between DHX9 and BECN1 expression in TCGA-TNBC dataset. **E** Ctrl siRNA, DHX9 siRNA alone or DHX9 siRNA combined with BECN1 siRNA for BC cells treatment respectively. Then the protein levels of DHX9, BECN1 and LC3-II were analyzed by Western blotting. **F** MCF7 cell was transfected with mRFP-GFP-LC3 after DHX9 silencing or combined silencing of DHX9 and BECN1. Twenty-four hours later, autophagosomes (yellow dots) and autolysosomes (red-only dots) per cell were observed and counted. Scale bars, 5 μm. **G**, **H** Cell viability (**G**) and EdU-positive rates (**H**) were evaluated after DHX9 downregulation or combined downregulation of DHX9 and BECN1 in BC cells. Scale bars, 50 μm. **I** Cell invasion and migration ability were evaluated after DHX9 downregulation or combined downregulation of DHX9 and BECN1 in BC cells. Scale bars, 50 μm. Data are representative of three biological independent experiments (**A**, **B**, **E**–**I**) and are plotted as the mean ± SD (**A**, **F**–**I**). *P* values were calculated by unpaired two-tailed Student’s t test (**A, F–I**). *p < 0.05, **p < 0.01, ***p < 0.001 vs. corresponding control. ns not significant.

Autophagy is regulated not only by kinase signals but also by ATGs [16]. To investigate the underlying mechanism of DHX9-mediated autophagy impairment, we examined the expression of four critical ATGs (*ATG5*, *ATG7*, *BECN1* and *ULK1)* required for autophagosome formation. As revealed in Fig. 4A, although the mRNA expressions of all these genes were markedly increased with DHX9 suppression, BECN1 exhibited the most obvious upward trend. Subsequently, as revealed in Fig. 4B and Figure S4A, DHX9 knockdown elevated BECN1 and ATG5 protein levels, while its ectopic expression reduced them (Figure S4B).

Compared with normal samples, tumor samples have lower BECN1 expression levels, which has been reported to contribute to cancer pathogenesis and progression [3, 4]. Lower BECN1 expression is associated with poorer prognosis in HER2+ and basal-like BC [17]. Thus, we wondered whether DHX9 could promote the malignant phenotypes of BC cells and inhibit autophagy via suppressing the expression of BECN1. Above all, IHC staining and TCGA data analysis both revealed an adverse correlation between DHX9 and BECN1 (Fig. 4C, D). As indicated in Fig. 4E and Figure S4C, the increase in the protein levels of LC3-II caused by DHX9 knockdown was partially weakened when BECN1 was silenced in MCF7 and MDA-MB-231 cells. Accordingly, immunofluorescence assays displayed that enhanced autophagic flux caused by DHX9 downregulation could be blocked by BECN1 suppression (Fig. 4F and Figure S4D). In addition, the rescue experiments proved that BECN1 depletion largely reversed the detrimental effects of DHX9 deficiency on cell viability (Fig. 4G), colony number (Figure S4E), EdU-positive rates (Fig. 4H and Figure S4F), as well as cell invasion and migration ability (Fig. 4I and Figure S4G). Interestingly, similar to BECN1 knockdown, ATG5 silencing also reversed the autophagy-promoting and tumor-suppressive effects of DHX9 silencing (Figure S4H–J). However, in cells overexpressing DHX9, where BECN1 expression was significantly decreased, upregulation of ATG5 failed to reverse the autophagy-inhibition and tumor-promoting effects induced by DHX9 overexpression (Figure S4K–M), which contrasted with the marked reversal effects observed upon BECN1 upregulation in the same cellular context (Figure S4N–P). This suggests that while ATG5, as one of the core components of the autophagy-initiating machinery, may be required for BECN1-mediated tumor-suppressive effects of DHX9 silencing, only BECN1 functions as the essential triggering factor.

Therefore, these results indicated that DHX9 could inhibit autophagy and exerted its oncogenic effects at least partly through the repression of BECN1.

### DHX9 occupies the promoter of BECN1 and represses its transcription

**Fig. 5:**
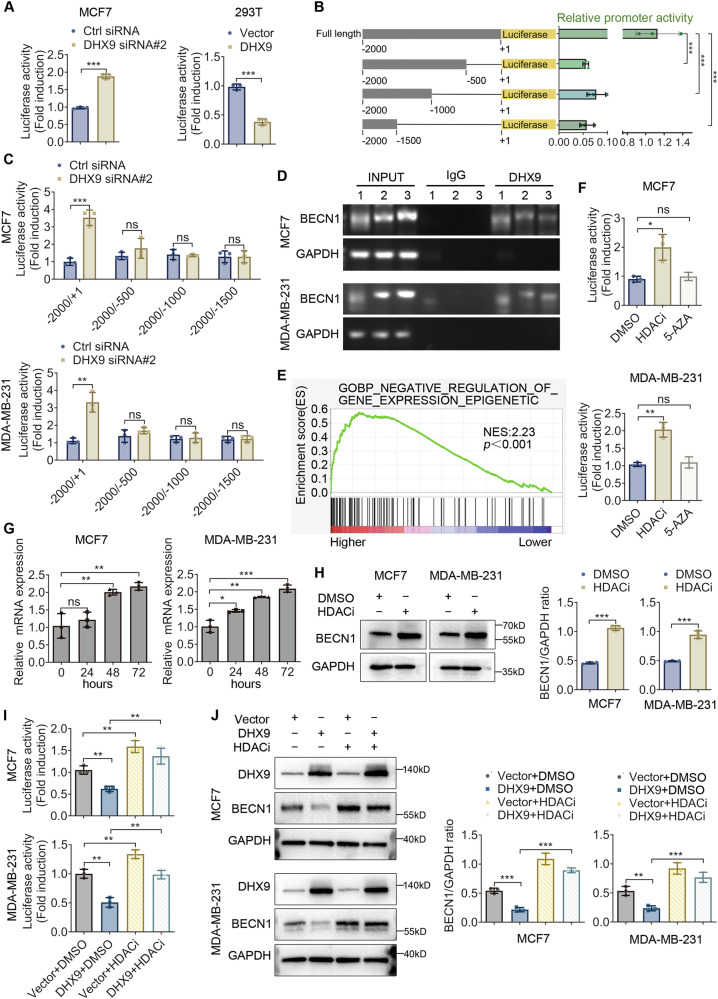
DHX9 occupies the promoter of BECN1 and represses its transcription. **A** Cells were transfected with BECN1-Luc after DHX9 silencing (MCF7) or overexpression (293T). Twenty-four hours later, the collected cells were subjected to the luciferase activity assay. **B** BC cells underwent co-transfection of pGL3-basic vectors harboring different fragments of the BECN1 promoter and pRL-TK control plasmid. The luciferase activity levels relative to the Renilla luciferase activity levels and the levels of the -2000 to +1 fragment were calculated. **C** pGL3-basic vectors harboring different fragments of the BECN1 promoter were transfected into BC cells, co-transfected with pRL-TK plasmid following DHX9 knockdown. The luciferase activity levels relative to the Renilla luciferase activity levels and the levels of the control group were calculated. **D** ChIP-PCR was performed with the indicated antibodies against DHX9 at the BECN1 promoter in BC cells. 1, 2, 3 indicated three different pairs of primers located in the −500 to +1 bp region of the BECN1 promoter. **E** Gene Ontology analysis using the TCGA-BRCA dataset shows that DHX9 high expression was enriched in the “Negative_Regulation_of_Gene Expression_Epigenetic” pathway. **F** Dual luciferase reporter assay to detect the transcriptional suppressive effect on BECN1 promoter with treatment of HDACi or 5-Aza-CdR (5-Aza). **G** The mRNA expression of BECN1 was detected by qPCR following treatment with HDAC inhibitor in indicated times. **H** Immunoblot to investigate the protein level of BECN1 following HDACi treatment. Histograms (Right) show the relative expression of BECN1 to GAPDH. **I**, **J** Dual luciferase reporter assay (**I**) and Western blot analysis (**J**) to assess whether the repression of BECN1 by DHX9 depends on HDAC activity. Histograms (**J**, Right) shows the relative protein expression of BECN1 to GAPDH. Data are representative of three biological independent experiments (**A−D**, **F−J**) and are plotted as the mean ± SD (**A−C**, **F−J**). *P* values were calculated by unpaired two-tailed Student’s t test (**A**, **C**, **F−J**) and Dunnett’ s multiple comparisons test (**B**). *p < 0.05, **p < 0.01, ***p < 0.001 vs. corresponding control. ns, not significant.

As a crucial transcription cofactor, DHX9 is known to interact with several transcription regulatory factors [12, 18, 19]. We speculate that it may repress the transcription of BECN1. As expected, the luciferase activity of BECN1 promoter was significantly enhanced upon DHX9 knockdown in MCF7 cells (Fig. 5A). Conversely, DHX9 overexpression repressed the promoter activity in 293T cells (Fig. 5A). To further confirm the potential binding region of BECN1 by which DHX9 affected promoter activity, different lengths of promoter fragments were cloned and inserted into luciferase reporter plasmids. As illustrated in Fig. 5B, the promoter fragments with −500 to +1 deleted showed much lower activity than that with the full-length promoter. Following DHX9 knockdown, only the activity of the full-length promoter containing the -500 to +1 fragment exhibited a significant increase (Fig. 5C), whereas the activity of the other fragments remained unchanged. These results implied that the promoter region containing −500 to +1 might be important for DHX9 to modulate the transcriptional activity of BECN1.

Furthermore, ChIP-PCR was conducted to assess the occupation of DHX9 in the promoter region of BECN1. The results indicated that DHX9 was recruited to the proximal promoter of BECN1 in BC cells (Fig. 5D). Collectively, these findings implied that DHX9 could directly bind to the BECN1 promoter region, which may play a pivotal role in repressing its transcription.

### DHX9-mediated BECN1 repression requires HDAC activity, but not DNMT activity

Epigenetic modifications including histone deacetylation and DNA methylation are common mechanisms leading to transcription repression [20, 21]. DNA methylation is mediated by DNA methyltransferases (DNMTs), while histone deacetylation by histone acetyltransferases (HATs) and histone deacetylases (HDACs). Gene Ontology (GO) analysis of the TCGA-BRCA data showed that high DHX9 expression was enriched in the “Negative_Regulation_of_Gene Expression_Epigenetic” pathway (Fig. 5E), suggesting that DHX9 may be implicated in epigenetic regulation. Interestingly, several helicase family members repressed transcription by interacting with HDACs [22]. In addition, we found that the main epigenetic modifications on the promoter of BECN1 were H3K27ac and H3K9ac in BC cells through analysis of ChIP-seq data from TFmapper (Figure S5A, B) and UALCAN (Figure S5C).

To further clarify the potential epigenetic mechanism for DHX9-mediated transcriptional repression of BECN1, we identified whether histone deacetylation or DNA methylation is responsible for its transcriptional activity. The results revealed that HDAC inhibitor (HDACi) but not DNMT inhibitor 5-Aza-CdR could significantly increase the transcriptional activity of BECN1 (Fig. 5F). Then, we observed that the BECN1 mRNA levels in BC cells treated with HDACi gradually increased in a time-dependent manner (Fig. 5G). Meanwhile, BECN1 protein levels were upregulated following HDACi treatment (Fig. 5H), and HDACi could reverse DHX9-induced BECN1 inhibition in BC cells (Fig. 5I, J). In addition, HDACi treatment did not alter the endogenous DHX9 expression level (Fig. 5J). Collectively, these findings hinted that DHX9-mediated BECN1 repression could be attributed to histone deacetylation.

### DHX9 interacts with HDAC5 and synergistically represses the transcription of BECN1

**Fig. 6:**
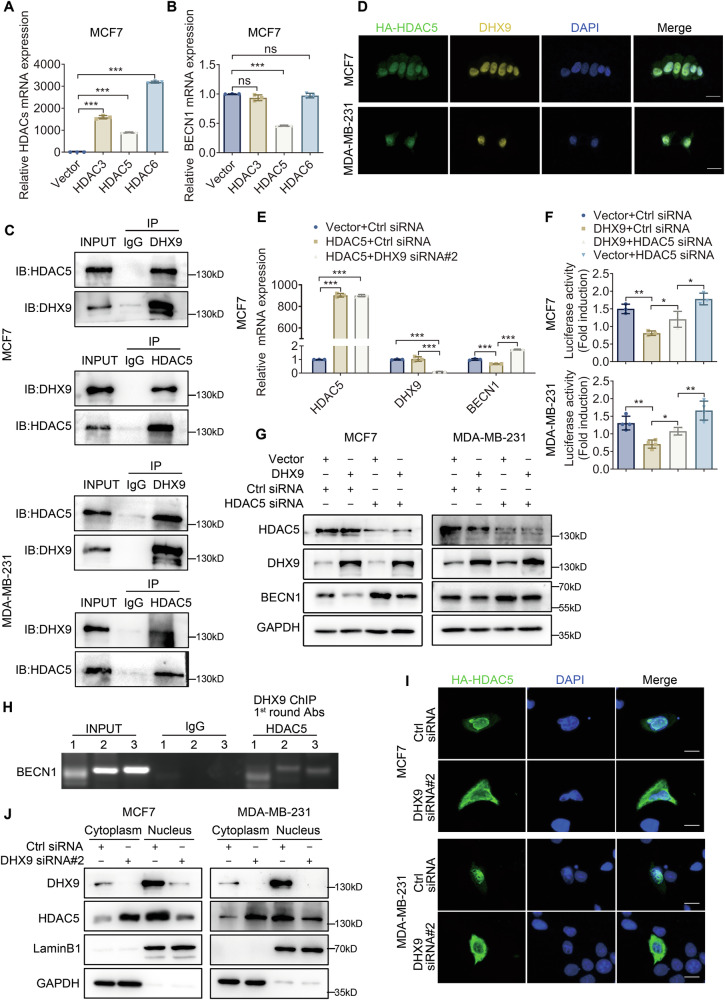
DHX9 interacts with HDAC5 and synergistically represses the transcription of BECN1. **A** Quantitative RT-PCR to verify the overexpression efficiency of HDAC3, 5, 6 in MCF7. **B** Quantitative RT-PCR to inspect the mRNA expression of BECN1 after HDAC3, HDAC5, or HDAC6 overexpression respectively in MCF7. **C** BC cells were collected to analyze the physical interaction between HDAC5 and DHX9 by IP applying the indicated antibodies against HDAC5 and DHX9. **D** Immunofluorescence analyses were executed to investigate the subcellular localization of DHX9 and HA-HDAC5 by fluorescence microscopy. Scale bars, 20 μm. **E** MCF7 cell overexpressing Vector or HDAC5 were co-transfected with Ctrl or DHX9 siRNA for 48 h and then subjected to qRT-PCR assays. **F** BC cells overexpressing Vector or DHX9 were co-transfected with Ctrl or HDAC5 siRNA for 24 h and then subjected to a dual luciferase reporter assay. **G** BC cells overexpressing Vector or DHX9 were transfected with Ctrl or HDAC5 siRNA for 48 h, then the indicated proteins were analyzed by Western blotting. **H** Re-ChIP showing the colocalization of DHX9 with HDAC5 at the BECN1 promoter. 1, 2, 3 indicated three different pairs of primers located in the -500 to +1 bp region of the BECN1 promoter. **I**, **J** Immunofluorescence analysis (**I**) and nuclear-cytoplasmic fractionation experiment (**J**) were used to evaluate the nucleoplasm distribution of HDAC5 after DHX9 silencing. Scale bars, 10 μm. Data are representative of three biological independent experiments (**A**–**J**) and are plotted as the mean ± SD (**A**, **B**, **E**, **F**). *P* values were calculated by unpaired two-tailed Student’s t test (**A, B, E, F**). *p < 0.05, **p < 0.01, ***p < 0.001 vs. corresponding control. ns, not significant.

To gain deeper insight into the mechanism, we attempted to determine the interactors of DHX9. Utilizing Biogrid database, we identified two potential HDAC family members (HDAC5 and HDAC6) that may interact with DHX9 (Figure S6A). Additionally, KLF5 is reported to cooperate with HDAC3 to suppress BECN1 transcription [23]. Thus, we focused on HDAC3, HDAC5 and HDAC6 to elucidate a new potential interaction between HDACs and DHX9. DHX9 bound to HDAC3, HDAC5 and HDAC6 when each HDAC was tagged with HA and co-transfected into 293T cells with flag-tagged DHX9 (Figure S6B). The overexpression efficiency of HDACs was confirmed using qRT-PCR (Fig. 6A and Figure S6C). Then we desired to determine which HDAC regulates BECN1 expression in BC cells. Surprisingly, only HDAC5, but not HDAC3 or HDAC6 exhibited a significant inhibition on the mRNA expression of BECN1 (Fig. 6B and Figure S6D). Moreover, HDAC5 physically interacted with DHX9 (Fig. 6C). In the HA empty vector control group, the green signal (HA staining) exhibited a uniform distribution throughout the cytoplasm and the nucleus (Figure S6E), whereas in the HA-HDAC5 overexpression group, the HA signal showed predominant nuclear localization similar to the yellow signal (Fig. 6D). These results showed a nuclear colocalization of HDAC5 with DHX9.

Next, we investigated whether DHX9-mediated BECN1 silencing depended on HDAC5. As shown in Fig. 6E and Figure S6F, enhanced HDAC5 expression inhibited BECN1 mRNA levels, which was further restored after DHX9 knockdown. Western blotting also exhibited similar trends at the protein levels (Figure S6G). Additionally, the suppressive effect of DHX9 on BECN1 could be rescued by HDAC5 knockdown (Fig. 6F, G and Figure S6H). To further verify the colocalization of DHX9 with HDAC5 on the BECN1 promoter, ChIP-Re-ChIP assays were conducted. The results indicated that DHX9 and HDAC5 could directly bind to the promoter region of the *BECN1* gene (Fig. 6H).

Furthermore, we observed that DHX9 overexpression had no significant effect on the protein levels of HDAC5 (Fig. 6G and Figure S6G). Since HDAC5 shuttles between the nucleus and the cytoplasm under certain circumstances, we speculated that DHX9 may regulate its nucleocytoplasmic transport. Expectedly, in HDAC5-overexpressing cells, transient DHX9 knockdown reduced the nucleocytoplasmic HDAC5 ratio (Fig. 6I). Similarly, nuclear-cytoplasmic fractionation revealed that DHX9 knockdown decreased nuclear HDAC5 and increased cytoplasmic HDAC5 (Fig. 6J). To sum up, these findings validated the hypothesis that through interaction with HDAC5, DHX9 facilitated its nuclear localization, thereby synergistically repressing BECN1 transcription.

### DHX9 represses BECN1 transcription via histone deacetylation

**Fig. 7:**
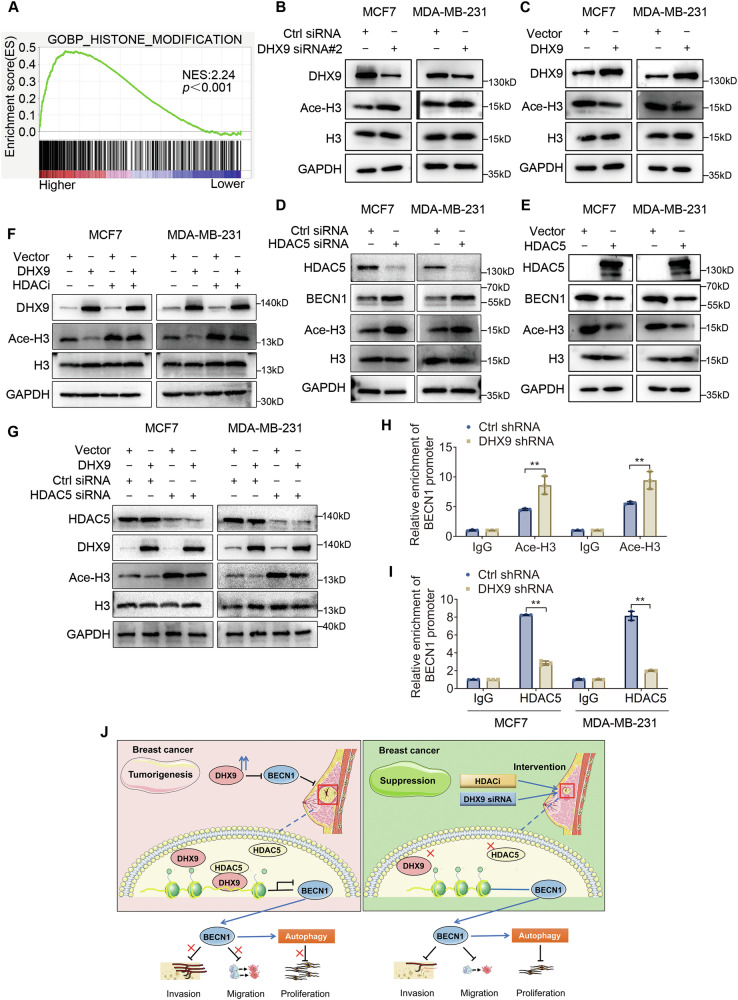
DHX9 represses BECN1 transcription via histone deacetylation. **A** Gene Ontology-Biological Process analysis indicated that gene sets related to histone modification were enriched in the DHX9 high expression group. **B**, **C** Immunoblot analysis revealed the impact of DHX9 silencing (**B**) or augmentation (**C**) on the acetylation level of histone H3. **D**, **E** Immunoblot analysis revealed the effect of HDAC5 silencing (**D**) or augmentation (**E**) on the acetylation level of histone H3. **F**, **G** Immunoblot analysis revealed that DHX9-mediated deacetylation of histone H3 depended on HDAC activity (**F**) or HDAC5 (**G**). **H**, **I** ChIP-qPCR showed the enrichment levels of Ace-H3 or HDAC5 on BECN1 promoter after DHX9 downregulation. **J** A sketch map to elucidate the biological function of DHX9 in BC. Enhanced expression of DHX9 represses BECN1 transcription by recruiting HDAC5 to its promoter mediating the deacetylation of histone H3 and contributes to impaired autophagy and tumor development in BC. Data are representative of three biological independent experiments (**B**–**I**) and are plotted as the mean ± SD (**H**, **I**). *P* values were calculated by unpaired two-tailed Student’s t test (**H**, **I**). **p < 0.01 vs. corresponding control.

HDACs play pivotal roles in regulating transcription and are linked to a range of diseases, including cancer. Based on our findings and GO analysis suggesting the role of DHX9 in histone modification (Fig. 7A), we inferred that the negative regulation of BECN1 by DHX9 likely depended on histone deacetylation. As mentioned previously, the top-rated epigenetic modification in BECN1 promoter is the deacetylation of histone H3 in BC cells (Figure S5B, C). Subsequent investigations revealed that DHX9 knockdown increased the acetylated histone H3 (Ace-H3) levels (Fig. 7B and Figure S7A), whereas DHX9 overexpression reduced them (Fig. 7C and Figure S7B). Similarly, HDAC5 deficiency or overexpression showed the same effect on Ace-H3 as DHX9 (Fig. 7D, E and Figure S7C, D). Furthermore, we investigated whether DHX9-mediated histone deacetylation relied on HDAC5. As illustrated in Fig. 7F, G and Figure S7E, F, the reduction in Ace-H3 levels mediated by DHX9 overexpression was reversed after HDACi treatment or HDAC5 knockdown.

Additionally, ChIP assays were performed to explore how DHX9 repressed BECN1 transcription. As shown in Fig. 7H, I, DHX9 knockdown attenuated HDAC5 recruitment to the BECN1 promoter and enhanced histone H3 acetylation in the BECN1 promoter region. These results indicated that DHX9 may recruit HDAC5 to the BECN1 promoter, mediating the deacetylation of H3 and ultimately inhibit BECN1 transcription (Fig. 7J).

### DHX9 silencing improves the respondence of BC cells to chloroquine

Given the accumulating evidence supporting the autophagy inhibitor-chloroquine (CQ) in tumor suppression and cancer therapy [24], and the preliminary validation of DHX9 inhibitor exerting anti-tumor effects [25, 26], we wondered the combination effects of DHX9 silencing and CQ treatment. As shown in Figure S7G, CQ further enhanced LC3-II accumulation in DHX9-silenced cells. CCK-8 assays, colony formation analyses, and EdU incorporation assays collectively demonstrated that while CQ inhibited BC cells proliferation under basal conditions, this suppressive effect was significantly enhanced in DHX9-silenced cells (Figure S7H–J). Similarly, combined DHX9 silencing and CQ treatment exerted stronger inhibitory effects on cell migration and invasion compared to monotherapy of CQ or DHX9 silencing (Figure S7K). Moreover, MDA-MB-231 cells seems more sensitive to CQ treatment than MCF7, consistent with previous report [27]. These findings suggest that DHX9 inhibition may serve as a chemosensitizer to enhance CQ-mediated anti-tumor efficacy.

## Discussion

Until now, the functions of DExD/H-box helicases in autophagy regulation have been discovered [11]. Notably, they can either positively or negatively modulate autophagy under different conditions. For instance, DDX53 promotes autophagy by binding to the ATG5 promoter to activate its transcription [28]. DDX17 upregulation inhibits autophagy by miRNA-mediated BECN1 repression [29]. Although recent studies have described the impact of DHX9 on several pathways such as the AKT, NF-κB and TP53 pathways [12, 14, 30], whether it modulates autophagy remains elusive. Here, both GSEA analysis and further experimental investigations disclosed that DHX9 was positively related to the mTOR signaling pathway. Interestingly, we also found DHX9 silencing increased autophagic activity by promoting autophagosome formation. Furthermore, DHX9 diminished LC3-II levels even with rapamycin, implying its autophagy-inhibition effect was not entirely mTOR-dependent.

The complex association between autophagy and BC has been demonstrated in multiple studies. This is the first publication displaying that DHX9 exerts its pro-carcinogenic effects by suppressing autophagy, which may unveil a previously unrecognized function of DHX9 in autophagy. Given the extensively characterized transcriptional regulatory role of DHX9 and its nuclear localization in BC tissues, we investigated the expression of several key ATGs following DHX9 knockdown or overexpression in BC cells. Among them, *BECN1* was identified as the target gene with the most significant fold change. Intriguingly, BECN1 can not only inhibit BC cell proliferation via autophagy [31], but also suppress cell invasion and migration in an autophagy-independent manner [32]. Additionally, some researches have pointed out that BECN1 is negatively correlated with oncogenic pathways [33]. Importantly, our study identified DHX9 as a negative regulator of BECN1 transcription. Rescue assays displayed that BECN1 knockdown reversed the increased autophagy and reduced cell proliferation, invasion and migration caused by DHX9 depletion.

Previous studies show that DHX9 acts as a bridging factor that binds to transcription factors, transmitting signals to RNA polymerase II [18]. It also binds to specific sequence on the promoter of some genes [34]. Despite the majority of research documents its involvement in transcription activation, few studies focus its role in transcriptional inhibition. Actually, RNA helicases also function as transcriptional repressors. For instance, DDX5 binds to p53 at the promoter of the *Pkd1* gene to restrain its transcription [35]. Furthermore, both DDX20 and DDX5 can collaborate with certain HDACs to repress transcription [36, 37]. Notably, DHX9 binds to the DNA-binding domain of *TonEBP* and inhibits its promoter activity [38], suggesting a negative role in transcription regulation. Indeed, bioinformatics analysis shows that DHX9 is associated with histone modification and negatively regulating transcription epigenetically. Thus, we wondered whether DHX9 could inhibit BECN1 transcription in these ways. Our findings highlighted a new function of DHX9 in repressing BECN1 transcription through histone deacetylation at its promoter, which was reversed by the HDAC inhibitor, but not the DNMT inhibitor 5-Aza-CdR. Therefore, determining how DHX9 contributes to histone deacetylation has become an urgent task.

HDACs remove the acetylation of lysine residues in histone and nonhistone proteins. Multiple cellular processes, such as gene expression and protein stability [39], and various diseases including cancers, are regulated by HDACs. HDACs interact with DNA binding factors to draw themselves to DNA hypersensitive sites and gene promoters. Of note, their activities are also observed in intergenic areas devoid of DNA binding factors, suggesting their recruitment to those areas may be attributed to alternative, yet undefined mechanisms [40]. Retinoblastoma (RB) promotes histone deacetylation and gene silencing via recruiting HDAC5 or HDAC1 under different circumstances [41, 42]. Previous study reported that in prostate cancer cells, KLF5 could collaborate with HDAC3 to repress BECN1 transcription [23]. Here in our study, DHX9 may recruit HDAC5 to the BECN1 promoter, leading to histone H3 deacetylation and BECN1 transcriptional repression.

Another surprising finding is that DHX9 silencing could change the nucleoplasm distribution of HDAC5. Controlled nucleocytoplasmic distribution is a major mechanism regulating class II HDACs [43]. Like HDAC4, HDAC5 translocates from the nucleus to the cytoplasm upon phosphorylation [44], while its interaction with the transcription factor MEF2 induces the nuclear localization [45]. Actually, HDAC5 is predominantly located in the nucleus in MCF7 and MDA-MB-231 cells [46]. Our study highlights that DHX9, as a transcriptional cofactor, may be implicated in the subcellular localization of HDAC5 in BC. Thus, further studies are needed to fully comprehend the underlying mechanism that explains how DHX9 affects HDAC5 nuclear translocation.

## Conclusions

To sum up, DHX9 is upregulated in BC cells and tissues, and its elevated expression is associated with an adverse prognosis. Functional analyses indicate that DHX9 exacerbates BC proliferation, migration, invasion and impedes the autophagic flux. Mechanistically, DHX9 recruits HDAC5 to the promoter region of BECN1, mediating the deacetylation of histone H3 and ultimately inhibits BECN1 transcription. Together, the present work identifies a previously unrecognized role of DHX9 in epigenetically silencing BECN1.

This highlights the potential of DHX9 as a biomarker and a therapeutic target in BC, while suggests the clinical applicability of HDACi in BC treatment. Several pan-HDACi, including Vorinostat and Belinostat, have been approved by the US Food and Drug Administration (FDA) for clinical applications of lymphoma. Recent clinical trials have shown that Entinostat-based combination regimen improves survival probability in both HR+ and TNBC subtypes [47, 48]. A study showed that LMK235, a selective inhibitor for HDAC4/5, demonstrated enhanced efficacy in triple-negative BC cells (MDA-MB-231) compared to luminal subtypes (MCF-7) [49]. Furthermore, studies have demonstrated that DHX9 suppression exhibits lethality in most tumor cell types while causing no obvious detrimental effects on normal cells and organs, highlighting the feasibility of DHX9 as a therapeutic target [50]. ATX968, a newly developed small-molecule inhibitor of DHX9, exhibits tumor growth inhibition in cancers with microsatellite instability and mismatch repair deficiency. Its role in other tumor types such as BC needs more investigation [25]. Interestingly, although CQ functions as an autophagy inhibitor, which is opposed to DHX9 silencing-induced autophagy activation, our study showed the enhanced tumor-inhibitory effects upon their combined application. This phenomenon may be attributed to the anti-tumor mechanism (e.g. apoptosis and necroptosis) of CQ unrelated to autophagy, as it has been clarified that CQ sensitizes BC cells to chemotherapy independent of autophagy [24, 51]. And the tumor-suppressive effects of DHX9 silencing mediated by BECN1 is also not entirely autophagy-dependent. Additionally, several studies have reported that combination of CQ with autophagy-promoting targeted agents may enhance anti-tumor effects through mutually compensatory mechanisms [52]. These results indicate that targeting DHX9 could enhance the sensitivity of BC cells to CQ. Altogether, our study showed the tumor inhibition effect of DHX9-HDAC5-BECN1 axis in ER+ and TNBC cell lines, implying the therapeutic potential of DHX9 or HDAC5 inhibitors in at least HR+ or TNBC subtypes. However, further mechanistic and preclinical validation is warranted.

## Materials and Methods

### Cell lines

MCF7, T47D, MDA-MB-231, MDA-MB-468 and 293T were acquired from the Cell Bank of the Chinese Academy of Sciences (Shanghai, China). The normal human breast epithelial cell line MCF10A obtained from laboratory preservation was cultured in DMEM/F12 (Gibco^TM^, ThermoFisher Scientific, Massachusetts, USA) supplemented with 10% fetal bovine serum (Gibco) and 1% penicillin-streptomycin (Gibco). All the other cell lines were grown in high glucose DMEM (Gibco). Every cell line was kept under regular conditions with 5% CO_2_ at 37 °C. All cell lines used in this study were authenticated by short tandem repeat (STR) profiling and tested negative for mycoplasma contamination.

### Clinical samples

Human breast cancer and paired noncancerous tissues were collected at the Breast Surgery Department of Nanfang Hospital from 2021 to 2022. The Medical Ethics Committee of Nanfang Hospital authorized this study. The Declaration of Helsinki was scrupulously followed. A commercialized breast cancer tissue microarray containing 45 pairs of breast cancer tissues and para-carcinoma tissues was purchased from OUTDO Biotechnology (Shanghai, China). The TCGA-BRCA expression profile and clinical data were obtained from the UCSC Xena database [53]. The expression profile data of the target genes were downloaded from the GEO database include GSE162228 [54] and GSE22820 [55]. GSE162228 included 109 breast cancer patient samples and 24 matched adjacent normal breast tissue samples. GSE22820 included 176 primary breast cancer patient samples and 10 normal breast tissue samples.

### Plasmids and reagents

The promoter regions of BECN1 were inserted into the pGL3-basic vector (Promega, Wisconsin, USA). Tsingke Biotechnology (Beijing, China) provided the pLKO.1-GFP-shDHX9 plasmid for purchase. The expression plasmid pcDNA3.3-3×Flag-DHX9 was purchased from Biogene (Shanghai, China). The pcDNA3.1-3×HA-HDAC3, pcDNA3.1-3×HA-HDAC5, pcDNA3.1-3×HA-HDAC6, pcDNA3.1-3×flag-BECN1 and pcDNA3.1-3×flag-ATG5 plasmids were constructed in our laboratory through PCR amplification. The primers involved in PCR amplification are shown in the Supplementary Materials 3. Verification of the newly constructed plasmids was conducted by DNA sequencing. Bafilomycin A1, Rapamycin and 5-Aza-CdR were sourced from Selleck (Texas, USA). HDAC inhibitor was from Biyuntian (Shanghai, China).

### Cell proliferation assays

In 96-well plates, cells (3 × 10^3^ cells/well) were seeded in triplicate for the cell counting kit-8 (CCK8) assay. After 0, 24, 48, 72 and 96 h, each well received 10 μL CCK-8 (Apexbio, Texas, USA) mixed with 90 μL DMEM followed by an incubation period of 1 h. Then the optical density 450 value was measured with a microplate reader (Tecan, Switzerland).

BC cells (1 × 10^3^ cells/well) were placed in 6-well plates for the colony-formation assay. After 2 weeks of growth in a regular incubator, colonies were fixed, stained and counted.

Following the guidance published by the vendor (Biyuntian), 5-ethynyl-2′-deoxyuridine (EdU) assay was carried out. In brief, the cells in 24-well plates were treated with 10 μM EdU for 2 hours. Subsequently, after being fixed, washed and permeabilized, the cells were subjected to EdU detection for 30 minutes, followed by Hoechst 33342 staining for an additional 10 min. The ratio of EdU-positive cells to all Hoechst-positive cells was calculated to get the EdU incorporation rate.

### Transwell invasion and migration assays

Support membranes with 8 μm pores (BD, New Jersey, USA) were placed in 24-well plates for migration and invasion tests. The upper chamber with (invasion) or without (migration) Matrigel (BD) coated in advance was filled with 200 μL cell suspension (3 × 10^5^ cells/mL) in serum-free medium, while the lower chamber received 600 μL culture medium containing 20% FBS. Then the inserts were taken out from the wells after incubation for 24 h. Following fixation and staining, the cells on the lower surface were examined using a microscope. Five randomly selected optical microscope fields were photographed and observed.

### RNA interference

Following the supplier′s instructions, siRNA oligos of 50 nM synthesized by GenePharma (Shanghai, China) were transfected into cells with Lipofectamine 2000 reagent (Invitrogen^TM^, ThermoFisher Scientific, Massachusetts, USA). The sequences for the indicated siRNAs are shown in the Supplementary Materials 3.

### RNA extraction and quantitative RT-PCR

The cells were treated with RNAiso Plus (AG, Changsha, China) to isolate total RNA. Then 2 μg RNA was reverse-transcribed using the cDNA Synthesis SuperMix kit (Yeasen, Shanghai, China). Gene expression was detected using SYBR method (Yeasen) and a QuantStudio3 instrument (ThermoFisher Scientific). The primers involved in real-time PCR are shown in the Supplementary Materials 3.

### Western blot

After the cells were lysed with RIPA lysis buffer (Biyuntian) supplemented with protease inhibitors (Biyuntian) and centrifuged at 12,000 rcf for 15 min at 4 °C, protein loading buffer (Biyuntian) was added to the supernatant for denaturation. The denatured samples were loaded into the wells of SDS-PAGE gels for electrophoresis. Once the electrophoresis was complete, the membrane transfer was proceeded. Then the nonspecific binding sites were blocked with 5% skim milk for 2 h. After incubation overnight at 4 °C with the indicted primary antibodies, the blots were probed for 2 h at ambient temperature with secondary antibodies conjugated with horseradish peroxidase (Proteintech, Wuhan, China). Photographs were taken with a chemiluminescence apparatus (Tennon5200, Shanghai, China). The detailed information of the antibodies is listed in Supplementary Materials 3.

### Nuclear-cytoplasmic separation assay

The manufacturer’s instructions (ThermoFisher Scientific) were followed to perform this assay. Briefly, the cytoplasmic and nuclear components were extracted sequentially. The samples were denatured and set aside on ice for use.

### Co-immunoprecipitation (Co-IP)

Briefly, the cell extracts underwent an overnight incubation at 4°C combined with antibody-coupled protein A/G magnetic beads (Selleck, Texas, USA). Following elution and denaturation, the immunoprecipitated proteins were collected for Western blot analysis. All antibody information is listed in the Supplementary Materials 3.

### Dual luciferase reporter assay

In brief, promoter-luciferase plasmids of 400 ng and internal control plasmid pRL-TK of 40 ng were co-transfected into cells placed in 24-well plates. Twenty-four hours later, the luciferase activity, normalized to Renilla luciferase levels, was measured using a Dual Luciferase Assay kit (Vazyme, Nanjing, China).

### Chromatin immunoprecipitation (ChIP) and Re-ChIP

The ChIP procedure was performed with the description of ChIP Kit (Biyuntian). Briefly, cells (1 × 10^7^) were cross-linked with 37% formaldehyde (Macklin, Shanghai, China), and the resulting cell lysate collected after centrifugation was resuspended in ChIP buffer. Following sonication in ice, the chromatin underwent two incubation periods overnight at 4 °C, first with 2.5 μg indicated antibodies and then with the specific protein A/G magnetic beads. After a series of washes, the DNA-protein complexes were separated. TE buffer was employed to suspend the purified DNA for PCR. The primers for promoters are shown in the Supplementary Materials 3.

The re-ChIP assay was carried out according to previously described methods [56]. Generally speaking, the elute complexes of each sample were eluted with 100 μL of 10 mM Dithiothreitol (Solarbio, Beijing, China) from the beads in the primary ChIPs for 30 min at 37 °C. Following centrifugation, the supernatant underwent an additional ChIP treatment after diluting 20 times with ChIP buffer.

### Immunofluorescence

The cells were pre-seeded on cell culture slides (NEST, Wuxi, China). After fixed with methanol and blocked in 2% BSA, the cells were incubated overnight at 4 °C with primary antibodies. The following day, fluorophore-conjugated secondary antibodies (details in Supplementary Materials 3) were prepared to treat cells for 1 h at ambient temperature. Then 4′,6-diamidino-2-phenylindole (DAPI) was applied to mark the nuclei. After the slides were washed and mounted, images were photographed using fluorescent microscope or confocal microscope. For autophagosome observation, nuclei were labelled with DAPI after the cells were fixed and then a confocal microscope was used to capture images.

### Mouse xenograft studies

Four-week-old female BABL/c nude mice acquired from Guangdong Medical Laboratory Animal Center were randomly assigned into two groups, with 6 mice in each group. A total of 5 × 10^6^ MDA-MB-231-shDHX9 or MDA-MB-231-shCtrl cells were inoculated into the right fourth mammary fat pad of each mouse. The tumors were observed every 3 days, and calipers served to quantify the tumor volumes (V = 0.52 × length × width^2^ mm^3^). All the mice were euthanized after 3 weeks of tumor development, and the xenograft tumors were excised for final weight determination. Tumor sections were formalin-fixed, paraffin-embedded, and processed for immunohistochemistry. For the observation of survival time, the humane endpoints were referenced to the *ARENA/OLAW IACUC Guidebook* (2nd edition, 2002) for the management of laboratory animals. The Southern Medical University Animal Care and Use Committee approved all the animal studies.

### Immunohistochemistry (IHC)

Xenograft tumor tissue paraffin blocks were sectioned at 4 μm thickness. After deparaffinization and ethanol hydration, endogenous peroxidase activity was eliminated. After being blocked in 5% BSA for 1 h, the xenograft sections were incubated overnight at 4 °C with primary antibodies against DHX9, LC3, p62, BECN1, Ki-67 and cleaved-caspase3. BC tissue microarrays were probed with DHX9 and BECN1. After three washes with PBS, the sections were exposed to secondary antibodies and developed in DAB (Solarbio). Each slide was assessed based on two parameters: staining intensity (none: 0; weak: 1; moderate: 2; strong: 3) and the proportion of stained cells (positive cells ≤25% of the cells: 1; 26%–50% of the cells: 2; 51%–75% of the cells: 3; ≥75% of the cells: 4) [57]. The product of these two values determined the final score, which ranged from 0 to 12.

### Bioinformatics analysis

The TCGA data were normalized and differentially analyzed using the Deseq2 package. The GEO data were analyzed using the limma package. The threshold value for identifying differentially expressed genes was set as *p* < 0.05. The impact of DHX9 expression on prognosis based on TCGA data was analyzed using survival package and survminer package. Survival analysis was also generated on OSbrca web server [58] and Kaplan Meier plotter [59]. The analysis of histone modification types was conducted according to the ChIP-seq data on TFmapper [60] and UALCAN [61].

### Statistical analysis

All the experiments were replicated three times for each assay. The sample size for each study was determined based on similar well-established literatures, and no statistics methods were employed to pre-specify the required sample size. The quantification of immunoblotting was performed with ImageJ software (USA), while the number of punctate foci in immunofluorescence was determined with Photoshop software (Adobe, California, USA). GraphPad software (version 10.4.2, Massachusetts, USA) was employed to conduct the statistical analysis. Unpaired Student′s t-tests were used to evaluate the significance of the differences between two groups. Paired Student′s t-tests were utilized to analyze the matched clinical data. Kaplan–Meier analysis was performed to generate survival curves, with statistical parameters determined by a log-rank test. All the statistical tests were two-sided and the data were presented as the means ± standard deviations (SDs). Statistical significance was established at *p* < 0.05, denoted as *p < 0.05, **p < 0.01, and ***p < 0.001.

## Supplementary information

Supplemental material 1

Supplemental material 2

Supplementary Materials 3

original western blots

## Data Availability

The datasets supporting the conclusions of this article are available in the GEO DataSets (https://www.ncbi.nlm.nih.gov/gds/?term=) and UCSC XENA (https://xenabrowser.net/datapages/).
