# Supplementary material for: DHX9-mediated epigenetic silencing of BECN1 contributes to impaired autophagy and tumor progression in breast cancer via recruitment of HDAC5
Source: Cell Death Dis. 2025 Jul 14;16(1):524. doi: 10.1038/s41419-025-07847-y (PMC12260095; doi:10.1038/s41419-025-07847-y)
Supplement: Supplementary file 1 — Supplemental material 1 [file 41419_2025_7847_MOESM1_ESM.docx]

**Supplementary Materials 1**

**DHX9-mediated** **epigenetic silencing of BECN1 contributes to** **impaired autophagy and tumor progression in breast cancer via recruitment of HDAC5**

Ziyang Li, Fang Liu, Fengbei Li, Guopeng Zeng, Xin Wen, Jianan Ding, Jueyu Zhou

**This file includes:**

Supplemental figures and figure legends

**Supplemental figures and figure legends**


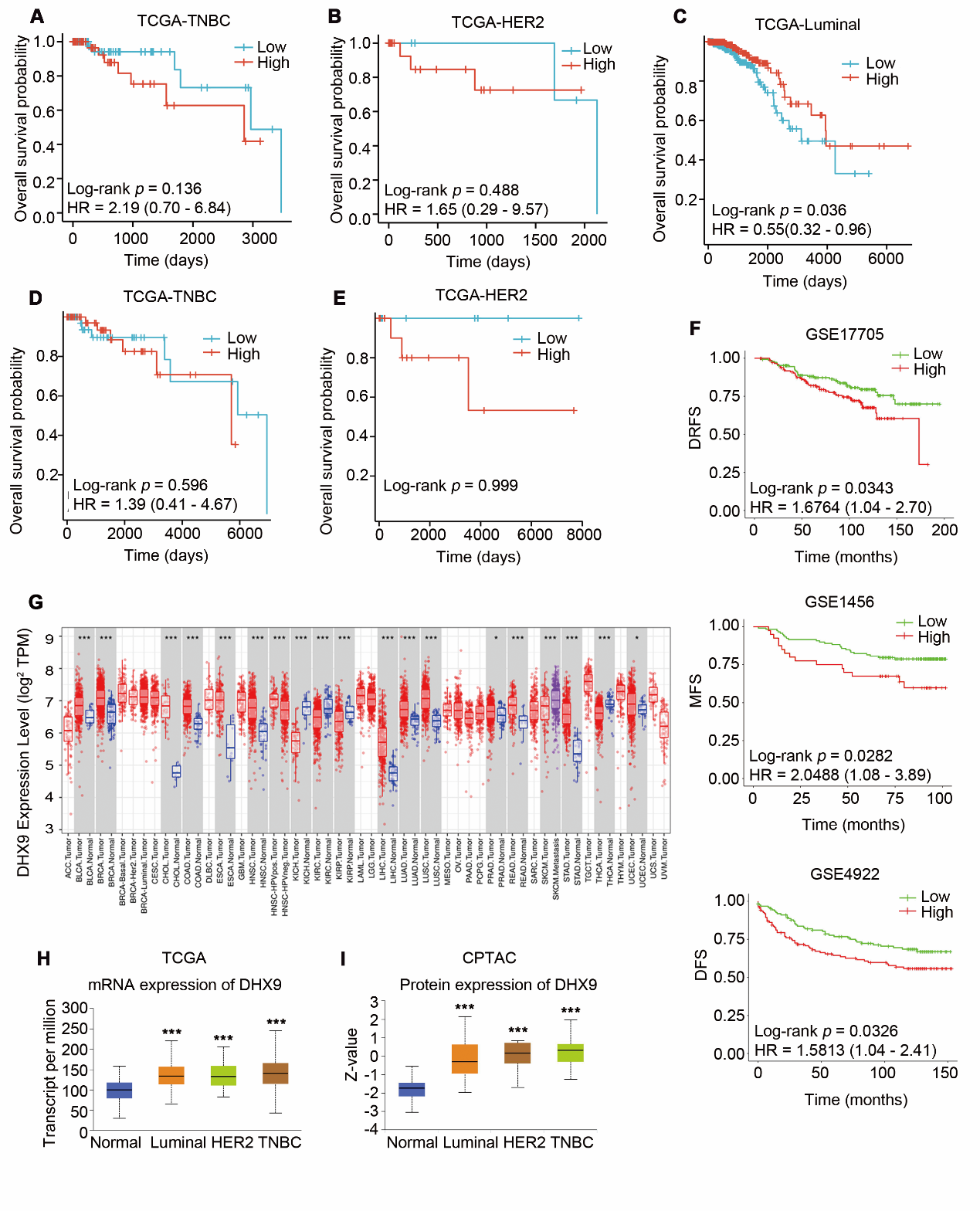


**Fig. S1. DHX9 is highly expressed in breast cancer and indicates poor prognosis.**

**(A-B)** The overall survival probabilities in TNBC **(A)** and HER2+ **(B)** subtypes of breast cancer based on TCGA-BRCA survival data were compared using a log-rank test between DHX9-high and -low expression groups.

**(C-E)** The overall survival probabilities in luminal **(C)**, TNBC **(D)** and HER2+ **(E)** subtypes of breast cancer based on TCGA-BRCA survival data were compared using a log-rank test between DDX39A-high and -low expression groups.

**(F)** Kaplan-Meier survival plots generated using OSbrca online database compared the DRFS, MFS and DFS between DFX9-high and -low expression groups using a log-rank test. DRFS: distant relapse-free survival, DFS: disease-free survival, MFS: metastasis-free survival.

**(G)** Box diagram generated using the Timer online database showing the mRNA expression of DHX9 in pan-cancer.

**(H-I)** Box diagram generated using the UALCAN online database presenting the mRNA **(H)** and protein **(I)** expression of DHX9 in breast cancer subtypes and normal breast tissues.

**p*<0.05, ****p*<0.001 vs. corresponding control.


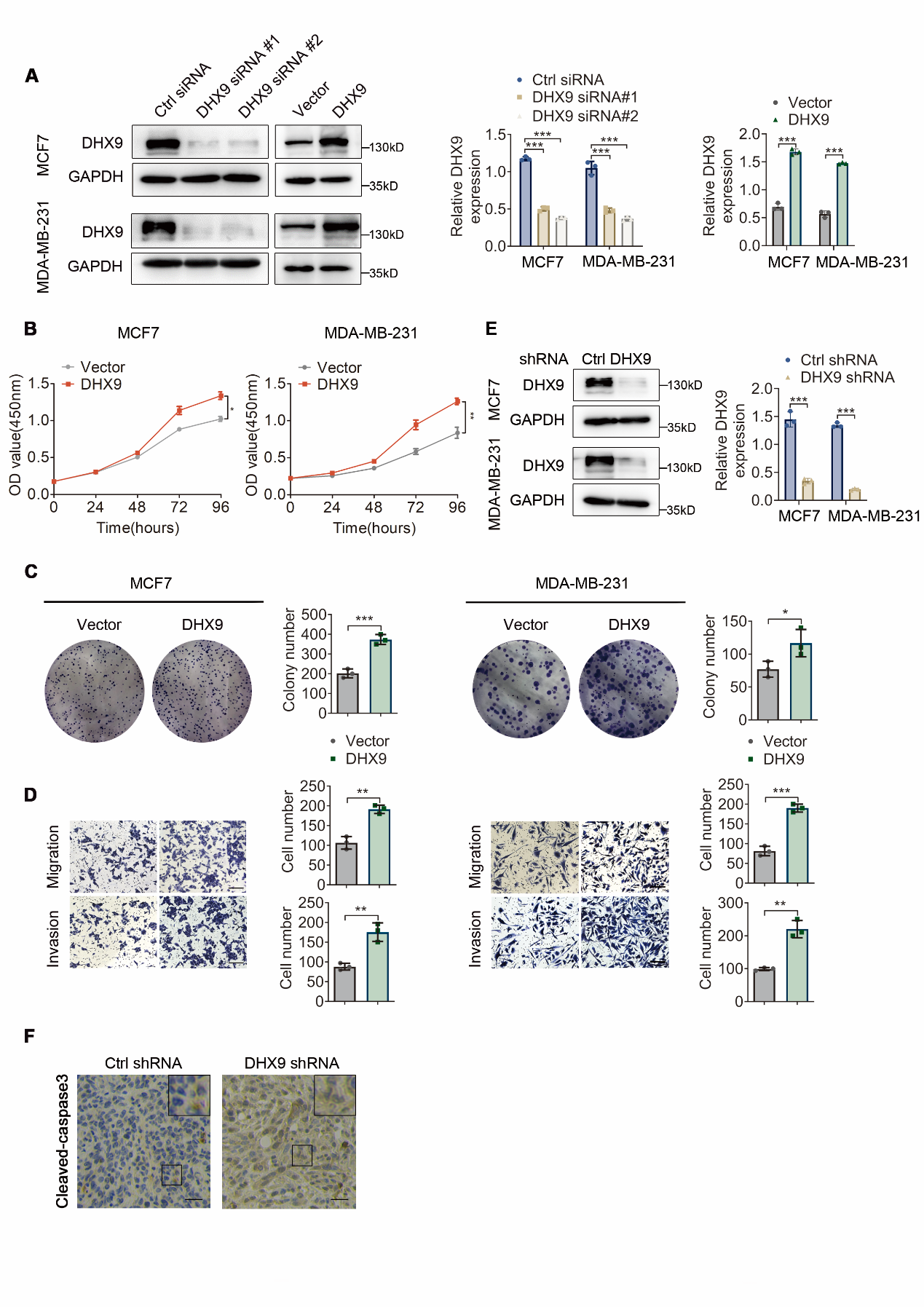


**Fig. S2. DHX9 knockdown impairs breast tumorigenesis *in vitro* and *in vivo*.**

**(A)** Cells were transfected with the indicated siRNAs or plasmids and Western blotting was conducted to verify the transfection efficiency of DHX9 knockdown or overexpression in BC cells. Histograms (Right) showing the relative expression of DHX9 to GAPDH.

**(B-D)** CCK8 **(B)**, colony formation **(C)**, and transwell assays **(D)** were performed after DHX9 overexpression.

**(E)** The knockout efficiency was assessed in stably transfected cells by immunoblotting. Histograms (Right) showing the relative expression of DHX9 to GAPDH.

**(F)** Representative IHC pictures showing the immunostaining of cleaved-caspase3 in mice xenografts (6 mice per group). Scale bars, 25 μm.

Data are representative of three biological independent experiments **(A-E)** and are plotted as the mean ± SD **(A-E)**. *P* values were calculated by unpaired two-tailed Student’s t test **(A-E)**. **p*<0.05, ***p*<0.01, ****p*<0.001 vs. corresponding control.


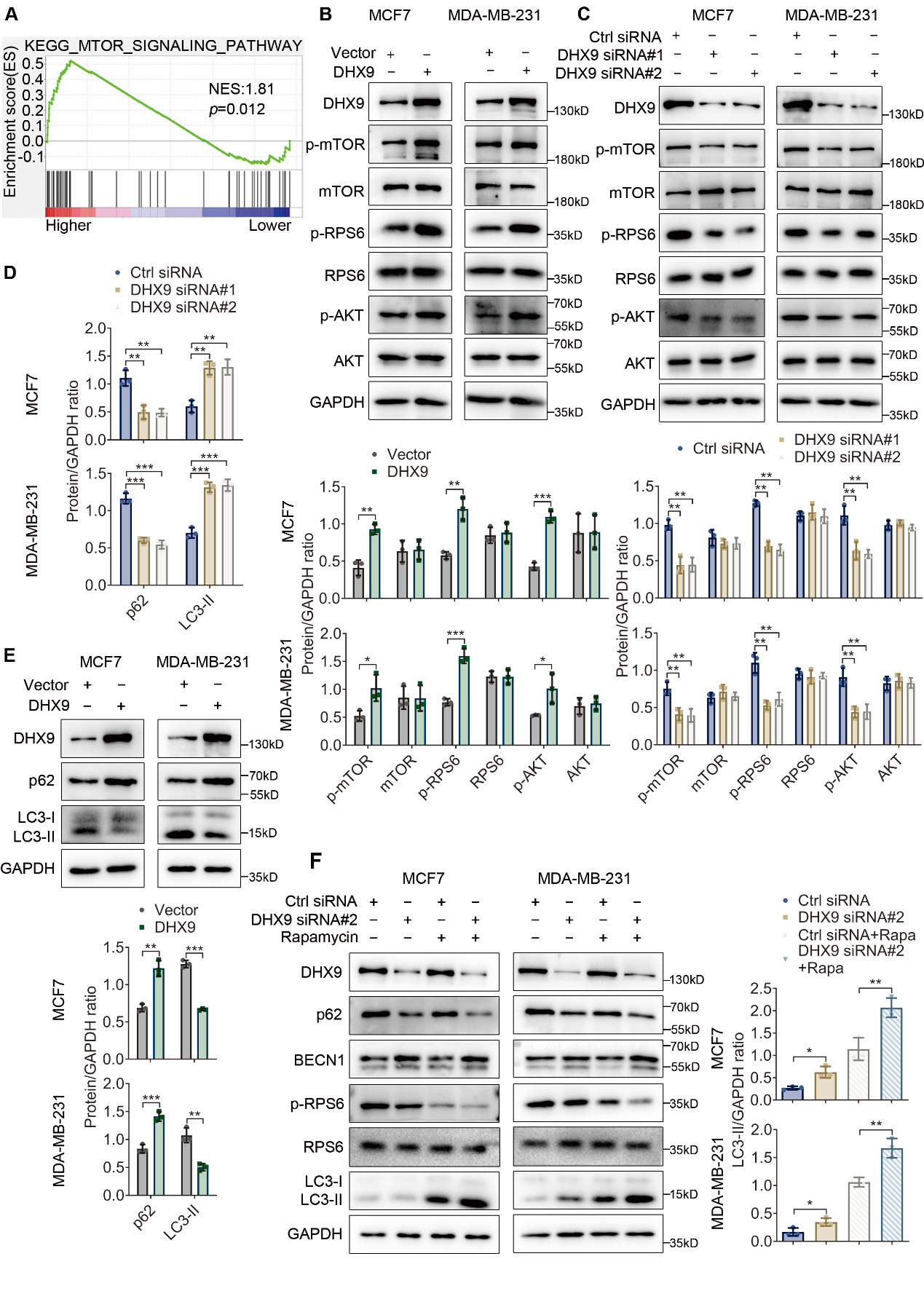


**Fig. S3. DHX9 knockdown induces autophagy.**

**(A)** Gene set enrichment analysis (GSEA) indicated that the mTOR pathway was positively enriched in patients harboring high DHX9 expression from TCGA-BRCA dataset.

**(B)** Immunoblot analysis to assess the protein levels of p-mTOR (s2448), p-RPS6 (Ser 235, 236), p-AKT (Ser473) and the corresponding total protein levels after DHX9 upregulation. Histograms (Below) show the relative expression of proteins to GAPDH.

**(C)** Immunoblot analysis to assess the protein levels of p-mTOR (s2448), p-RPS6 (Ser 235, 236), p-AKT (Ser473) and the corresponding total protein levels after DHX9 downregulation. Histograms (Below) show the relative expression of proteins to GAPDH.

**(D)** Histograms showing the relative expression of p62 and LC3-II to GAPDH following DHX9 silencing.

**(E)** Immunoblot analysis to investigate the protein expression of LC3-II and p62 after DHX9 overexpression. Histograms (Below) show the relative expression of p62 and LC3-II to GAPDH.

**(F)** BC cells transiently decreased DHX9 or control were treated with DMSO or 10 nM Rapamycin for 4 hours. The protein levels of LC3-II, p62, BECN1, p-RPS6, RPS6 and DHX9 were determined by immunoblotting. Histograms (Right) show the relative expression of LC3-II to GAPDH.

Data are representative of three biological independent experiments **(B-F)** and are plotted as the mean ± SD **(B-F)**. *P* values were calculated by unpaired two-tailed Student’s t test **(B-F)**. **p*<0.05, ***p*<0.01, ****p*<0.001 vs. corresponding control.


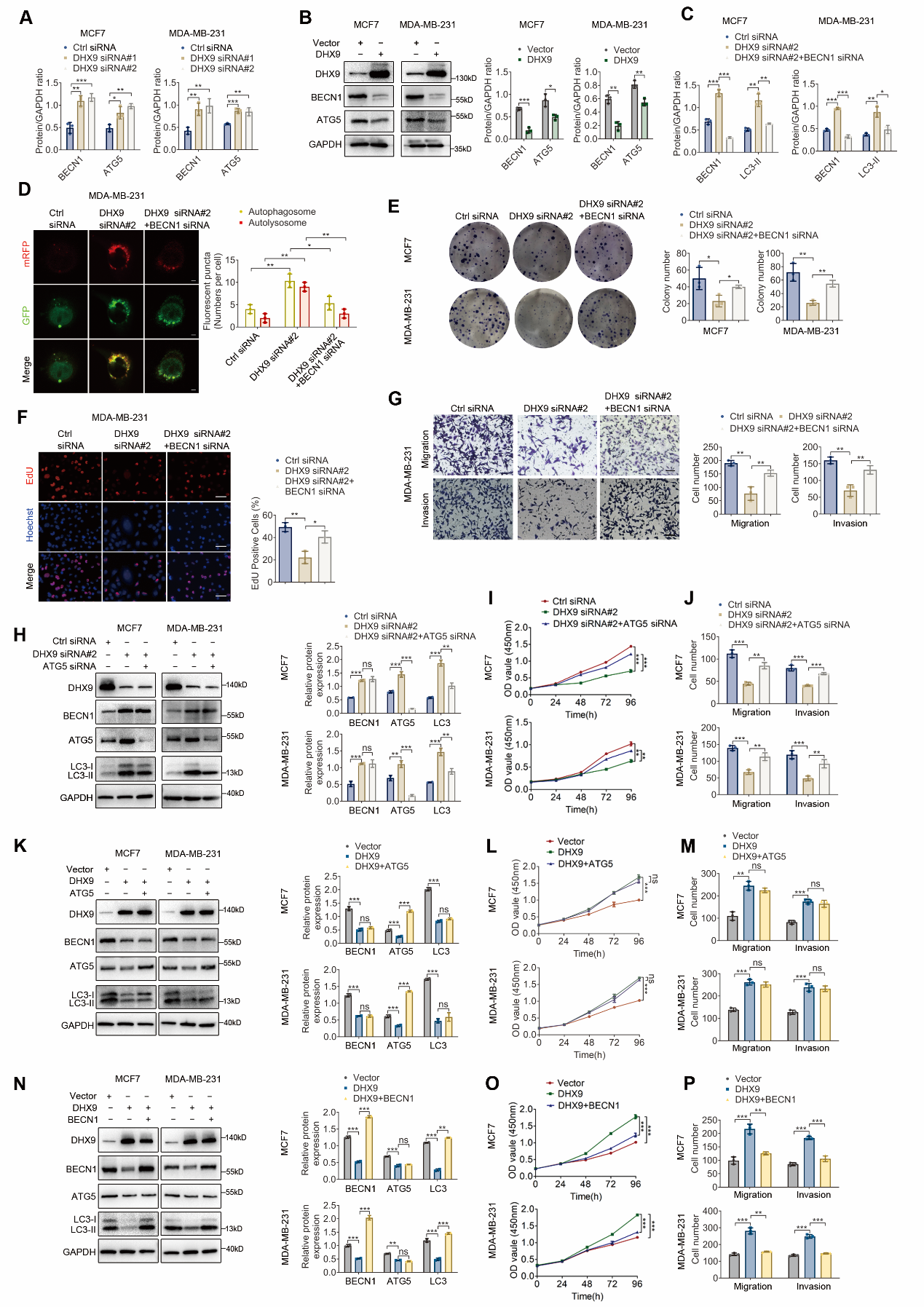


**Fig. S4. BECN1 knockdown reverses the biological effects of DHX9 silencing on BC cells.**

**(A)** Histograms showing the relative expression of BECN1 and ATG5 to GAPDH following DHX9 silencing.

**(B)** Immunoblot analysis to investigate the protein expression of BECN1, ATG5 and DHX9 after DHX9 overexpression. Histograms showing the relative expression of BECN1 and ATG5 to GAPDH.

**(C)** Histograms showing the relative expression of BECN1 and LC3-II to GAPDH after BC cells were transfected with Ctrl siRNA, DHX9 siRNA alone or DHX9 siRNA combined with BECN1 siRNA respectively.

**(D)** MDA-MB-231 was transfected with mRFP-GFP-LC3 after DHX9 silencing or combined silencing of DHX9 and BECN1. Twenty-four hours later, the number of autophagosomes (yellow dots) and autolysosomes (red only dots) per cell were observed and counted. Scale bars, 5 μm.

**(E)** Colony formation assays were conducted after DHX9 downregulation or combined downregulation of DHX9 and BECN1 in MCF7 and MDA-MB-231 cells.

**(F)** EdU positive cells were counted after DHX9 downregulation or combined downregulation of DHX9 and BECN1 in MDA-MB-231 cell. Scale bars, 50 μm.

**(G)** Cell invasion and migration abilities were evaluated after DHX9 downregulation or combined downregulation of DHX9 and BECN1 in MDA-MB-231 cell. Scale bars, 50 μm.

**(H-J)** The effects of knocking down ATG5 on autophagy **(H)**, cell viability **(I)**, migration and invasion abilities **(J)** in DHX9-silenced BC cells were evaluated.

**(K-P)** The effects of overexpressing ATG5 or BECN1 on autophagy, cell viability, migration and invasion abilities in DHX9-overexpressed BC cells were evaluated.

Data are representative of three biological independent experiments **(A-P)** and are plotted as the mean ± SD **(A-P)**. *P* values were calculated by unpaired two-tailed Student’s t test **(A-P)**. **p*<0.05, ***p*<0.01, ****p*<0.001 vs. corresponding control. ns, not significant.


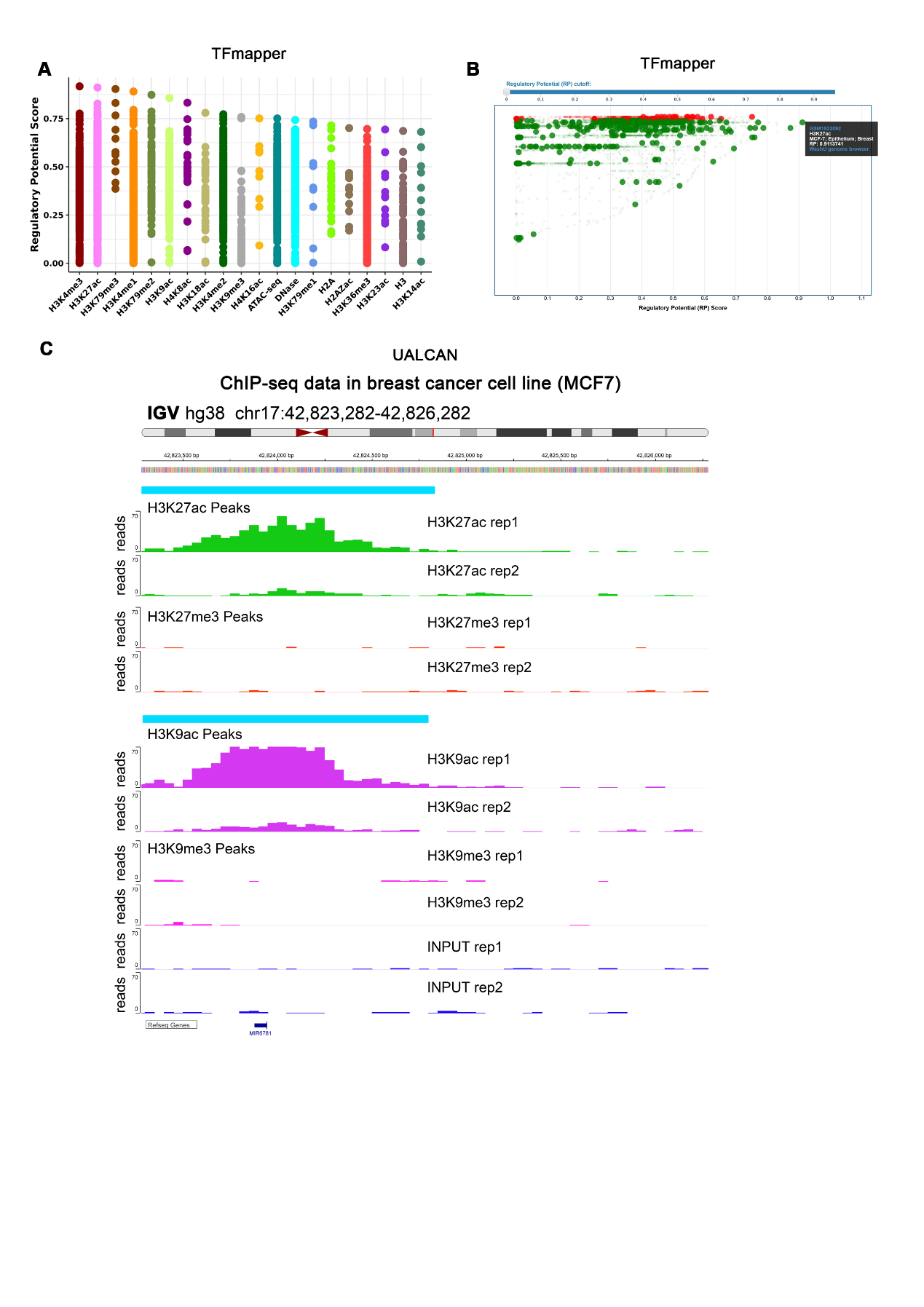


**Fig. S5. ChIP-seq data show the peak of histone H3 acetylation in the BECN1 promoter.**

**(A)** Lattice diagram showing the regulatory potential scores of different H3 modification types of the BECN1 promoter in various biological sources.

**(B)** Lattice diagram showing that the most common H3 modification type in MCF7 is H3K27ac.

**(C)** Representative IGV tracks showing enriched H3 modification types in the BECN1 promoter by ChIP-seq. The blue rectangles represent the H3 modification peaks at the BECN1 promoter.


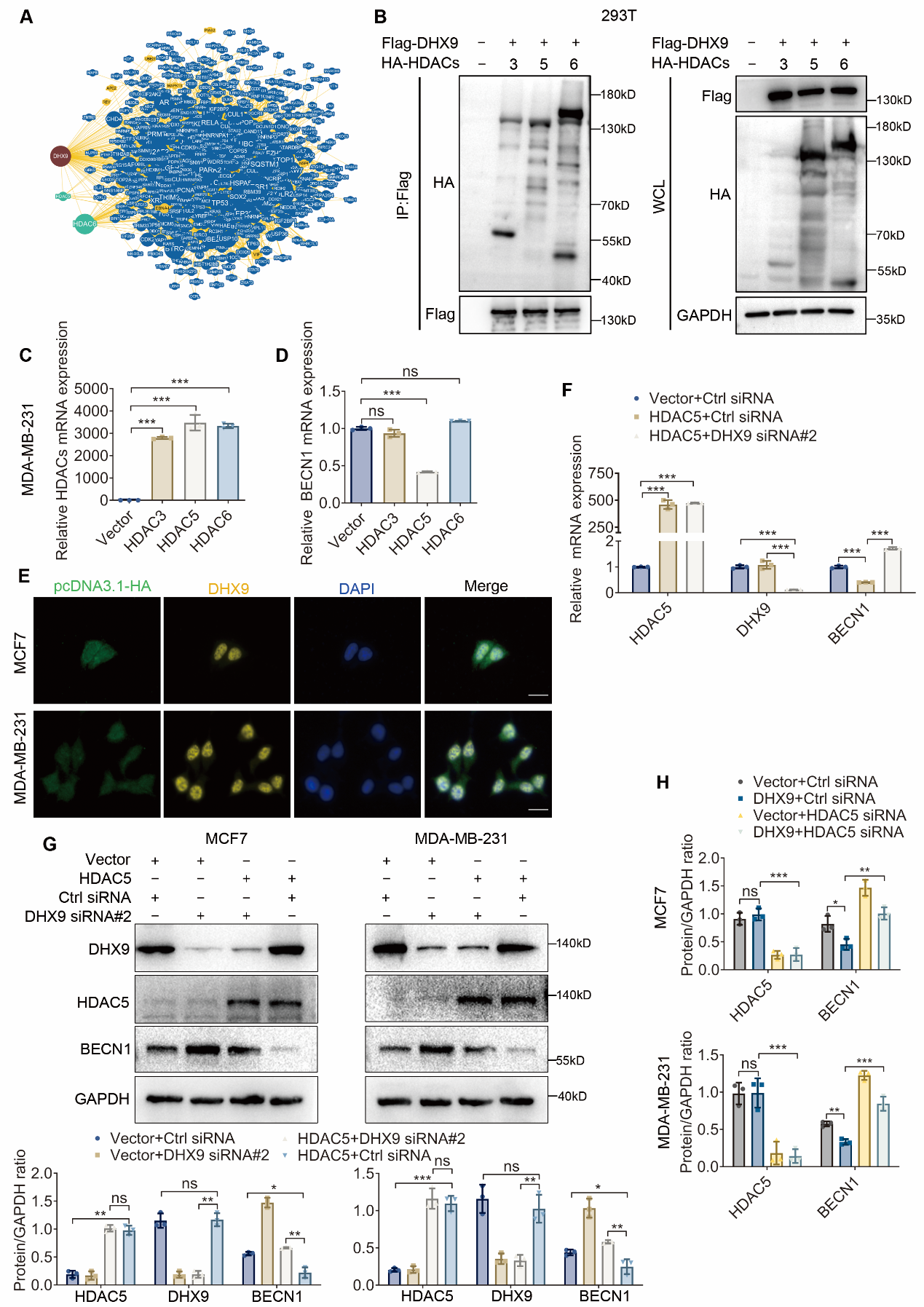


**Fig. S6. DHX9 interacts with HDAC5 and synergistically represses the transcription of BECN1.**

**(A)** The interaction net diagram of DHX9 with its predicted interactors generated using the Biogrid online database. Brown dot: DHX9, green dots: HDACs, blue dots: other potential interactors.

**(B)** 293T cells were co-transfected with Flag-Vector and HA-Vector or Flag-DHX9 and each HA-HDAC, as indicated, for 48 hours. Then the cells were lysed and analyzed by IP using Flag-magnetic beads and Western blotting. WCL: whole cell lysate.

**(C-D)** Quantitative RT-PCR assays were conducted to verify the overexpression efficiency of HDAC3, 5, 6 **(C)**, and to inspect the mRNA expression of BECN1 in MDA-MB-231 **(D)**.

**(E)** Immunofluorescence analyses were executed to investigate the subcellular localization of DHX9 and HA by fluorescence microscopy. Scale bars, 20μm.

**(F)** MDA-MB-231 cell overexpressing Vector or HDAC5 was co-transfected with Ctrl or DHX9 siRNA for 48 hours and then subjected to a quantitative RT-PCR assay.

**(G)** Immunoblot analyses were performed in BC cells overexpressing Vector or HDAC5 after co-transfected with Ctrl or DHX9 siRNA. Histograms (Below) show the relative expression of DHX9, HDAC5 and BECN1 to GAPDH.

**(H)** Histograms showing the relative expression of HDAC5 and BECN1 to GAPDH after BC cells overexpressing Vector or DHX9 were transfected with Ctrl or HDAC5 siRNA.

Data are representative of three biological independent experiments **(B-H)** and are plotted as the mean ± SD **(C, D, F-H)**. *P* values were calculated by unpaired two-tailed Student’s t test **(C, D, F-H)**. **p*<0.05, ***p*<0.01, ****p*<0.001 vs. corresponding control. ns, not significant.


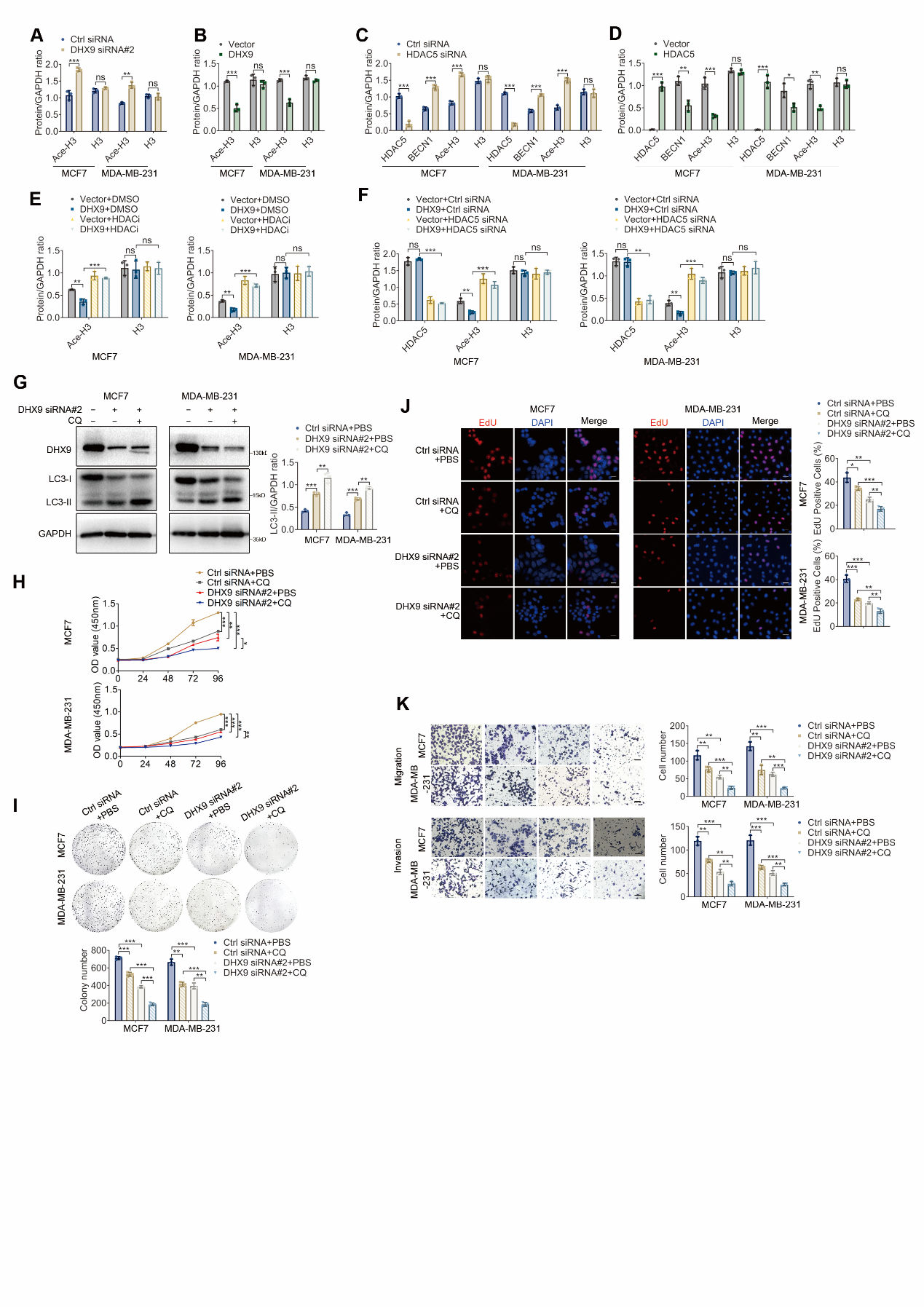


**Fig. S7. DHX9 represses BECN1 transcription via histone deacetylation.**

**(A-B)** Histograms revealing the effects of DHX9 silencing **(A)** or overexpression **(B)** on the acetylation level of histone H3.

**(C-D)** Histograms revealing the effects of HDAC5 silencing **(C)** or overexpression **(D)** on the protein level of BECN1 and the acetylation level of histone H3.

**(E-F)** Histograms revealing DHX9-mediated deacetylation of histone H3 depends on HDAC activity **(E)** or HDAC5 **(F)**.

**(G)** Westen blots were performed in DHX9-silenced BC cells with or without CQ treatment (20µM, 4 hours). Histograms showing the relative expression of LC3-II to GAPDH.

**(H-J)** Cell viability **(H)**, colony number **(I)** and EdU-positive rates **(J)** were evaluated in DHX9-silenced BC cells with or without CQ treatment (15 µM). Scar bars, 20 μm (EdU).

**(K)** Cell invasion and migration abilities were evaluated in DHX9-silenced BC cells with or without CQ treatment (15µM, 96 hours). Scale bars, 50 μm.

Data are representative of three biological independent experiments **(A-K)** and are plotted as the mean ± SD **(A-K)**. *P* values were calculated by unpaired two-tailed Student’s t test **(A-K)**. **p*<0.05, ***p*<0.01, ****p*<0.001 vs. corresponding control. ns, not significant.
