# Supplementary material for: DHX9-mediated epigenetic silencing of BECN1 contributes to impaired autophagy and tumor progression in breast cancer via recruitment of HDAC5
Source: Cell Death Dis. 2025 Jul 14;16(1):524. doi: 10.1038/s41419-025-07847-y (PMC12260095; doi:10.1038/s41419-025-07847-y)
Supplement: Supplementary file 3 — Supplementary Materials 3 [file 41419_2025_7847_MOESM3_ESM.docx]

**Supplementary Materials 3**

**DHX9-mediated** **epigenetic silencing of BECN1 contributes to** **impaired autophagy and tumor progression in breast cancer via recruitment of HDAC5**

Ziyang Li, Fang Liu, Fengbei Li, Guopeng Zeng, Xin Wen, Jianan Ding, Jueyu Zhou

**This file includes:**

Detailed information of primers, siRNAs, antibodies, catalog numbers and company names of reagents and kits

**Primers for plasmid construction**

pcDNA3.1-HA-HDAC3:

5ʹ- cttggtaccgagctcggatccATGGCCAAGACCGTGGCC-3ʹ[forward],

5ʹ- tgctggatatctgcagaattcTTAAACTAATACCTTCCCATTTACTTTTTC-3ʹ [reverse];

pcDNA3.1-HA-HDAC5:

5ʹ- cttggtaccgagctcggatccATGAACTCTCCCAACGAGTCGG-3ʹ[forward],

5ʹ- tgctggatatctgcagaattcTCACAGGGCAGGCTCCTGC-3ʹ [reverse];

pcDNA3.1-HA-HDAC6:

5ʹ- cttggtaccgagctcggatccATGAGTGGAGCGAACCGCG-3ʹ[forward],

5ʹ- tgctggatatctgcagaattcTTAGTGTGGGTGGGGCATATCC-3ʹ [reverse];

pcDNA3.1-flag-BECN1:

5'- cttggtaccgagctcggatccATGGAAGGGTCTAAGACGTCCA -3'[forward],

5'- tgctggatatctgcagaattcTCATTTGTTATAAAATTGTGAGGACACC -3'[reverse];

pcDNA3.1-flag-ATG5:

5'- cttggtaccgagctcggatccATGACAGATGACAAAGATGTGCTTC -3'[forward],

5'- tgctggatatctgcagaattcTCAATCTGTTGGCTGTGGGAT -3'[reverse]

plko.1-GFP-shDHX9:

5ʹ- GAGCCAACUUGAAGGAUUA-3 ʹ

5ʹ- UAAUCCUUCAAGUUGGCUC-3 ʹ

**RNAi treatment**
The oligonucleotide sequences targeting the indicated genes mRNA are as follows:

DHX9 siRNA#1: GUAAAUGAACGUAUGCUGA

DHX9 siRNA#2: GAGCCAACUUGAAGGAUUA

BECN1 siRNA: CAGUUUGGCACAAUCAAUA

HDAC5 siRNA: CAGCAUGACCACCUGACAA

ATG5 siRNA: GCAACUCUGGAUGGGAUUG

Ctrl siRNA: UUCUCCGAACGUGUCACGUTT

**RNA extraction and qRT-PCR**

The primers used to amplify the indicated genes are as follows.

|  | **qRT-PCR Primers** | |
| --- | --- | --- |
| **Gene** | **Forward Sequence (5′-3′)** | **Reverse sequence (5′-3′)** |
| *DHX9* | CTCCACATCTGGCTCTCAAA | TTTTCCAAGGTCCAGTTTCC |
| *BECN1* | CCATGCAGGTGAGCTTCGT | GAATCTGCGAGAGACACCATC |
| *ATG5* | AAAGATGTGCTTCGAGATGTGT | CACTTTGTCAGTTACCAACGTCA |
| *ATG7* | ATGATCCCTGTAACTTAGCCCA | CACGGAAGCAAACAACTTCAAC |
| *ULK1* | GGCAAGTTCGAGTTCTCCCG | CGACCTCCAAATCGTGCTTCT |
| *HDAC3* | TCTGGCTTCTGCTATGTCAACG | CCCGGTCAGTGAGGTAGAAAG |
| *HDAC5* | CTGCGGAACAAGGAGAAGAG | GGGAACTCTGGTCCAAAGAA |
| *HDAC6* | CAACTGAGACCGTGGAGAG | CCTGTGCGAGACTGTAGC |
| *GAPDH* | GCACCGTCAAGGCTGAGAAC | TGGTGAAGACGCCAGTGGA |

**The luciferase reporter assay**

The primers used for cloning the indicated promoters are as follows.

|  | **Primers for promoter clone** | |
| --- | --- | --- |
| **Gene** | **Forward Sequence (5′-3′)** | **Reverse sequence (5′-3′)** |
| *BECN1*  *+1 to -2000* | cgagctcttacgcgtgctagcGCAGAATATAGTTATGTGCAAGCACTT | cagtaccggaatgccaagcttGAAAACTTCCGCCCGAGACC |
| *BECN1*  *-500 to -2000* | cgagctcttacgcgtgctagcGCAGAATATAGTTATGTGCAAGCACTT | cagtaccggaatgccaagcttACAACAACAGCAACAACAAAAGGC |
| *BECN1*  *-1000 to -2000* | cgagctcttacgcgtgctagcGCAGAATATAGTTATGTGCAAGCACTT | cagtaccggaatgccaagcttAGAGATACAGAAAATGGGGTGAGG |
| *BECN1*  *-1500 to -2000* | cgagctcttacgcgtgctagcGCAGAATATAGTTATGTGCAAGCACTT | cagtaccggaatgccaagcttCATGGCTC  ACTGCAGATTTGAC |

**The chromatin immunoprecipitation (ChIP) assay**

The primers for the indicated promoters are as follows.

|  | **ChIP-PCR Primers** | |
| --- | --- | --- |
| **Gene** | **Forward Sequence (5′-3′)** | **Reverse sequence (5′-3′)** |
| *BECN1-1* | GCCTTTTGTTGTTGCTGTTGTTGT | GGTGGGGCACGCCTATAATC |
| *BECN1-2* | ATTATAGGCGTGCCCCACCAC | GGAGGCTCCGCTATTCTCTAAATTC |
| *BECN1-3* | GAATTTAGAGAATAGCGGAGCCTCC | GGAAAACTTCCGCCCGAGAC |
|  | **ChIP-qPCR Primer** |  |
| *BECN1-1* | GCCTTTTGTTGTTGCTGTTGTTGT | GGTGGGGCACGCCTATAATC |

**Antibody Validation**

| **Antibody target** | **Vendor** | **Catalog No.** |
| --- | --- | --- |
| DHX9 | Proteintech | 17721-1-AP |
| DHX9 | Proteintech | 67153-1-Ig |
| BECN1 | Proteintech | 11306-1-AP |
| ATG5 | Proteintech | 10181-2-AP |
| ATG5 | ABclonal | A11427 |
| SQSTM1/p62 | Proteintech | 18420-1-AP |
| LC3 | Proteintech | 14600-1-AP |
| p-mTOR(s2448) | Abmart | T56571 |
| mTOR | Abmart | T55306 |
| p-RPS6(Ser240/244) | CST | 5364 |
| RPS6 | Wanleibio | WL04525 |
| p-Akt (Ser473) | CST | 4060S |
| AKT | CST | 4691S |
| HA | Alpalifebio | KTSM1315 |
| HA Tag Monoclonal antibody | Proteintech | 66006-2-Ig |
| Flag | Sigma | A8592 |
| HDAC5 | Proteintech | 16166-1-AP |
| Ace-Histone H3 | Abcam | Ab47915 |
| Histone H3 | Proteintech | 17168-1-AP |
| GAPDH | Proteintech | 60004-1-Ig |
| Lamin B1 | Proteintech | 66095-1-Ig |
| Ki67 | Proteintech | 27309-1-AP |
| Cleaved-caspase3 | CST | 9661 |
| HRP-conjugated Goat Anti-Rabbit IgG(H+L) | Proteintech | SA00001-2 |
| HRP-conjugated Goat Anti-Mouse IgG(H+L) | Proteintech | SA00001-1 |
| CoraLite488-conjugated Goat Anti-Mouse IgG(H+L) | Proteintech | SA00013-1 |
| Cy3–conjugated Goat Anti-Rabbit IgG(H+L) | Proteintech | SA00009-2 |

**Catalog numbers and company names of reagents and kits**

| **Reagents** | **Vendor** | **Catalog No.** |
| --- | --- | --- |
| DMEM/F12 | ThermoFisher Scientific | 11320033 |
| Fetal bovine serum | Gibco, ThermoFisher Scientific | 16000044 |
| Penicillin-streptomycin | Gibco, ThermoFisher Scientific | 15140122 |
| DMEM | Gibco, ThermoFisher Scientific | 11965092 |
| Bafilomycin A1 | Selleck | S1413 |
| Rapamycin | Selleck | S1039 |
| Chloroquine | Sigma aldrich | C6628 |
| 5-Aza-CdR | Selleck | S1200 |
| HDAC inhibitor | Biyuntian | P1112 |
| CCK-8 | Apexbio | K1018 |
|  |  |  |
| Cell culture insert | BD, Corning | 353097 |
| Matrigel | BD, Corning | 356234 |
|  |  |  |
| EdU assay kit | Biyuntian | C0075S |
| Lipofectamine 2000 | Invitrogen, ThermoFisher Scientific | 11668019 |
| RNAiso Plus | Accurate biology | AG21101 |
| cDNA Synthesis SuperMix kit | Yeasen | 11123ES60 |
| qPCR SYBR Green Master Mix | Yeasen | 11202ES08 |
| RIPA lysis buffer | Biyuntian | P0013B |
| 5×loading buffer | Biyuntian | P0286 |
| Protease inhibitors | Biyuntian | P1045 |
| NE-PER Nuclear and cytoplasmic Extraction Reagents | ThermoFisher Scientific | 78835 |
| Protein A/G magnetic beads | Selleck | B23202 |
| Dual Luciferase Assay kit | Vazyme | DL101-01 |
| ChIP kit | Biyuntian | P2080S |
| Formaldehyde | Macklin | F809702-500ml |
| Dithiothreitol | Solarbio | D8220 |
|  |  |  |
|  |  |  |
| Cell culture slide | NEST | 801010 |
|  |  |  |
|  |  |  |
| DAB kit | Solarbio | DA1016 |
