# Supplementary material for: DHX9-mediated epigenetic silencing of BECN1 contributes to impaired autophagy and tumor progression in breast cancer via recruitment of HDAC5
Source: Cell Death Dis. 2025 Jul 14;16(1):524. doi: 10.1038/s41419-025-07847-y (PMC12260095; doi:10.1038/s41419-025-07847-y)
Supplement: Supplementary file 4 — original western blots [file 41419_2025_7847_MOESM4_ESM.pdf]

Fig.1I

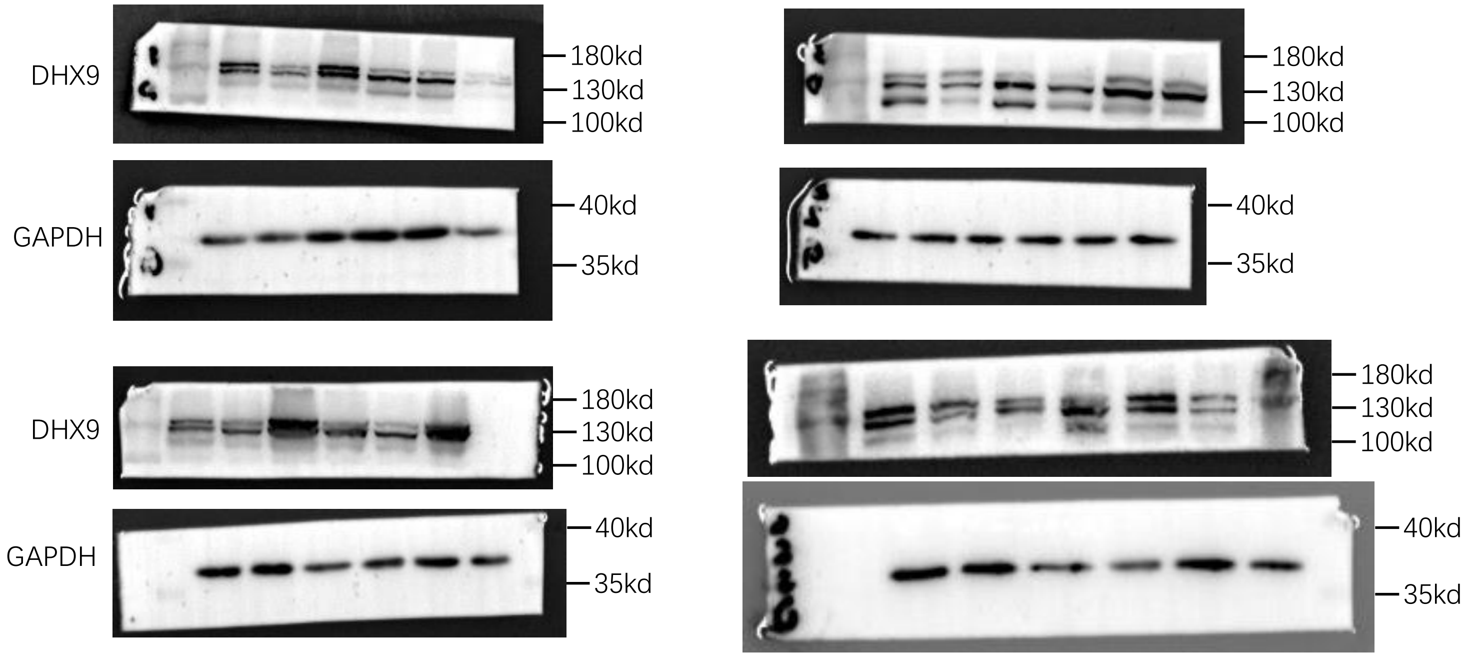

DHX9
180kd
130kd
100kd
GAPDH
40kd
35kd
DHX9
180kd
130kd
100kd
GAPDH
40kd
35kd
180kd
130kd
100kd
40kd
35kd
180kd
130kd
100kd
40kd
35kd

Fig.1J

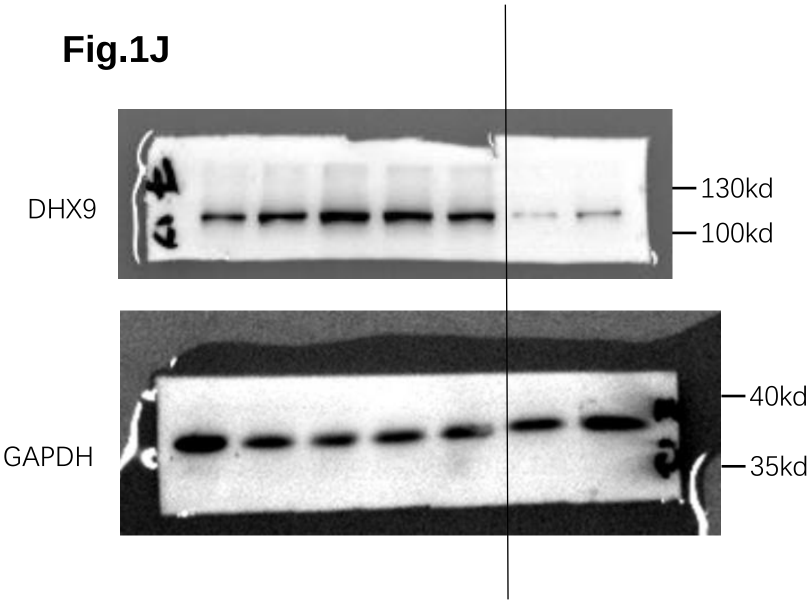

DHX9
130kd
100kd
GAPDH
40kd
35kd

Fig. 3A

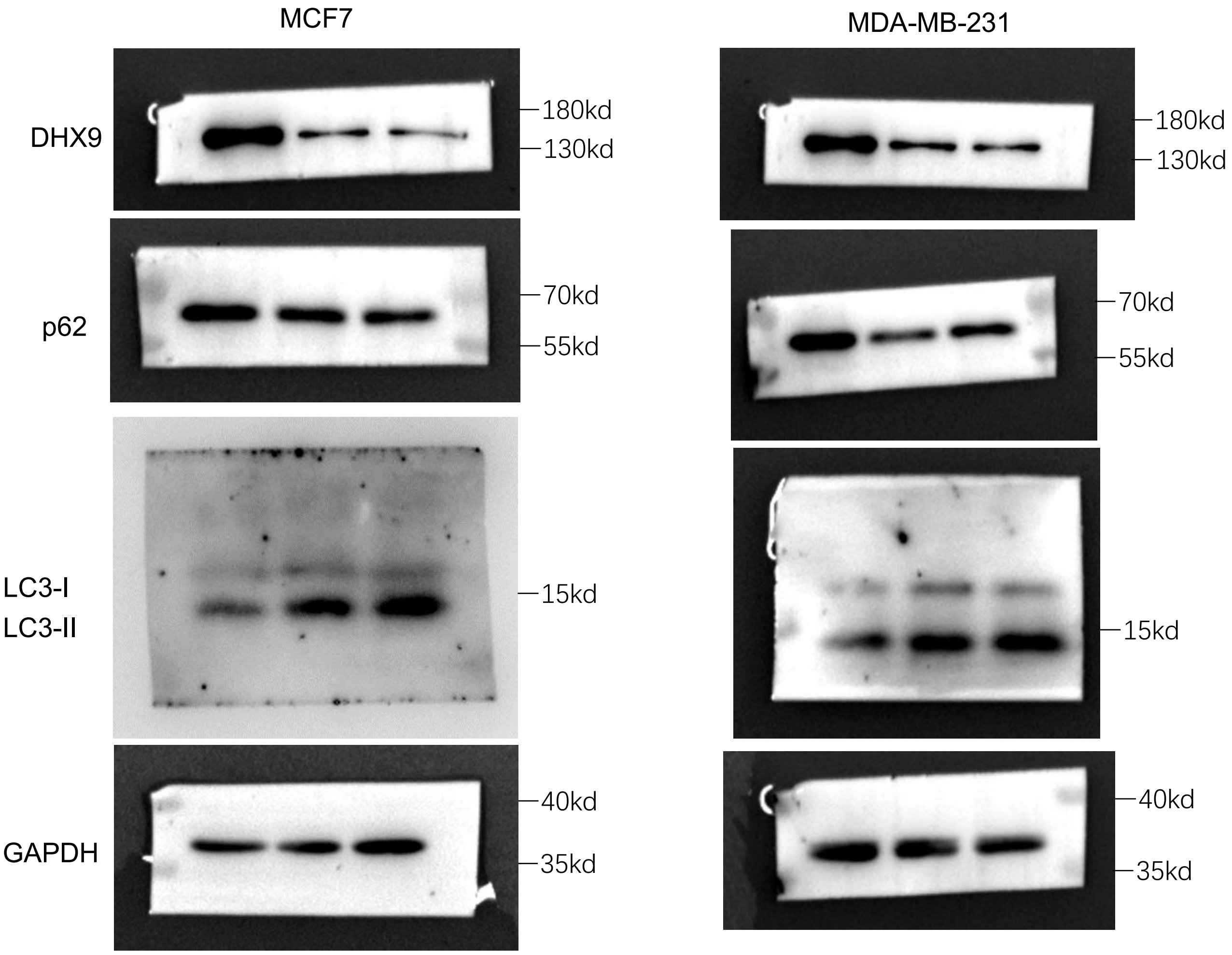
MCF7
MDA-MB-231
DHX9
p62
LC3-I
LC3-II
GAPDH
180kd
130kd
70kd
55kd
15kd
40kd
35kd

Fig. 3B

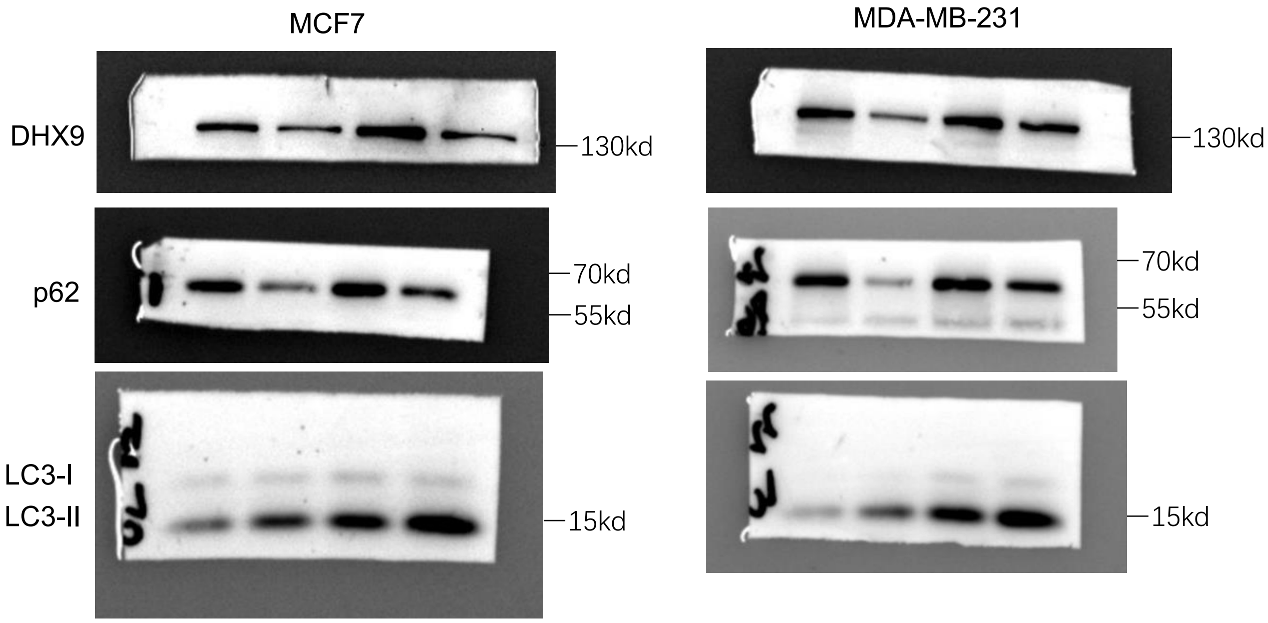
MCF7
MDA-MB-231
DHX9
p62
LC3-I
LC3-II
130kd
70kd
55kd
15kd

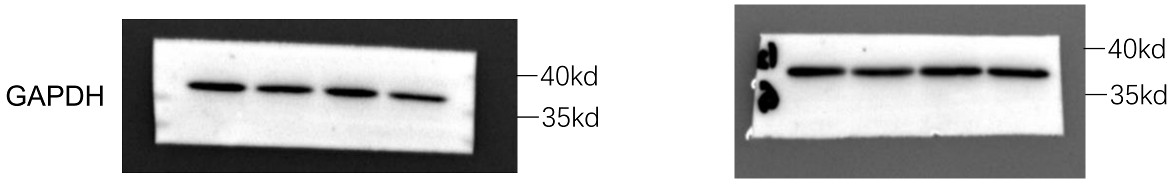
GAPDH
40kd
35kd
40kd
35kd

Fig.3C

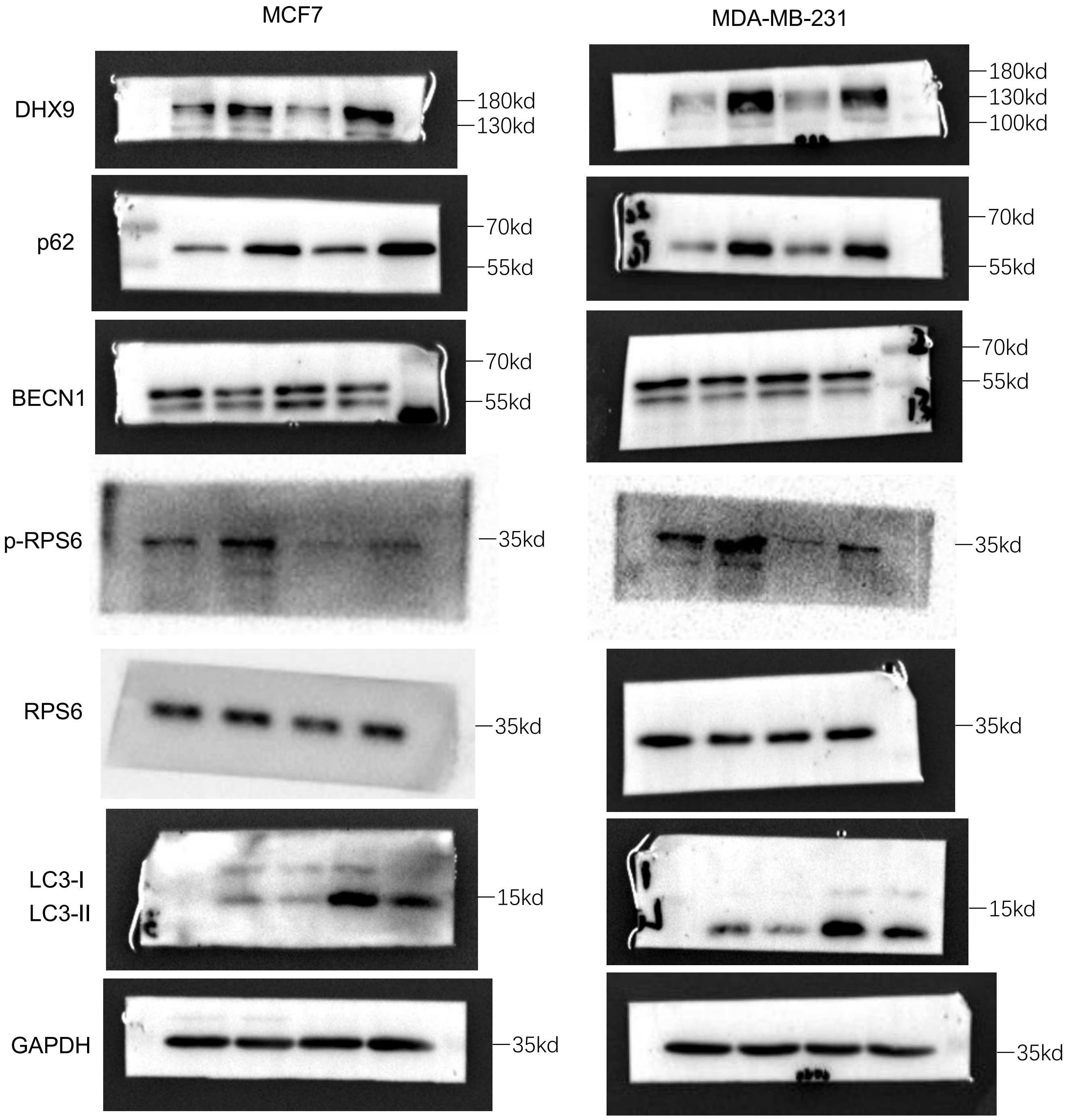
MCF7
MDA-MB-231
DHX9
180kd
130kd
180kd
130kd
100kd
p62
70kd
55kd
70kd
55kd
BECN1
70kd
55kd
70kd
55kd
p-RPS6
35kd
35kd
RPS6
35kd
35kd
LC3-I
LC3-II
15kd
15kd
GAPDH
35kd
35kd

Fig. 4B

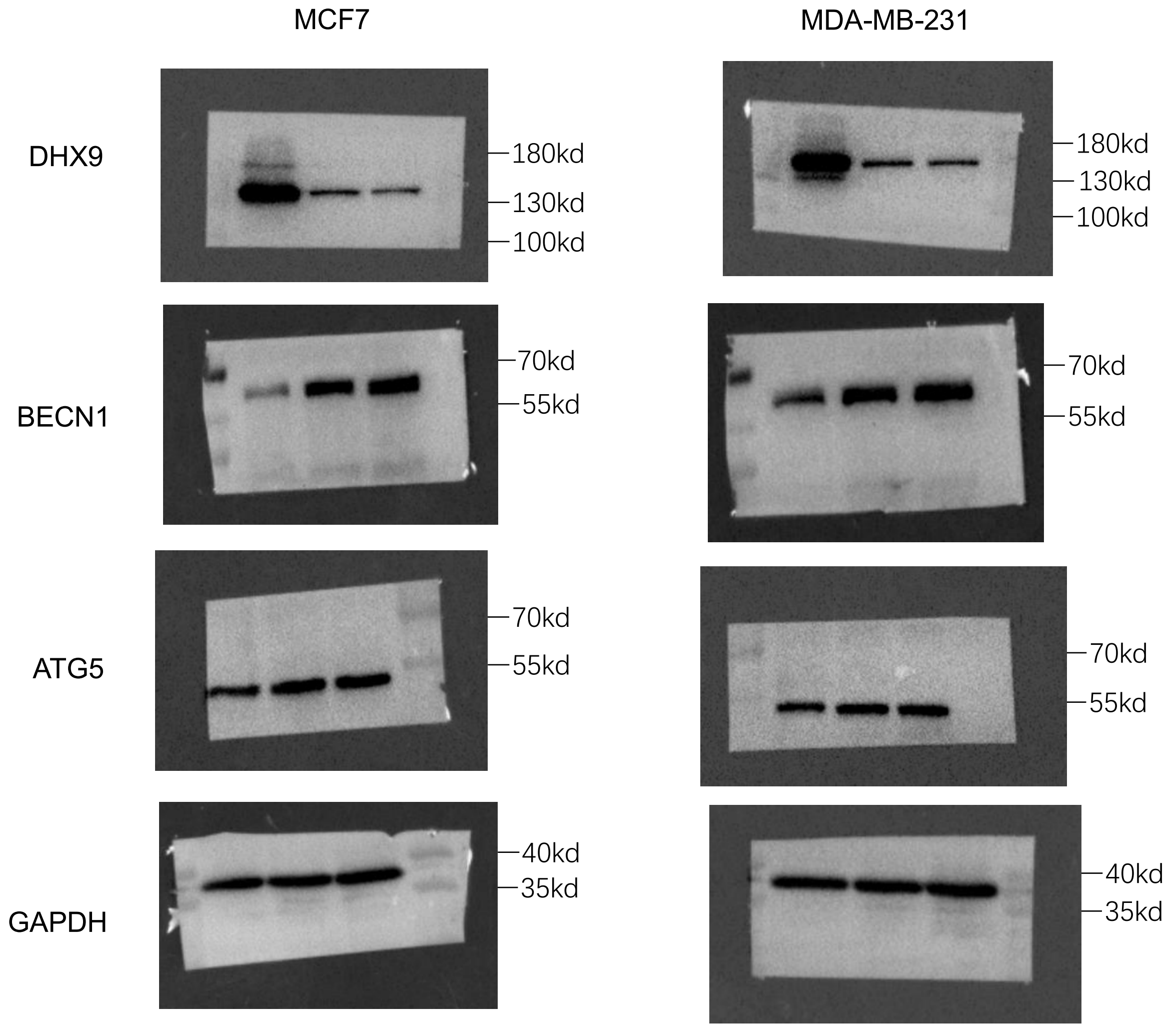

MCF7
MDA-MB-231
DHX9
BECN1
ATG5
GAPDH
180kd
130kd
100kd
70kd
55kd
40kd
35kd

Fig. 4E

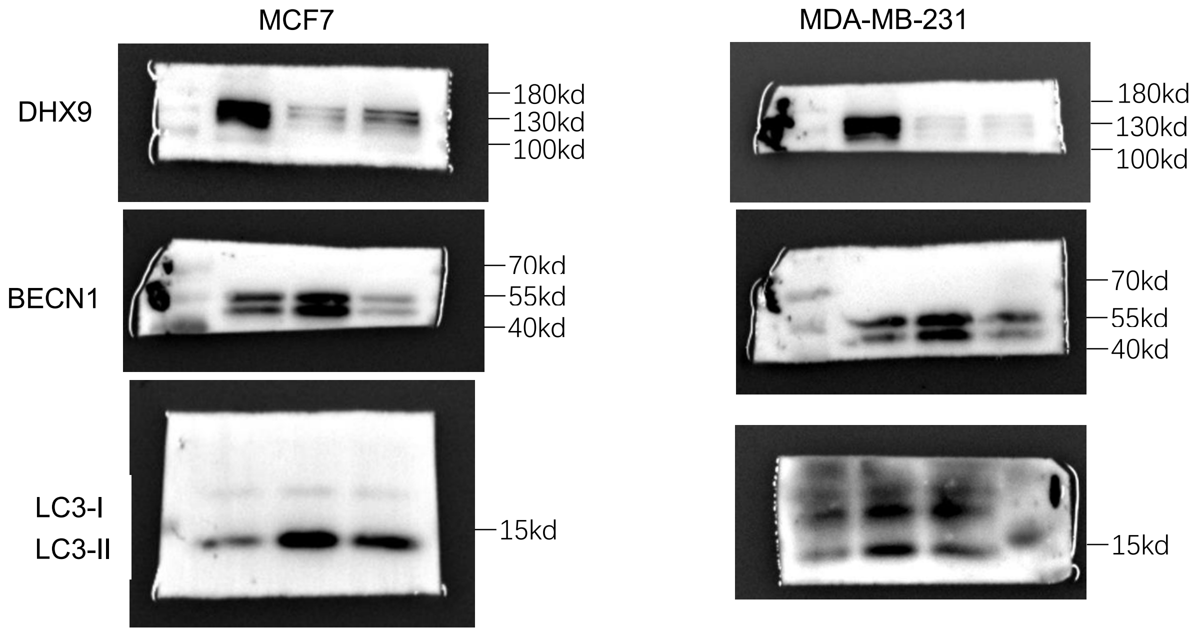

MCF7
MDA-MB-231
DHX9
BECN1
LC3-I
LC3-II
180kd
130kd
100kd
70kd
55kd
40kd
15kd

GAPDH

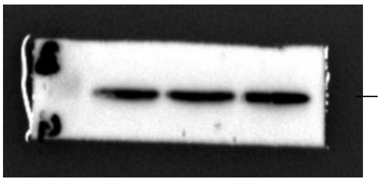

35kd

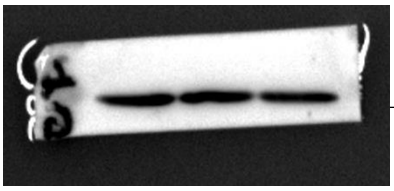

35kd

Fig. 5H

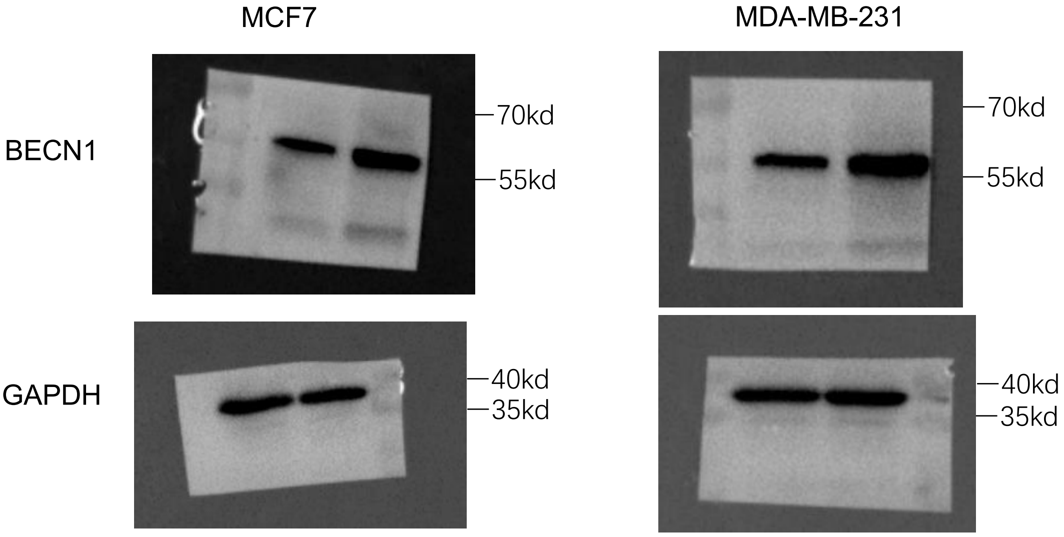
MCF7
MDA-MB-231
BECN1
70kd
55kd
70kd
55kd
GAPDH
40kd
35kd
40kd
35kd

Fig. 5J

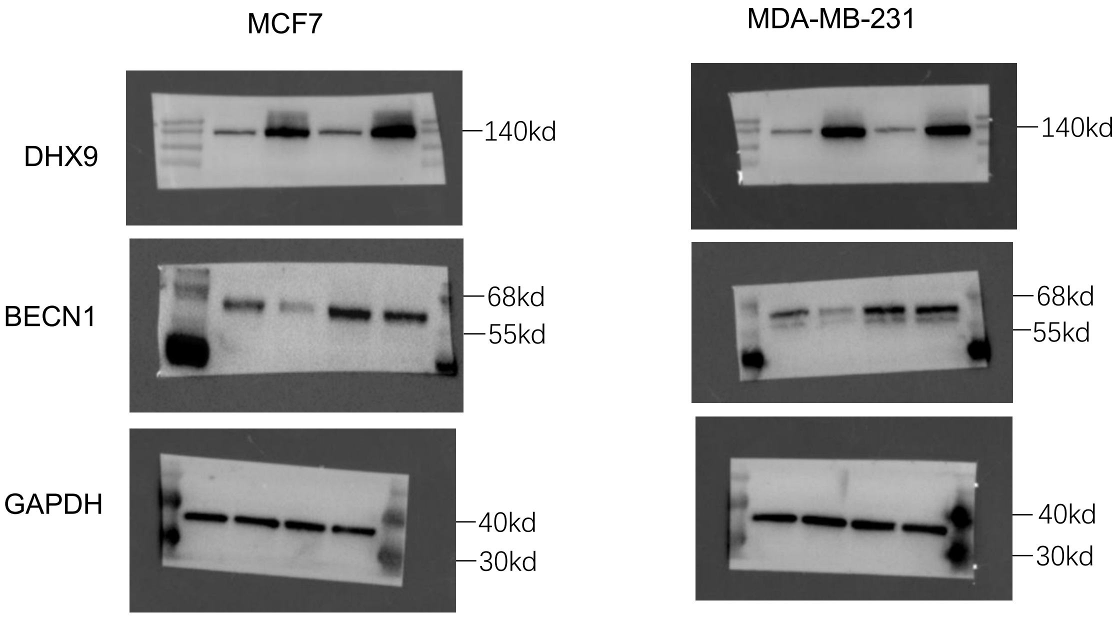
MCF7
MDA-MB-231
DHX9
140kd
140kd
BECN1
68kd
55kd
68kd
55kd
GAPDH
40kd
30kd
40kd
30kd

Fig. 6C

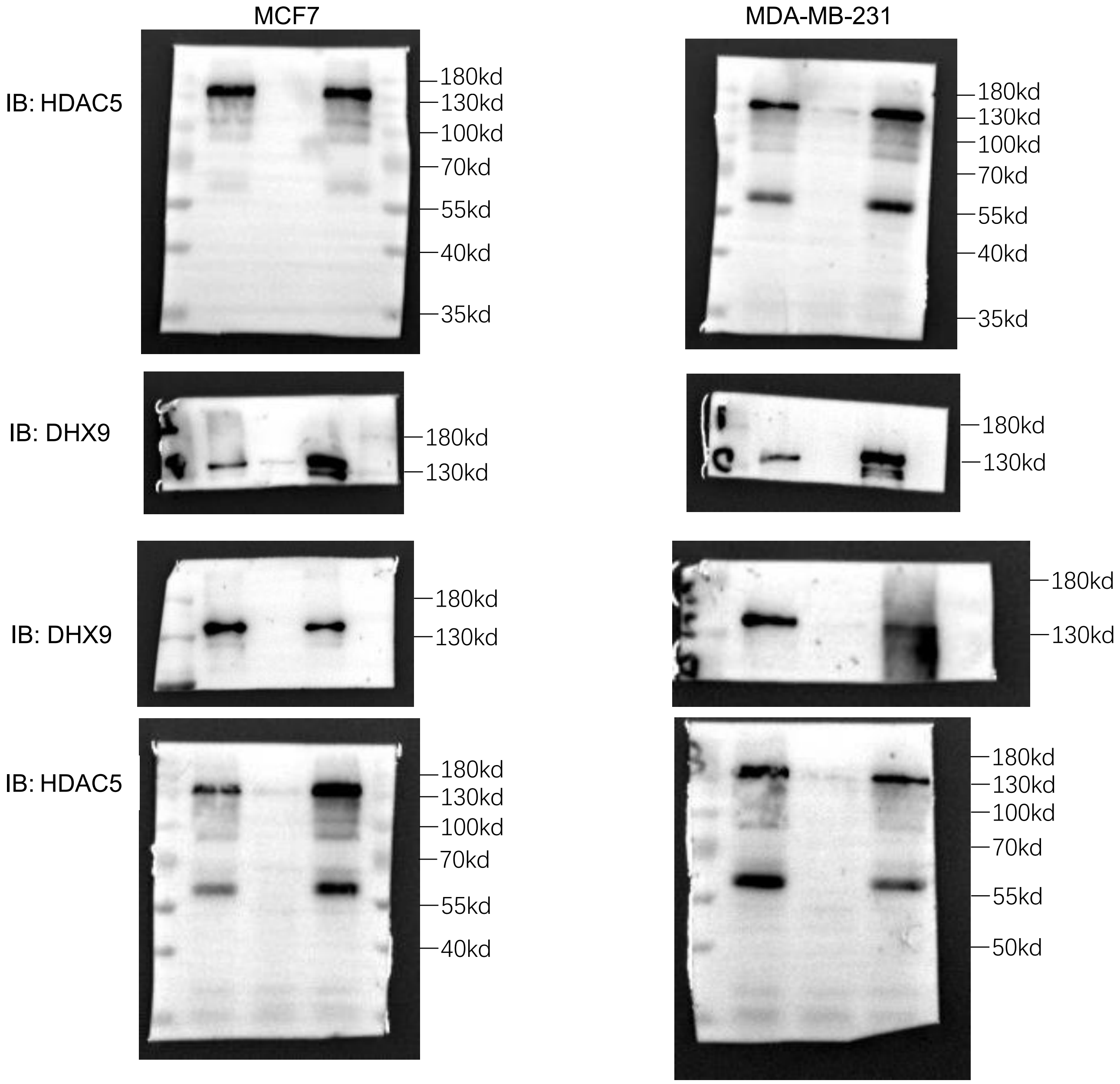
MCF7
MDA-MB-231
IB: HDAC5
IB: DHX9
IB: DHX9
IB: HDAC5
180kd
130kd
100kd
70kd
55kd
50kd
40kd
35kd

Fig. 6G

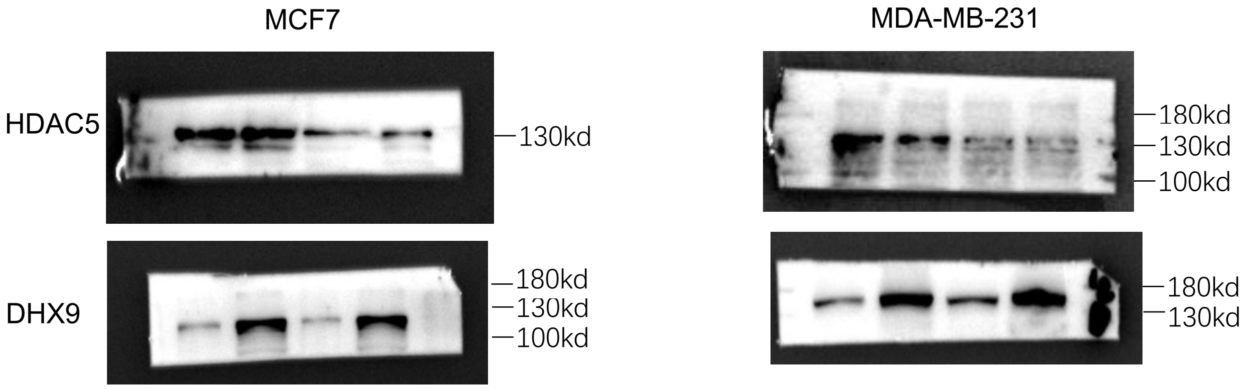
MCF7
MDA-MB-231
HDAC5
DHX9
180kd
130kd
100kd

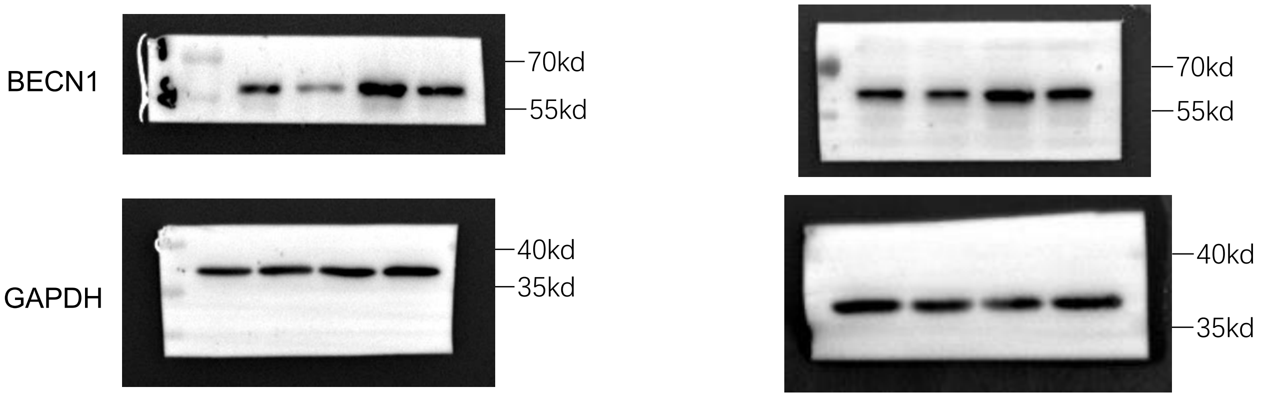

BECN1
70kd
55kd
GAPDH
40kd
35kd
70kd
55kd
40kd
35kd

Fig. 6J

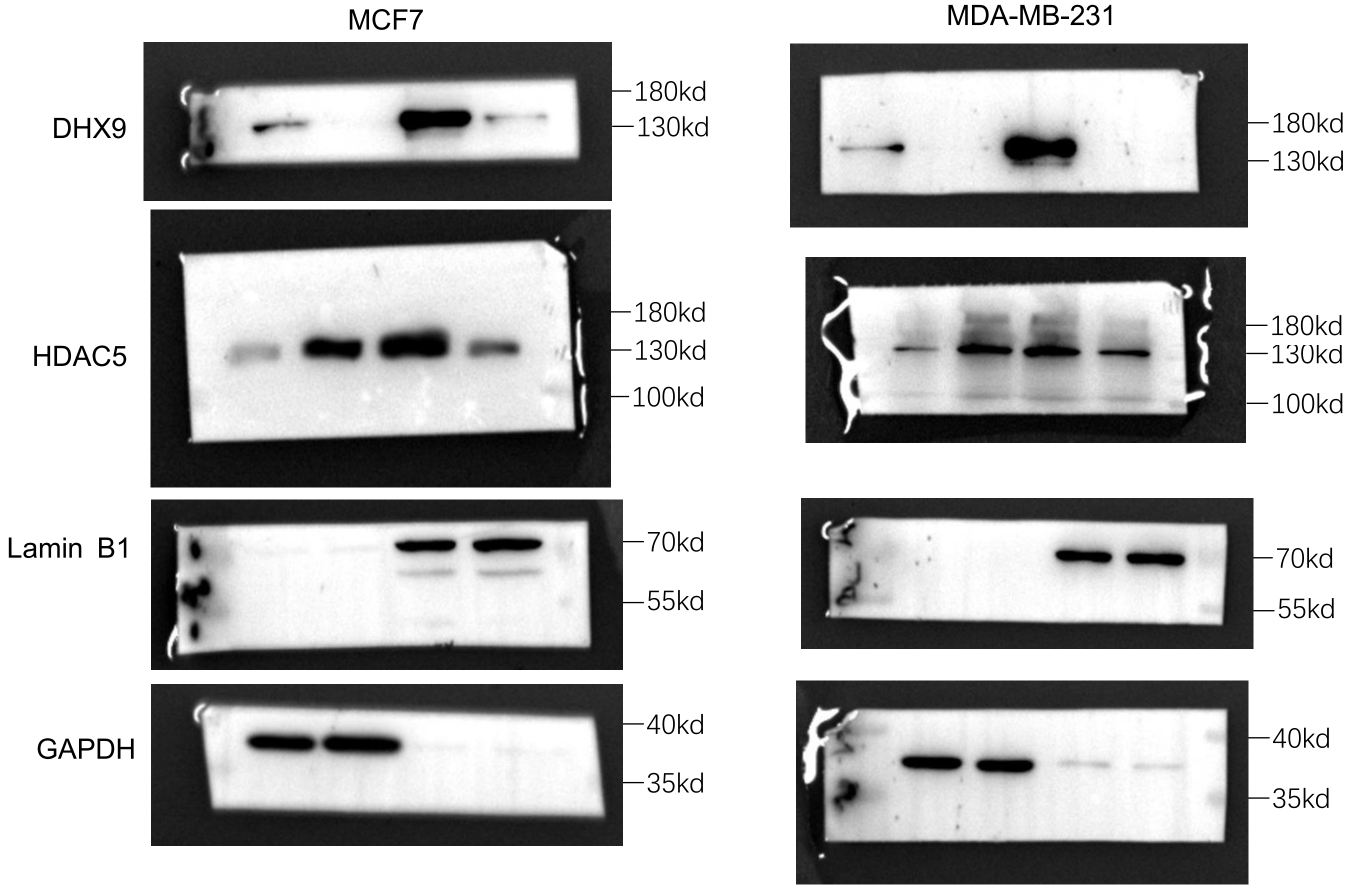

MCF7
MDA-MB-231
DHX9
180kd
130kd
HDAC5
180kd
130kd
100kd
Lamin B1
70kd
55kd
GAPDH
40kd
35kd

Fig. 7B

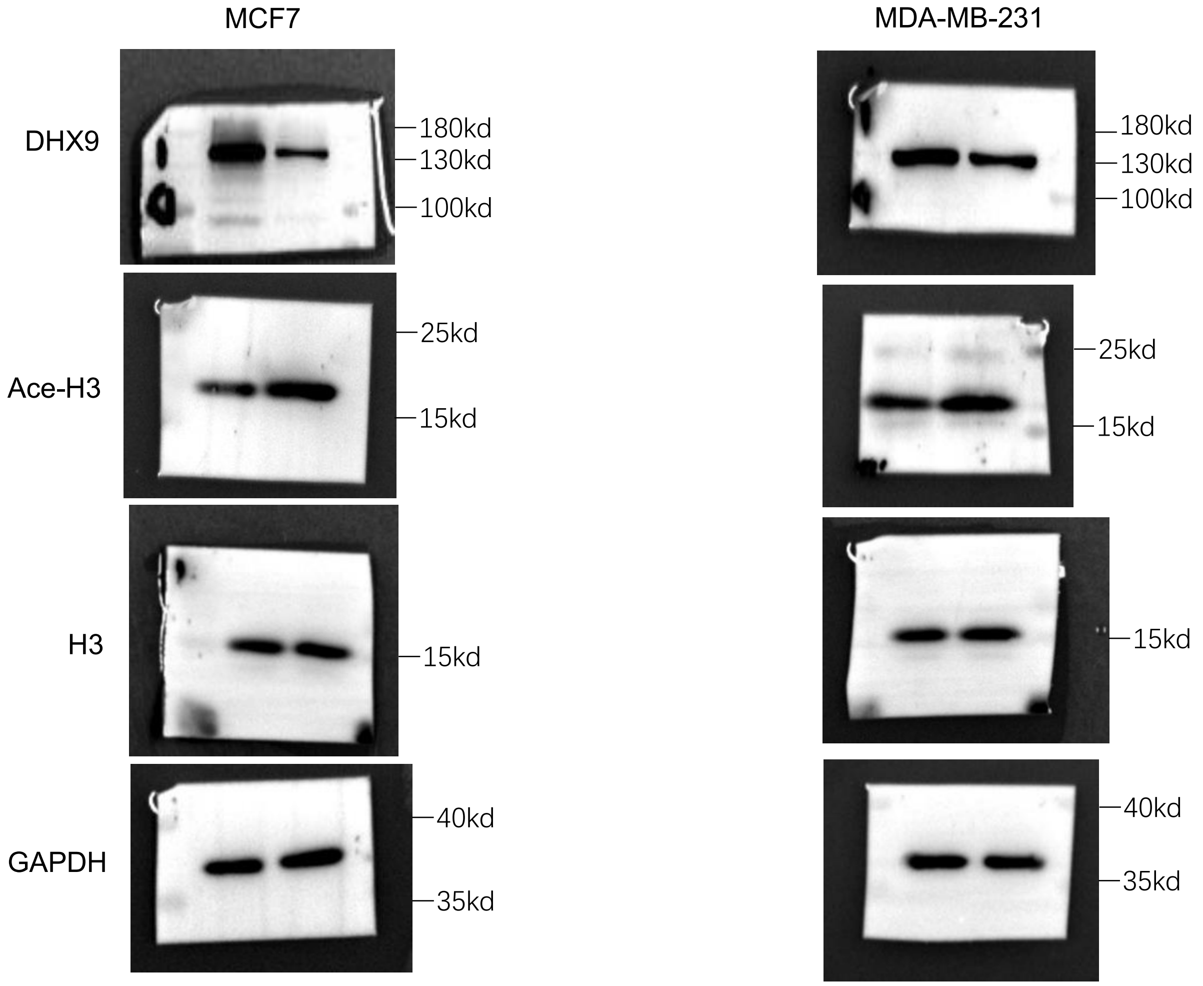

MCF7
MDA-MB-231
DHX9
Ace-H3
H3
GAPDH
180kd
130kd
100kd
25kd
15kd
40kd
35kd

Fig. 7C

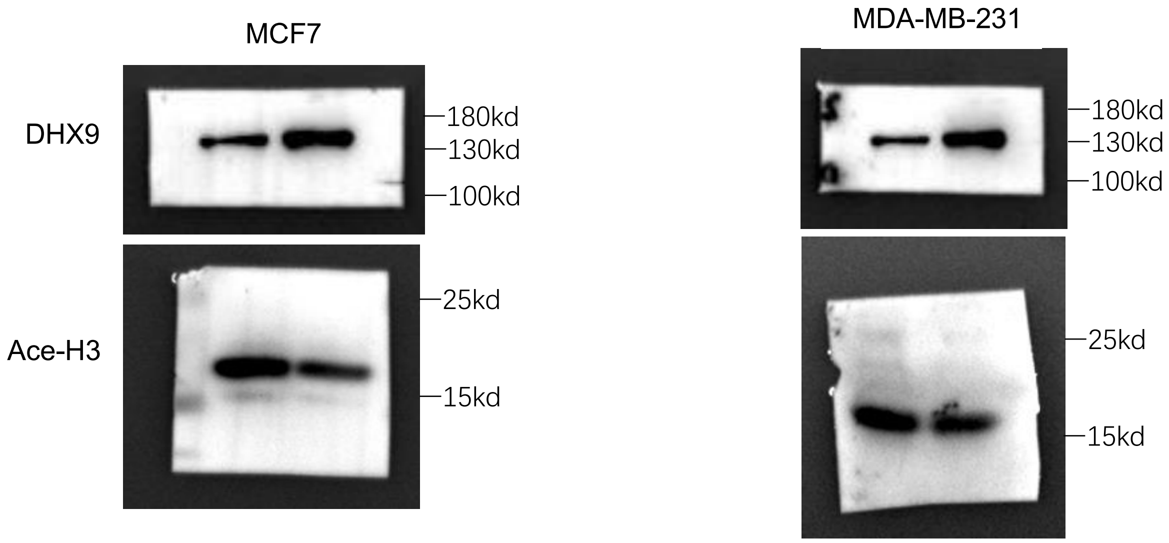

MCF7
MDA-MB-231
DHX9
Ace-H3
180kd
130kd
100kd
25kd
15kd

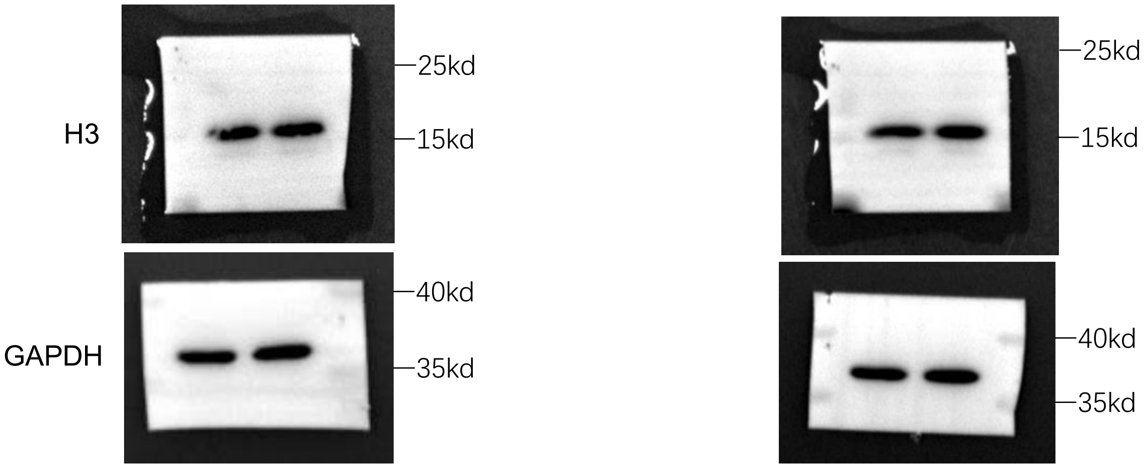
H3
25kd
15kd
GAPDH
40kd
35kd
25kd
15kd
40kd
35kd

Fig. 7D

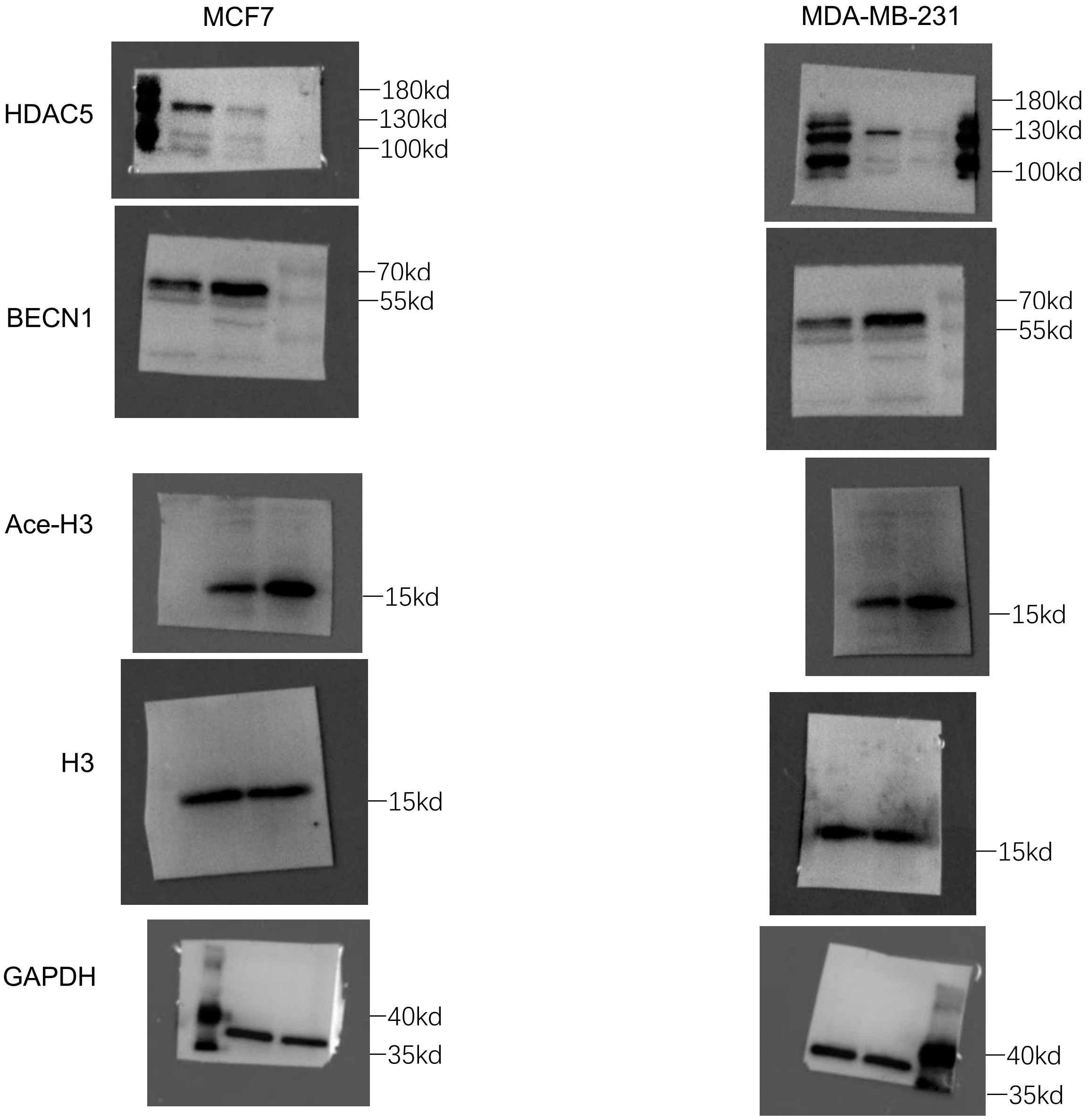
MCF7
MDA-MB-231
HDAC5
180kd
130kd
100kd
BECN1
70kd
55kd
Ace-H3
15kd
H3
15kd
GAPDH
40kd
35kd
180kd
130kd
100kd
70kd
55kd
15kd
15kd
40kd
35kd

Fig. 7E

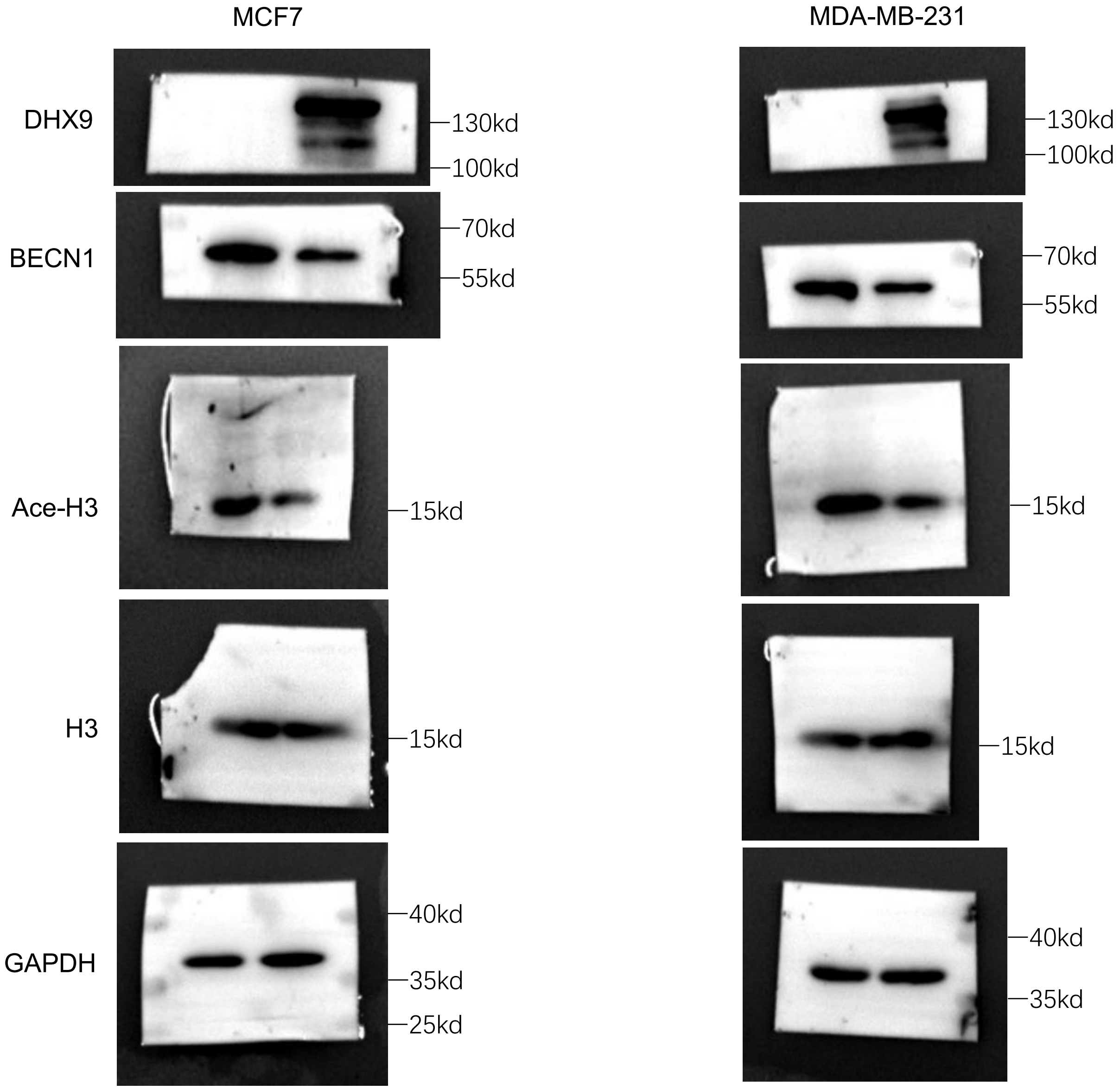
MCF7
MDA-MB-231
DHX9
BECN1
Ace-H3
H3
GAPDH
130kd
100kd
70kd
55kd
15kd
40kd
35kd
25kd

Fig. 7F

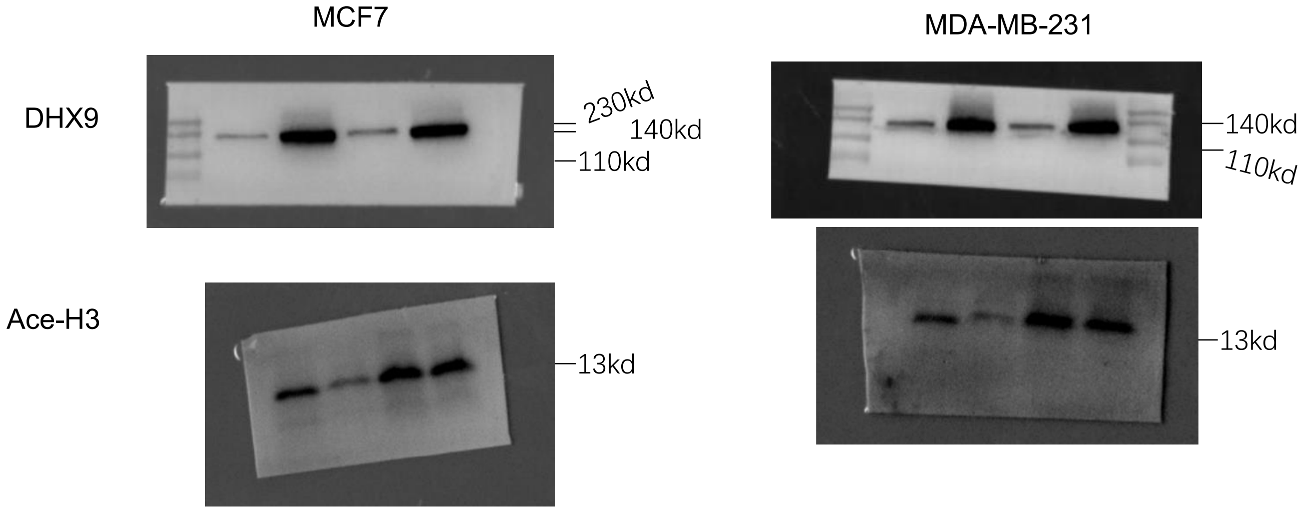
MCF7
MDA-MB-231
DHX9
Ace-H3
230kd
140kd
110kd
13kd

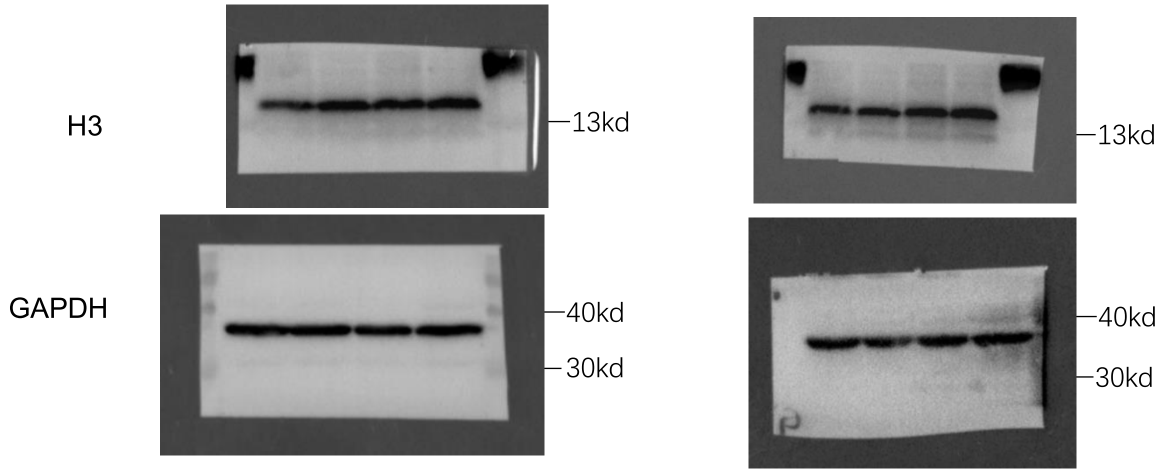
H3
13kd
13kd
GAPDH
40kd
30kd
40kd
30kd

Fig. 7G

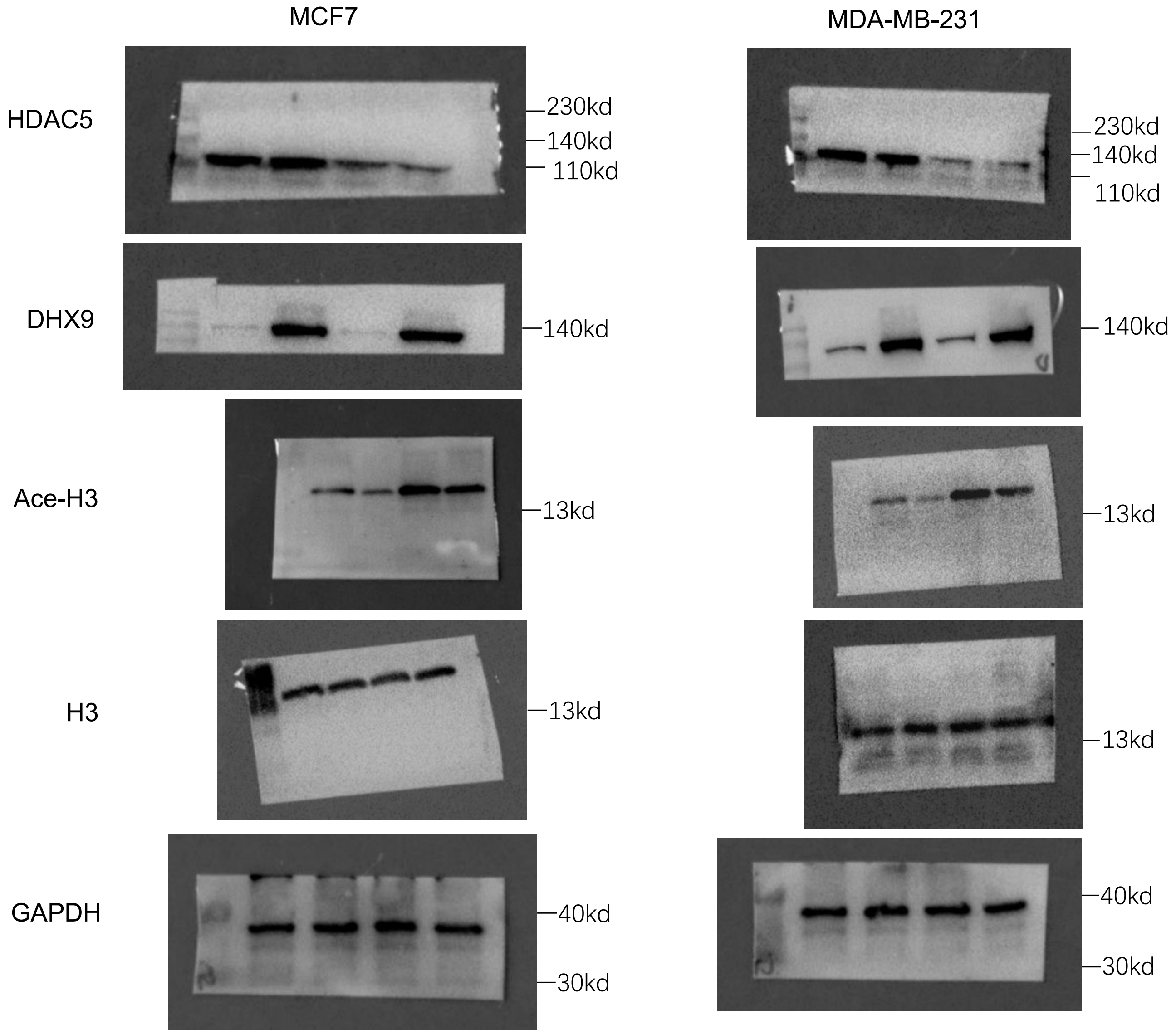
MCF7
MDA-MB-231
HDAC5
230kd
140kd
110kd
230kd
140kd
110kd
DHX9
140kd
140kd
Ace-H3
13kd
13kd
H3
13kd
13kd
GAPDH
40kd
30kd
40kd
30kd

Fig. S2A

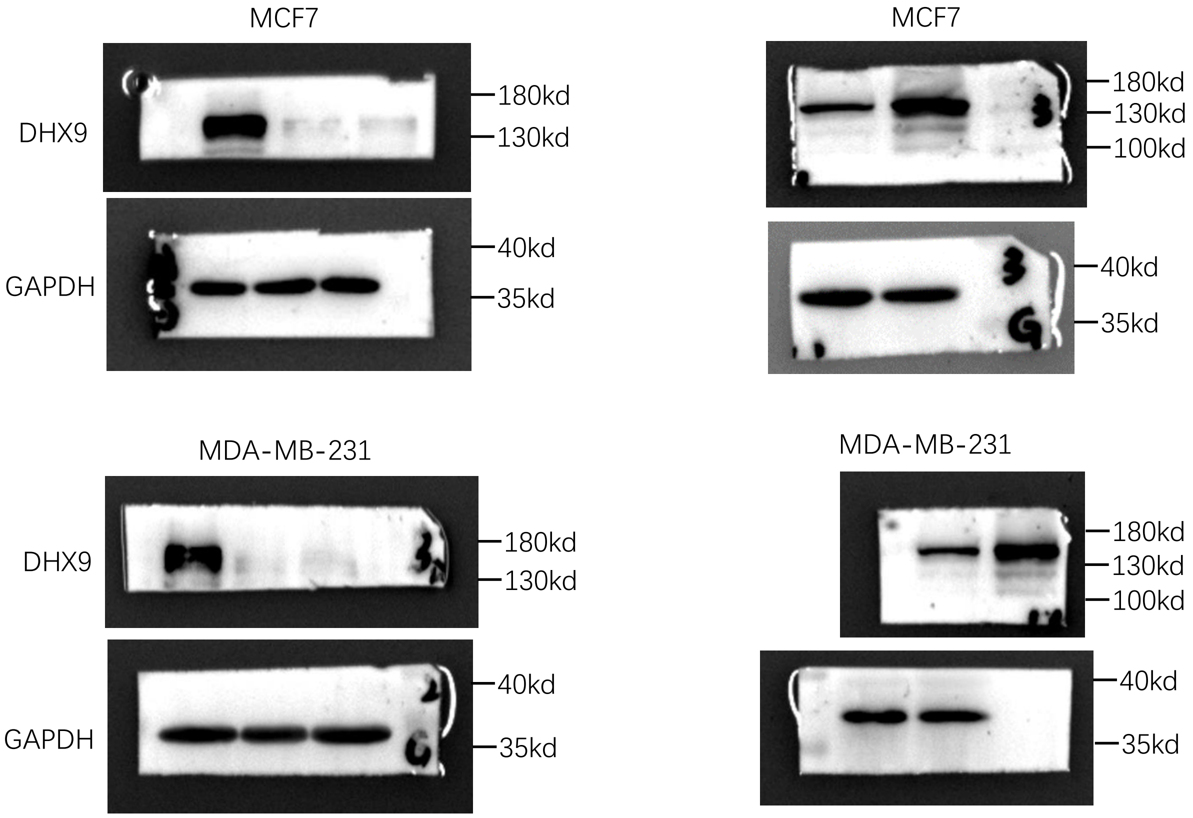

Fig. S2E

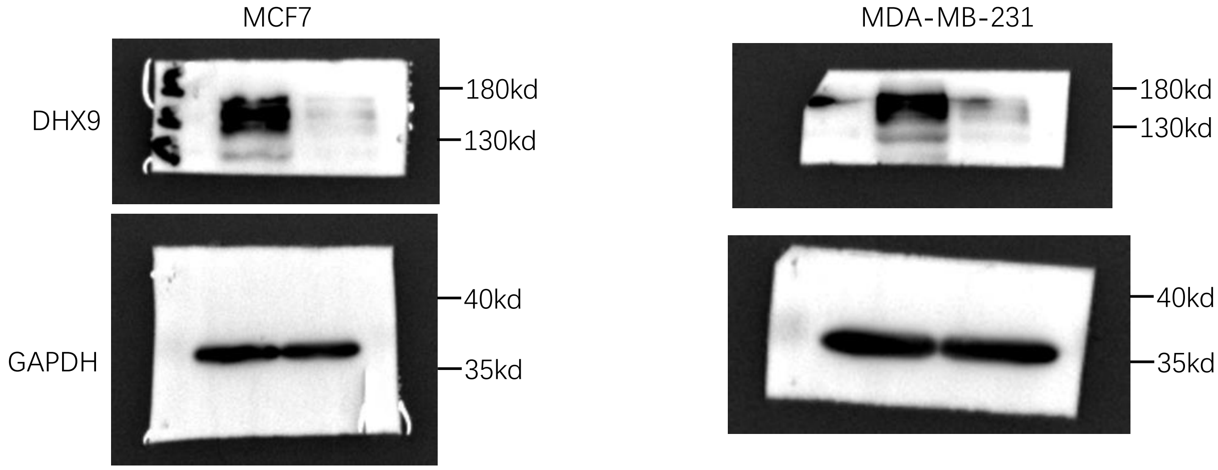

Fig. S3B

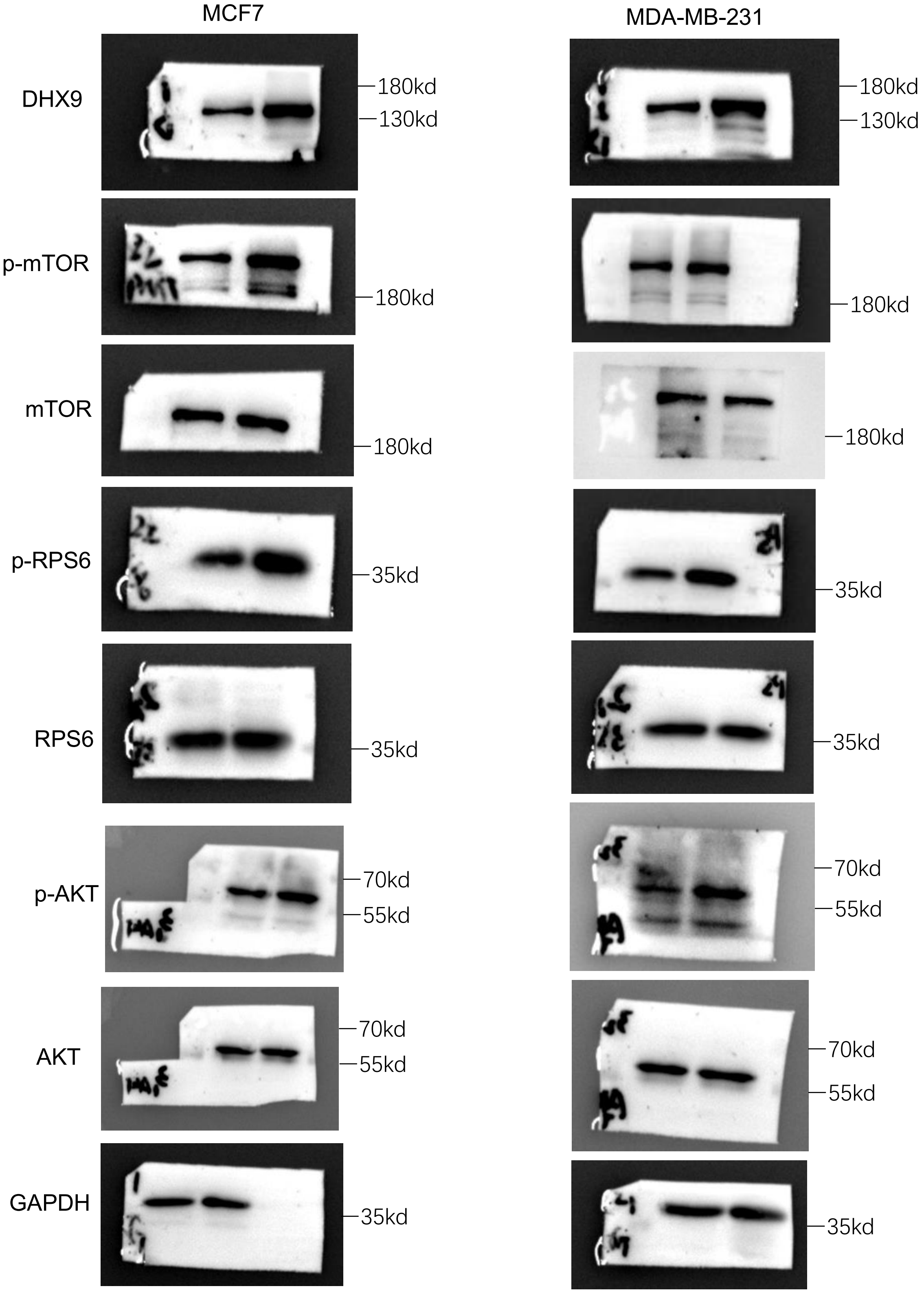
MCF7
MDA-MB-231
DHX9
180kd
130kd
180kd
130kd
p-mTOR
180kd
180kd
mTOR
180kd
180kd
p-RPS6
35kd
35kd
RPS6
35kd
35kd
p-AKT
70kd
55kd
70kd
55kd
AKT
70kd
55kd
70kd
55kd
GAPDH
35kd
35kd

Fig. S3C

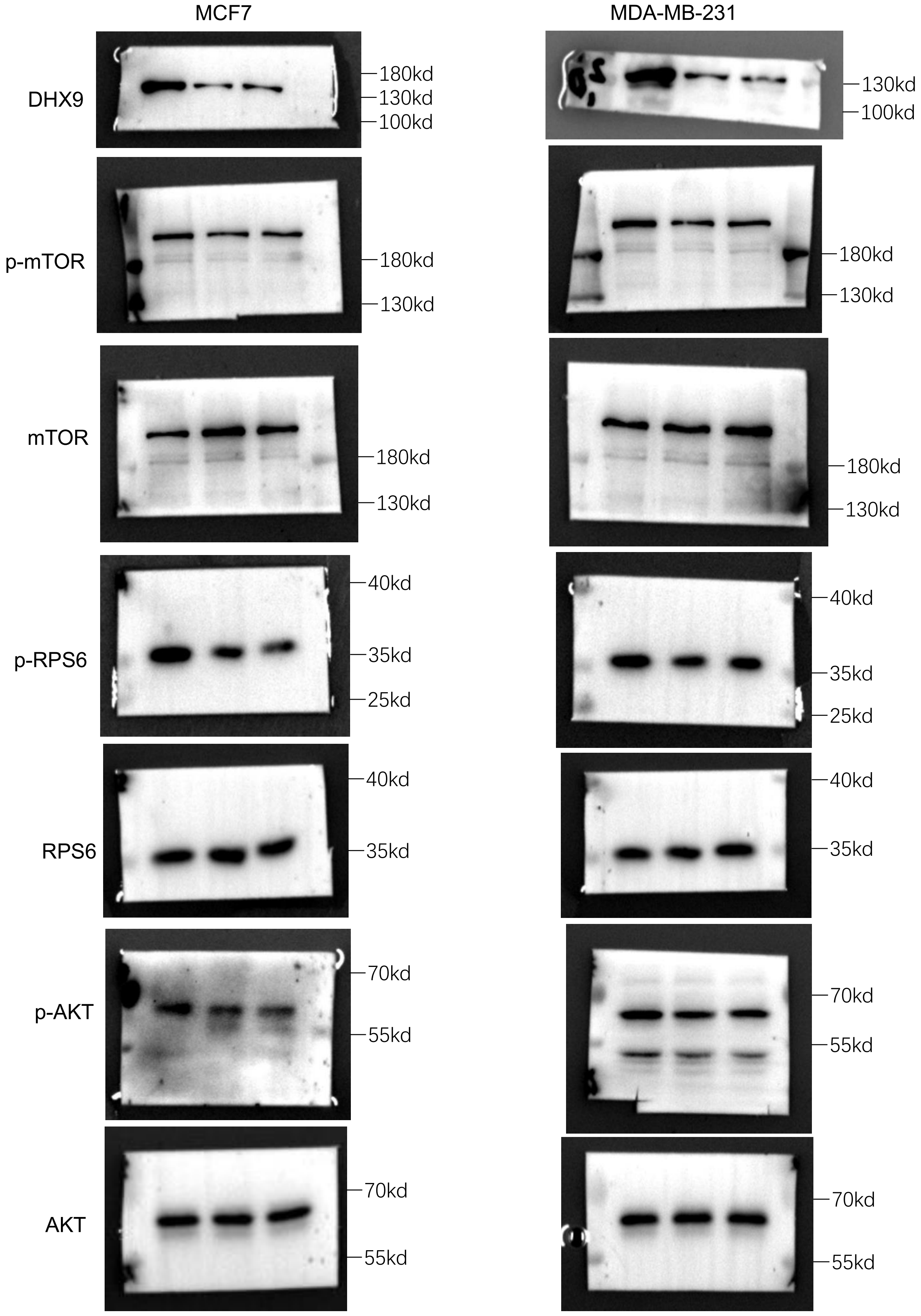
MCF7
MDA-MB-231
DHX9
180kd
130kd
100kd
130kd
100kd
p-mTOR
180kd
130kd
180kd
130kd
mTOR
180kd
130kd
180kd
130kd
p-RPS6
40kd
35kd
25kd
40kd
35kd
25kd
RPS6
40kd
35kd
40kd
35kd
p-AKT
70kd
55kd
70kd
55kd
AKT
70kd
55kd
70kd
55kd

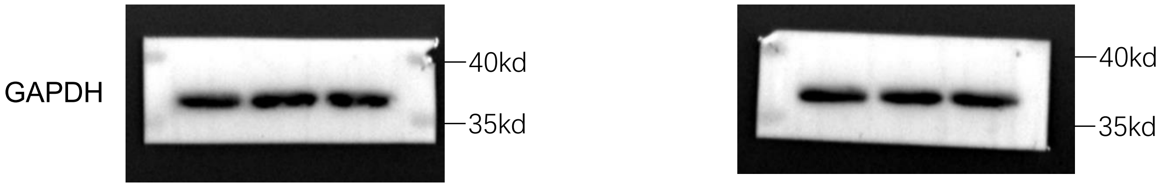

Fig. S3E

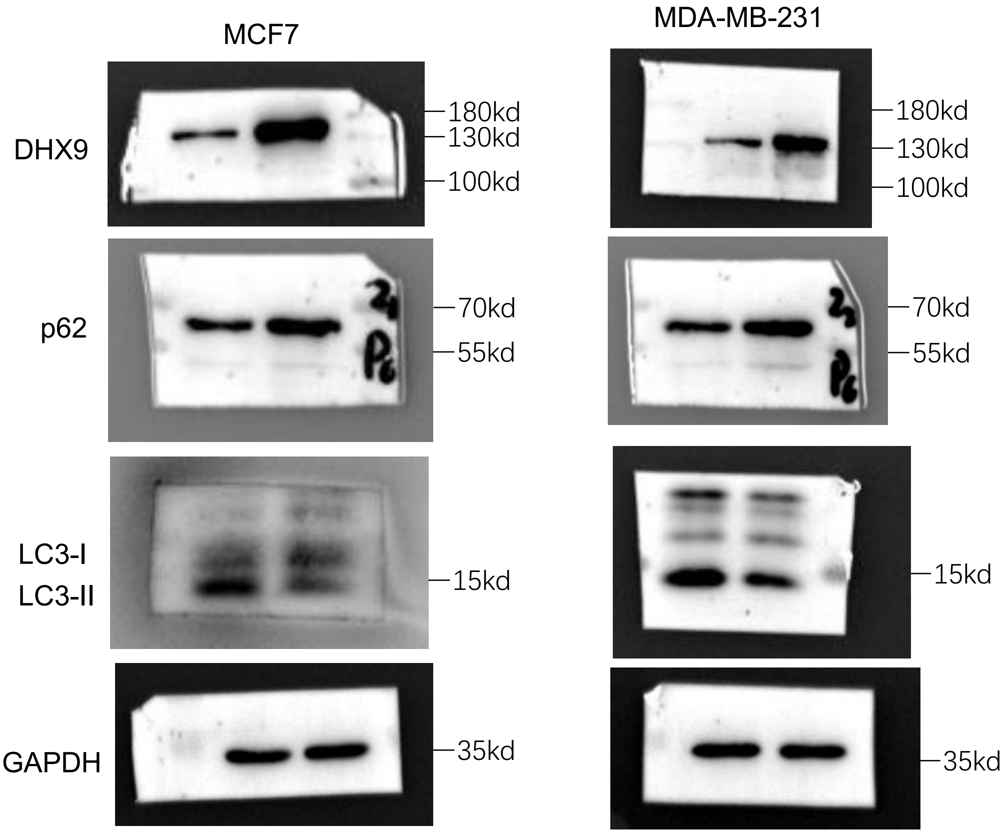

Fig. S3F

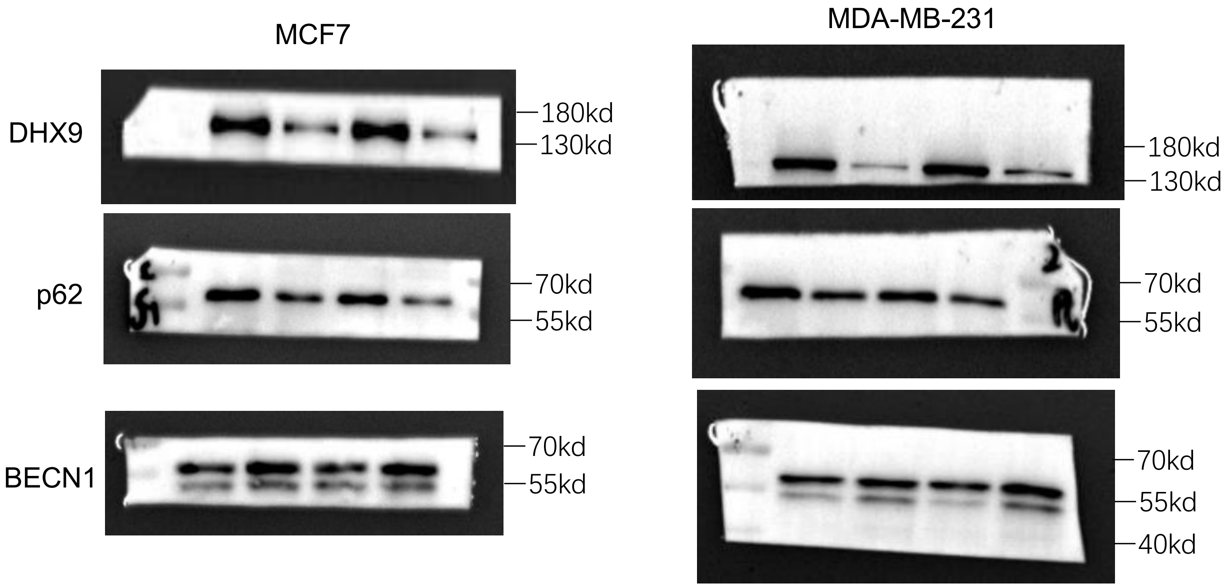

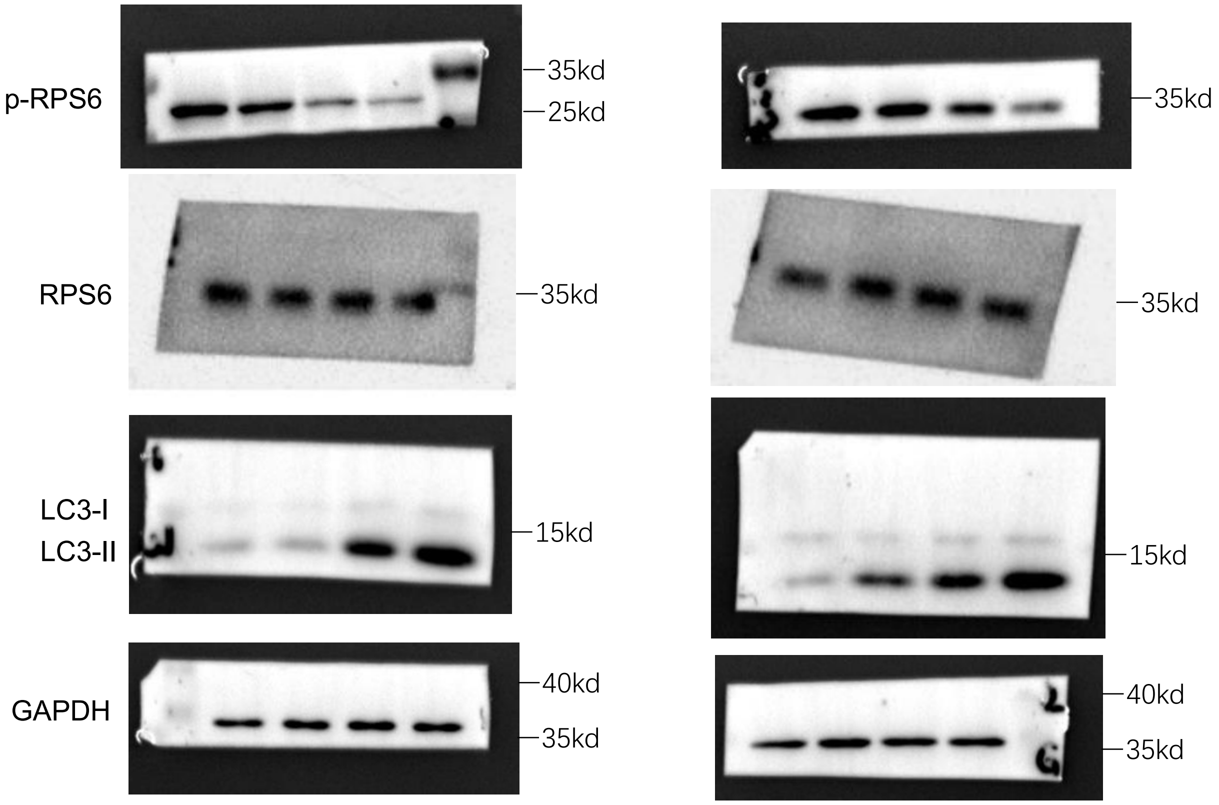
p-RPS6
35kd
25kd
35kd
RPS6
35kd
35kd
LC3-I
LC3-II
15kd
15kd
GAPDH
40kd
35kd
40kd
35kd

Fig. S4B

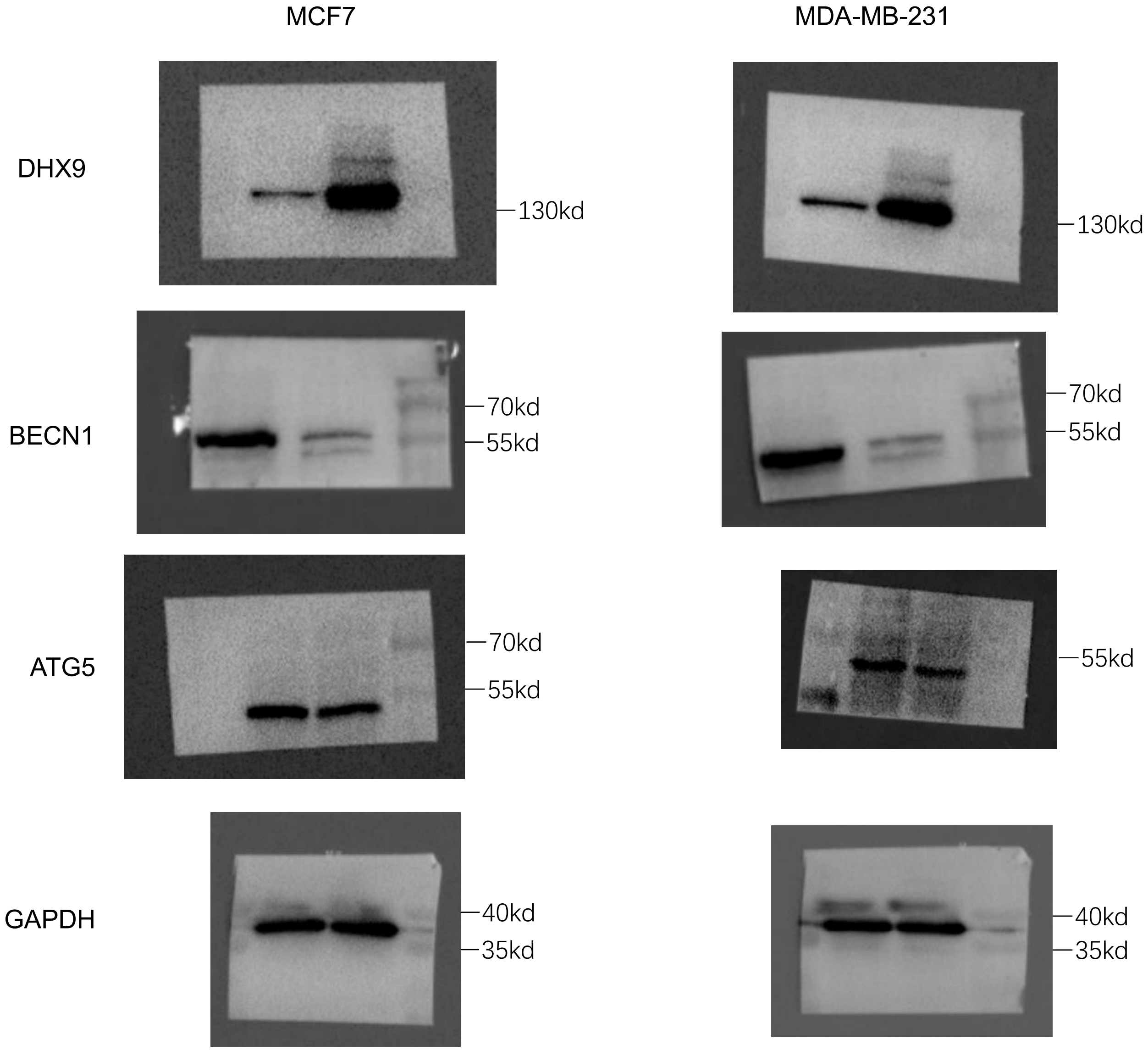

Fig S4H

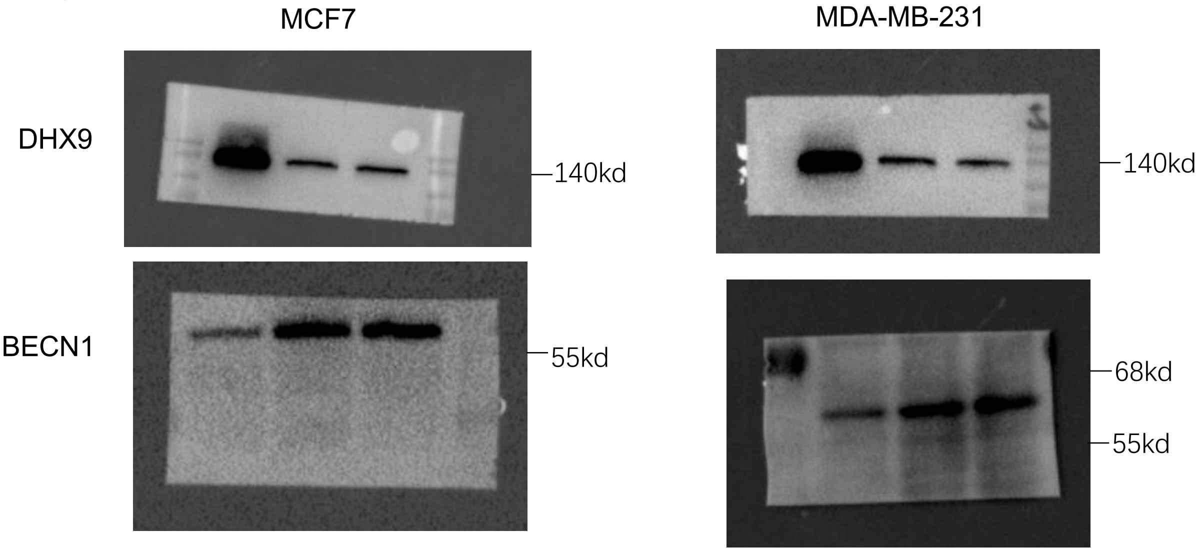

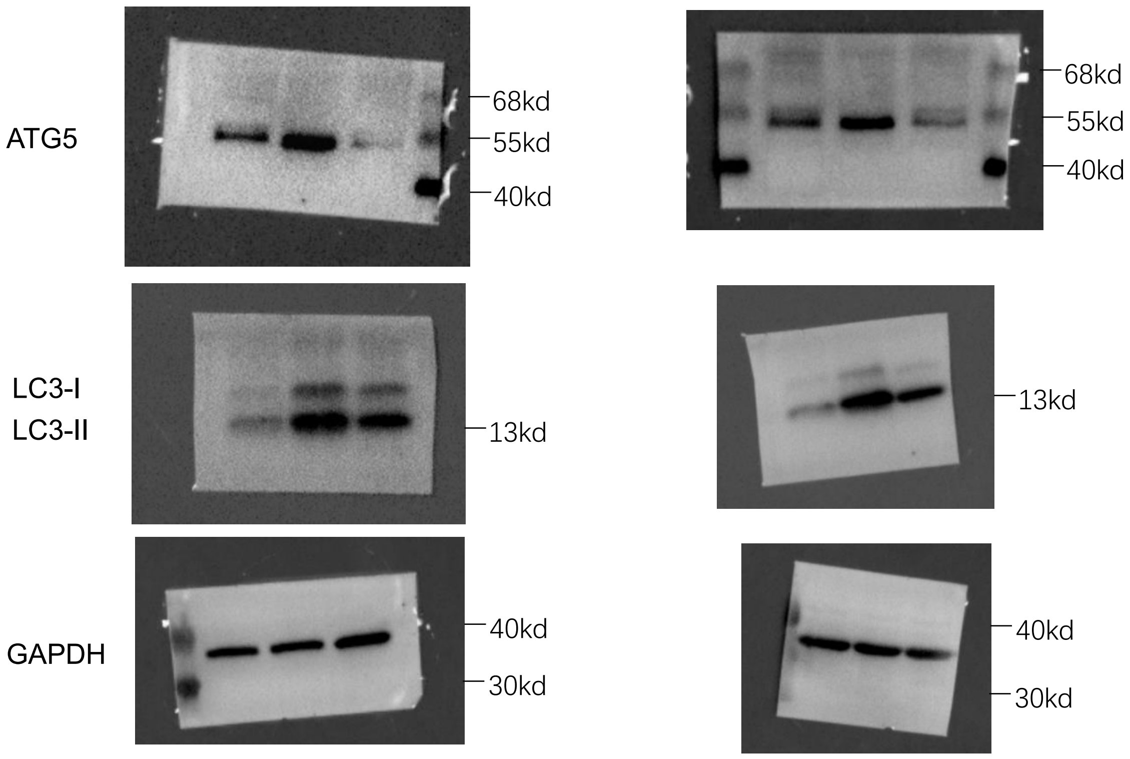
ATG5
68kd
55kd
40kd
68kd
55kd
40kd
LC3-I
LC3-II
13kd
13kd
GAPDH
40kd
30kd
40kd
30kd

Fig S4K

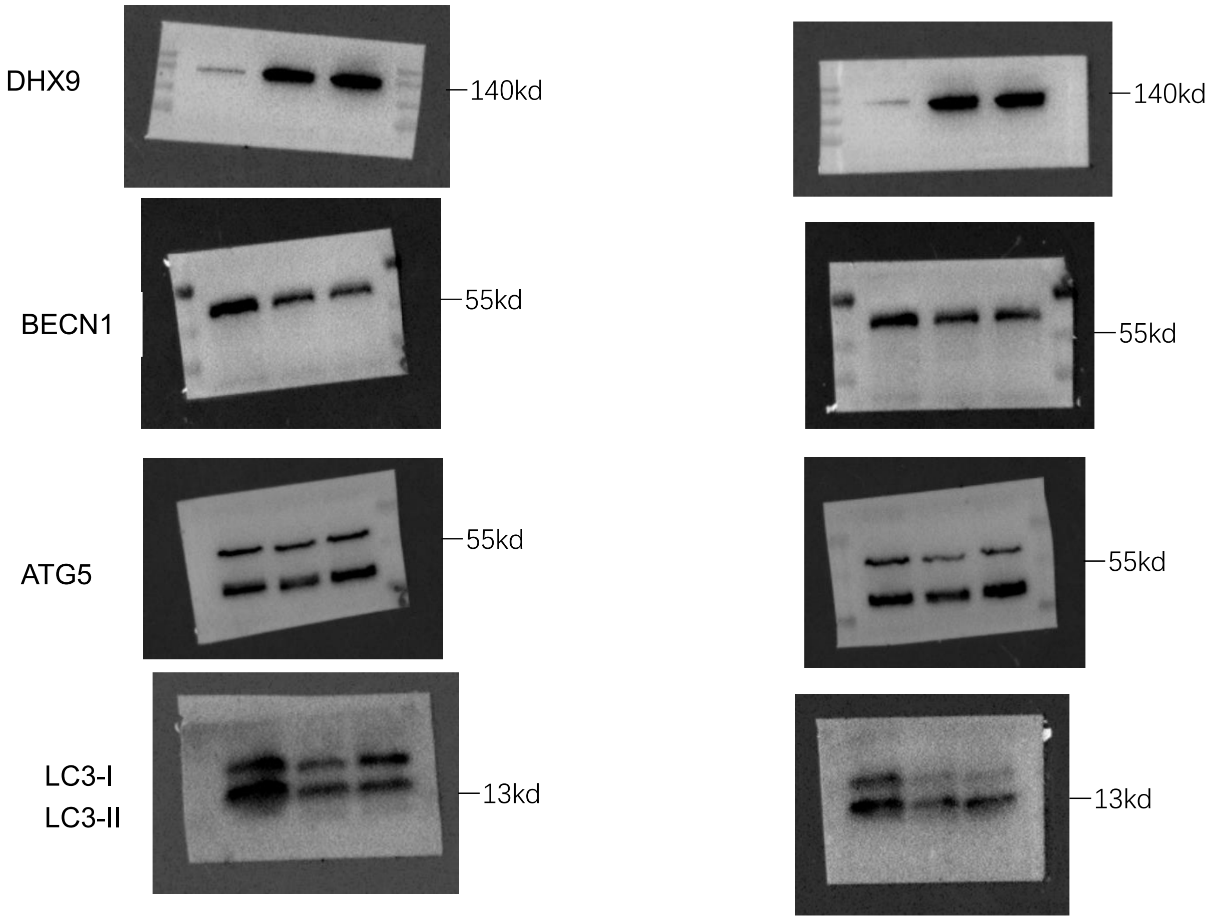
DHX9
140kd
140kd
BECN1
55kd
55kd
ATG5
55kd
55kd
LC3-I
LC3-II
13kd
13kd

GAPDH

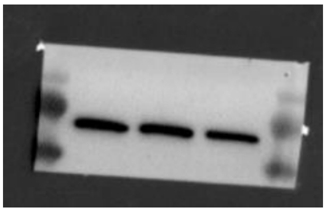

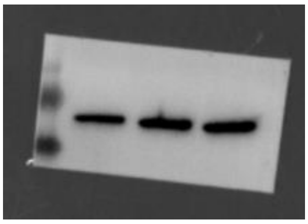

Fig S4N

DHX9

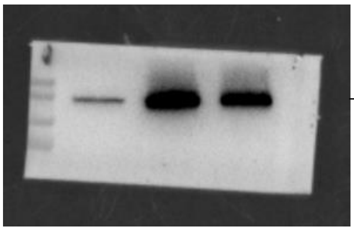

140kd

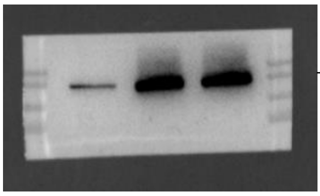

140kd

BECN1

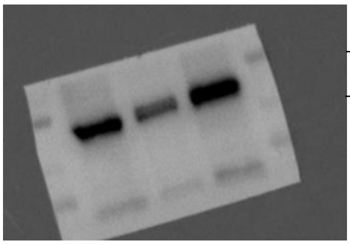

68kd
55kd

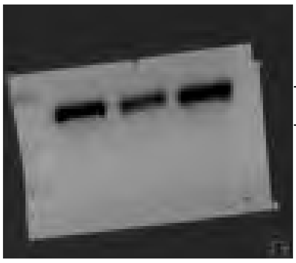

68kd
55kd

ATG5

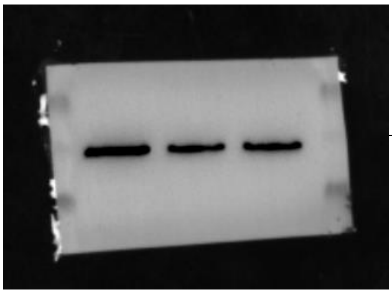

55kd

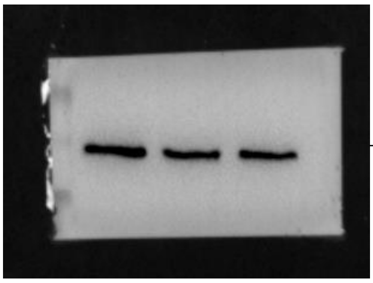

55kd

LC3-I
LC3-II

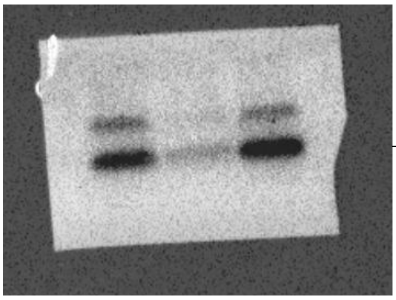

13kd

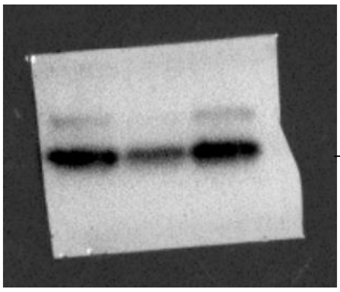

13kd

GAPDH

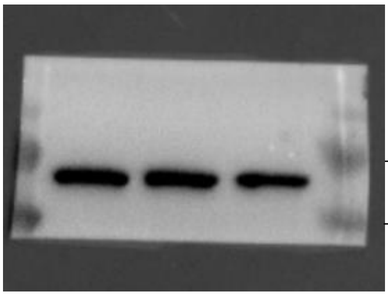

40kd
30kd

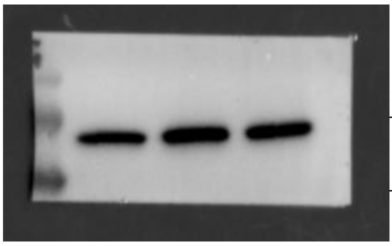

40kd
30kd

Fig. S6B

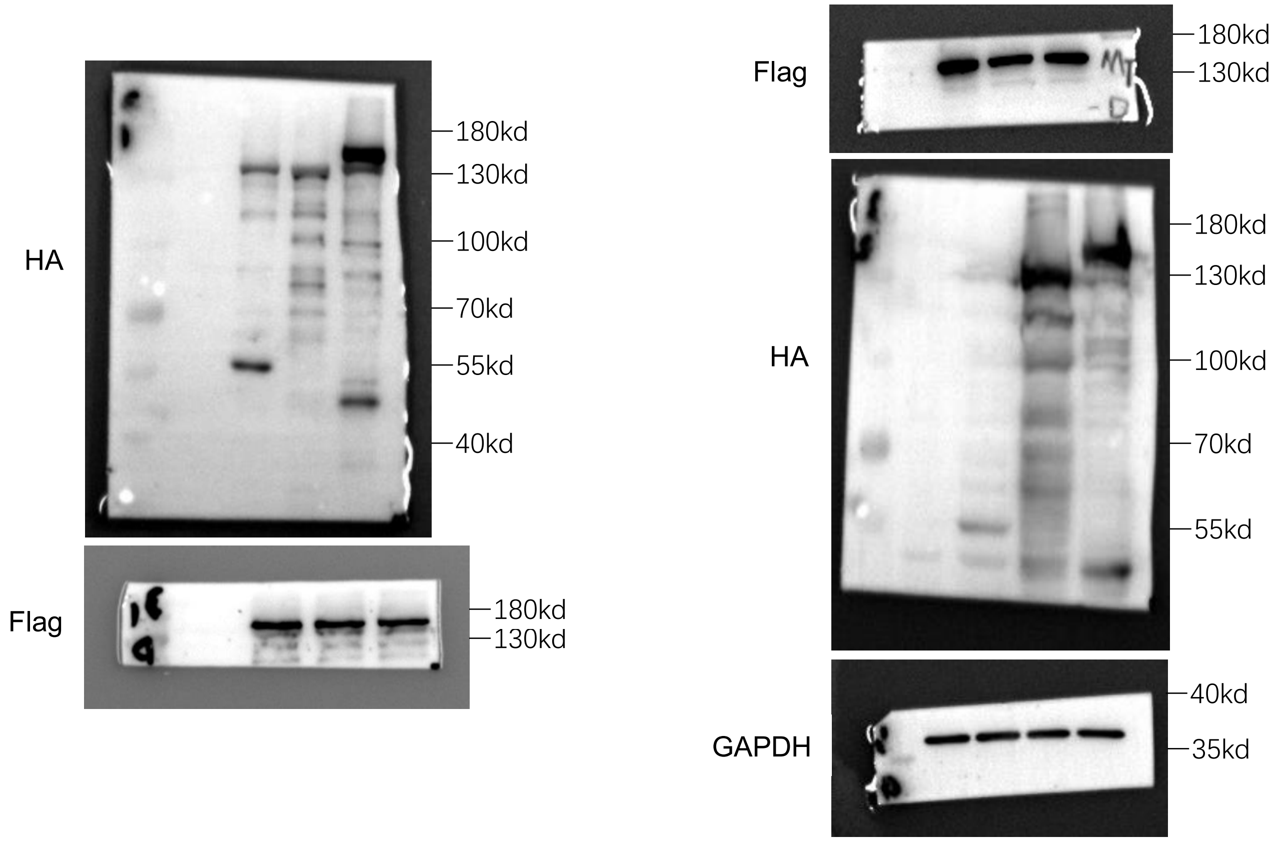

Fig. S6F

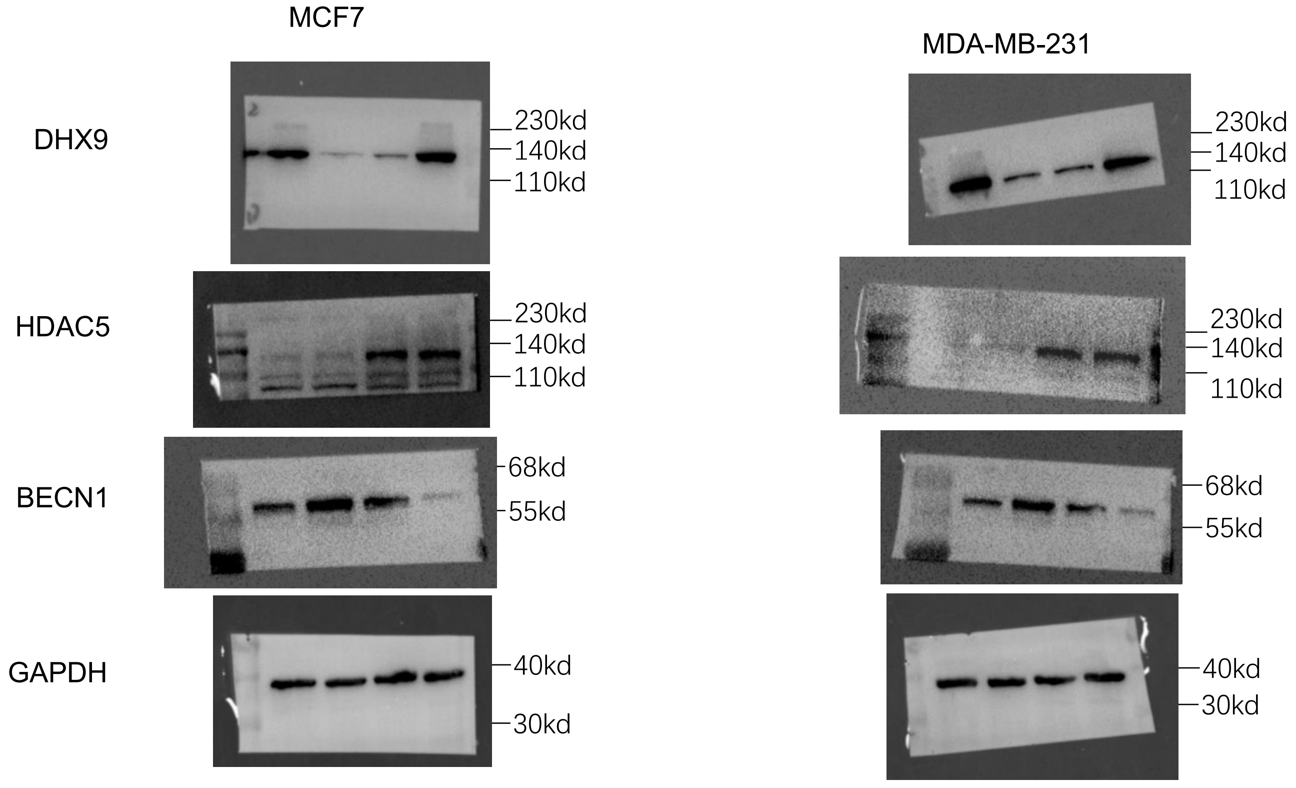

Fig. S7G

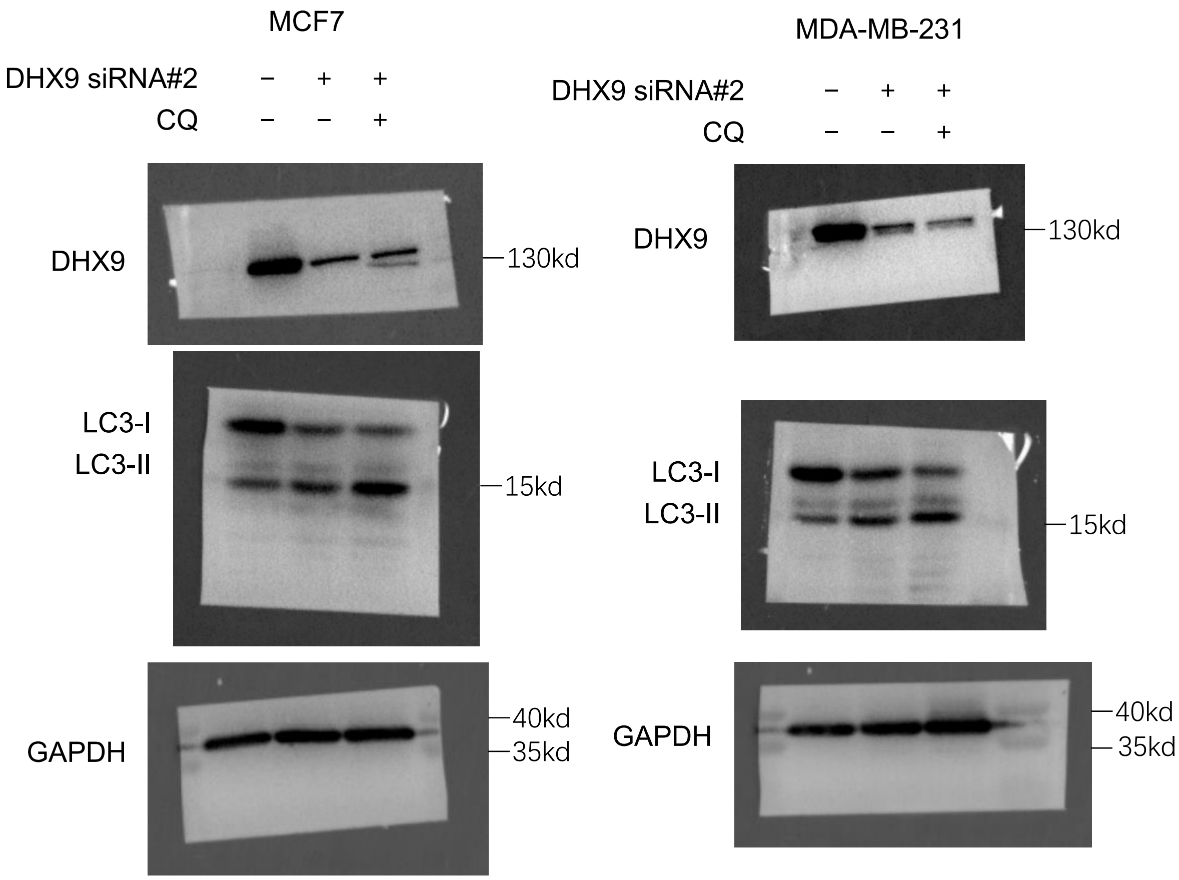
MCF7
DHX9 siRNA#2 − + +
CQ − − +
DHX9
130kd
LC3-I
LC3-II
15kd
GAPDH
40kd
35kd
MDA-MB-231
DHX9 siRNA#2 − + +
CQ − − +
DHX9
130kd
LC3-I
LC3-II
15kd
GAPDH
40kd
35kd
